# Supplementary material for: Dietary exposure to nitrites and nitrates in association with type 2 diabetes risk: Results from the NutriNet-Santé population-based cohort study
Source: PLoS Med. 2023 Jan 17;20(1):e1004149. doi: 10.1371/journal.pmed.1004149 (PMC9844911; doi:10.1371/journal.pmed.1004149)
Supplement: S1 Appendix — Method A. Methodology for identification of under-energy reporting and validation studies for the 24-h web-based dietary records. Method B. Procedures for the computation of food additive data. Method C. Incident T2D ascertainment in NutriNet-Santé and biological data assessment. Figure A. Flowchart for sample selection, NutriNet-Santé, 2009–2021. Figure B. Age distribution of study participants, NutriNet-Santé cohort, 2009–2021 (N = 104,168). Table A. Age and sex-adjusted models for associations between nitrite and nitrate exposures and T2D risk, NutriNet-Santé cohort, France, 2009–2021 (n = 104,168). Figure C. Proportional hazard assumption testing using rescaled Schoenfeld residuals. Table B. Associations between nitrite and nitrate exposures from fruit and vegetables, and red and processed meats, and T2D risk, NutriNet-Santé cohort, France, 2009–2021 (n = 104,168). Table C. Sex and antioxidant-stratified associations between exposure to nitrites/nitrates and T2D risk, NutriNet-Santé cohort, 2009–2021 (n = 104,168). Table D. Associations between nitrite and nitrate exposures and T2D risk-sensitivity analyses, NutriNet-Santé cohort, France, 2009–2021 (n = 104,168). Table E. Associations between dietary exposure to nitrates with T2D risk, adjusted stratified for mouthwash use, France, 2009–2021 (n = 25,328). Table F. Cross-sectional associations between dietary exposure to nitrites and nitrates with metabolic syndrome prevalence, NutriNet-Santé cohort, France, 2009–2021 (n = 16,450). Table G. Cause-specific associations between dietary exposure to nitrites and nitrates with mortality risk as a competing risk, NutriNet-Santé cohort, France, 2009–2021 (n = 104,168). (DOCX) [file pmed.1004149.s002.docx]

**Dietary exposure to nitrites and nitrates in association with type-2 diabetes risk:**

**Results from the NutriNet-Santé population-based cohort study**

**Srour B. *et al.***

*****S1 Appendix*****

# Method A. Methodology for identification of under-energy reporting and validation studies for the 24h web-based dietary records

## Energy under-reporting was identified using Black’s method (1,2) based on the original method developed by Goldberg et al. (3), relying on the hypothesis that energy expenditure and intake, when weight is stable, are equal. Black’s equations are based on an estimate of the person’s basal metabolic rate (BMR) calculated via Schofield’s equations (4) and taking into account sex, age, height and weight, as well as physical activity level (PAL), number of 24h records, intra-individual variabilities of reported energy intake and BMR, and intra/intervariabilities of PAL. In the present study, intra-individual coefficients of variations for BMR and PAL were fixed using the values proposed by Black et al., i.e. 8.5 % and 15%, respectively. For identifying under-reporters, the 1.55 value of PAL was used. It corresponds to the WHO value for “light” activity, which is the probable minimum energy requirement for a normally active but sedentary individual (not sick, disabled or frail elderly). A higher value might have exaggerated the extent of under-reporting. Some under-reporting individuals were not excluded if their reported energy intake, initially estimated abnormally low, was found to be likely in case of recent weight variation or reported practice of weight-loss restrictive diet or proactive statement of the participant that he/she ate less than usual on the day of the dietary record. In this study 21,708 participants (corresponding to 16.7% of the subjects) were considered as under-energy reporters and were excluded from the study. This proportion of under-reporters is common, for instance in the nationally representative INCA 3 study conducted in 2016 by the French Food Safety Agency (5) 18% of adults participants were identified as under-reporters using the Black method. Comparison between under-reporters and included participants is presented in the table below.

|  | **Under-energy reporters**  (n=21,796) | **Included participants**  (n=104,168) |
| --- | --- | --- |
| **Age** | 44.7 (14.9) | 42.7 (14.5) |
| **Sex, women** (%) | 16,042 (73.6%) | 82,474 (79.1%) |
| **BMI** (kg/m2) | 25.7 (5.9) | 23.7 (4.3) |
| **Number of dietary records** | 5.7 (3.1) | 5.6 (3.0) |
| **Energy intake without alcohol** (kcal/day) | 1,326.7 (420.9) | 1,846.2 (451.6) |
| **Monthly income** (Euros) | 2,637.5 (2145.3) | 3,047 (2282.1) |
| **Smoking status (%)** |  |  |
| Current smokers | 3,691 (16.9%) | 14,004 (14.3%) |
| Former smokers | 9,183 (42.1%) | 39,093 (40.0%) |
| Never smokers | 8,922 (40.9%) | 44,569 (45.6%) |
| **Alcohol intake (g/day)** | 5.0 (9.3) | 7.8 (11.8) |
| **Physical activity level (%)** |  |  |
| High | 7,738 (35.5%) | 29,293 (32.6%) |
| Moderate | 9,177 (40.1%) | 38,649 (43.0%) |
| Low | 5,319 (24.4%) | 21,896 (24.4%) |
| **Educational level (%)** |  |  |
| < High school degree | 5,885 (27.0%) | 17,035 (16.5%) |
| <2 years after high school | 3,946 (18.1%) | 16,287 (15.8%) |
| ≥2 years after high school | 11,967 (54.9%) | 69,952 (67.7%) |

Values are: mean (SD) for continuous variables. All p-values <0.001

# Method B: Procedures for the computation of food additive data

### Description of the databases for qualitative composition information

### Open Food Facts

Open Food Facts (OFF) (http://world.openfoodfacts.org/) is an open collaborative database of food products marketed worldwide under the Open Database License (ODBL). It contains data on hundreds of thousands of products (161,885 products with details of the list of ingredients). The initiative started in France in 2012, offering extensive coverage of the French food market, and a growing number of products are now available for other countries around the world. Contributors (citizens and active OFF contributors) are constantly adding products to this popular database by scanning the barcode and sending photographs of the packaging (citizen participative research). The information is processed automatically by artificial intelligence to retrieve different information for each product, such as trade name, brand, list of ingredients (including food additives) and nutritional composition. As food product formulations may change, compositions are regularly updated when they are re-registered by consumers. Each product is assigned one or more food categories and is identified by the Global Trade Item Number (GTIN) embedded in the barcode.

### OQALI

Oqali (French Food Quality Observatory, <https://www.oqali.fr/oqali_eng/>) has been carrying out studies to characterize the nutritional quality of the food supply of processed products, both in terms of the information available on the packaging and the nutritional composition of the products. It is jointly implemented by Anses (the French Agency for Food, Environmental and Occupational Health & Safety) and INRAE (Institut National de la Recherche pour l'Agriculture, l'alimentation et l'Environnement). The database includes 49,854 products (studied between 2008 and 2019) from 30 food sectors and from 5 market segments (specialist retailers, national brands, private labels, entry-level private labels and hard discount), identified by barcode. The database contains information on ingredient list, including food additives.

### GNPD

Mintel Global New Products Database (GNPD, https://www.mintel.com/global-new-products-database) is an online international database that registers food innovations (new product launched on the market, reformulation or packaging changes). Over five million records from more than 80 countries provide product ingredients, nutrition facts, packaging, distribution and pricing information. After extraction of the database in 2019, detection of food additives labels or numbers was performed based on the ingredient list. A total of 51,599 products were included.

### Merging NutriNet-Santé food consumption data with and Open Food Facts, Oqali and GNPD qualitative composition data

First, NutriNet-Santé generic food or beverage items were merged with the OFF categories by a trained team of three dietitians, one data-manager and one computer scientist who followed strict procedures as described below in order to reassure a high level of standardization in the food matching with the additives data. Only the OFF products with an available ingredient list were kept. After elimination of staple items generally consumed without brand names (such as fresh vegetables), 1,557 generic NutriNet food items (e.g. “chocolate cookie”) were matched with OFF categories. Two types of matches were performed:

- **Direct matches** between a generic NutriNet item and an OFF category (for 676 NutriNet items)

- **More complex matches** (with multiple categories or a category and a keyword). Example: the NutriNet item "Plain Cereal Bars" was matched with the OFF products of the category "Cereal Bars" and containing the keyword "plain" in their name.

Then, NutriNet-Santé and OFF food items were matched at the brand/commercial name level. Indeed, in NutriNet-Santé 24h dietary records, participants declare the brand of the product consumed by checking from a list of predefined brands or by typing in plain text. A table of correspondence between the predefined brands in NutriNet and the different OFF brands (n=20,000) was therefore created by the dietitians of the team. For the brands entered in clear text, each entry was cleaned and matched with an OFF brand. When the brand was missing, imputations were made considering (in the following order):

1) the brand most frequently declared by the participant for the same NutriNet food item (in other meals of other days of 24-hour records)

2) participant's answers to a questionnaire specifically designed to collect information on the most frequently consumed brands for each product category

3) the brand most declared by the Nutrinaute (as in 1), but based on the food category of the brand questionnaire (which contains several NutriNet items).

Similarly, NutriNet-Santé data was matched with the OQALI and GNPD databases.

When several industrial food products of food additive composition were possible candidates for a commercial food declared in NutriNet, databases were prioritized as follows: Oqali (national and official database from the French food safety authority), then Open Food Facts (very wide coverage of the French market) and lastly GNPD (as an international database, ingredients are registered in English which may lower the sensitivity of additive detection in the list). Moreover, to account for possible reformulations, products with a date of inclusion in the databases within +/- 1 year of the date of consumption were considered (dynamic matching).

### Quantitative data (doses of food additives)

No comprehensive quantitative database was available because information on the amount of each additive in a given product is not mentioned on its label. The quantitative composition of additives has therefore been derived from several sources. Firstly, ad-hoc laboratory assays have been carried out, prioritizing the most consumed additives and those with suspected health effects. The selected food products were the main vectors of these additives in our study population. 2,677 assays were performed. These assays were performed by Mérieux and Eurofins firms and the French DGCCRF (the General Directorate for Competition Policy, Consumer Affairs and Fraud Control) public laboratories. We also retrieved data from ad hoc dosing previously commended by the consumer association "UFC Que Choisir" which covered 39 additives and 1,721 products. These data from laboratory assays were used to impute doses: e.g. for a NutriNet food item of a specific brand that contains a specific additive but for which no assay data was available, all values of assays corresponding to the same generic item but to other brands were averaged. Besides, during the re-evaluation of food additives, the European Food Safety Authority (EFSA) regularly carries out exposure simulations based on the doses of additives in food products communicated by manufacturers across Europe. These doses (at the generic food item level – no brand data) have been used when no dose was available from laboratory assays. When EFSA data was missing on usage doses, the maximum levels authorized by the regulation were used (6). Last, quantitative additive data from the Codex General Standard for Food Additives (GSFA) (7) were used. Figure 1 illustrates the decision tree for the computation of food additive doses.


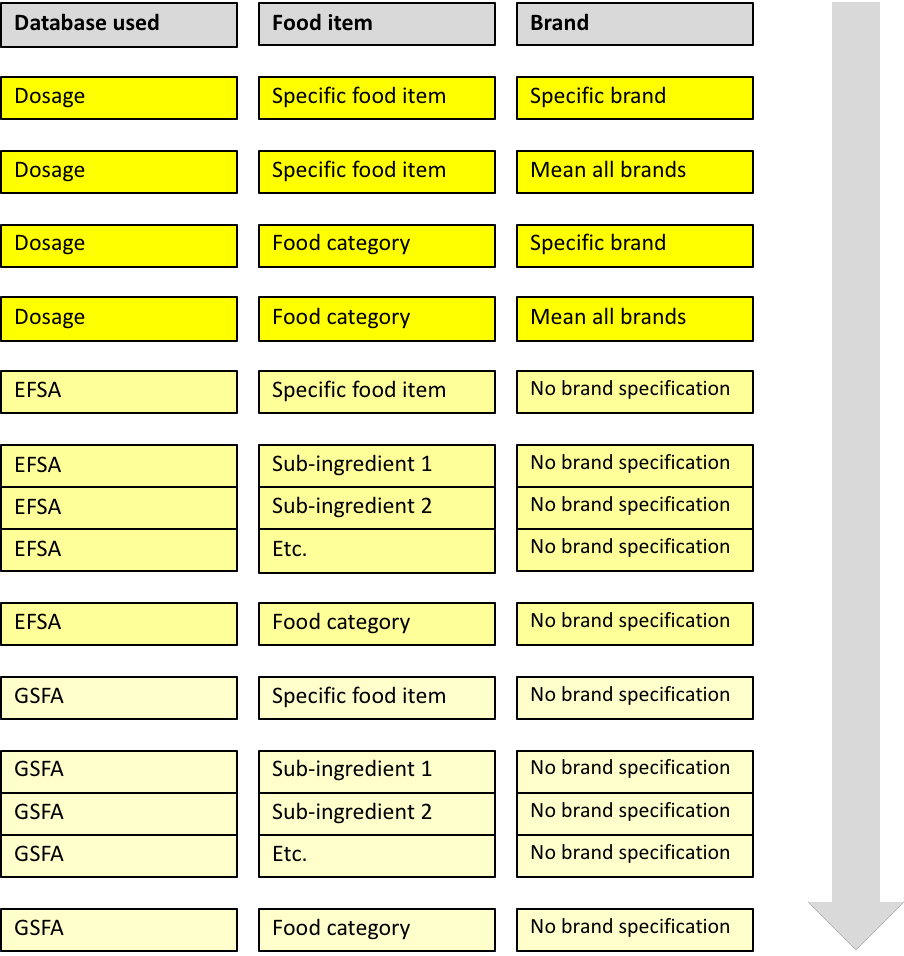


Figure 1: Decision tree for food additive doses computation

# Method C: Incident type-2 diabetes ascertainement in NutriNet-Santé and biological data assessment

Participants were asked to declare major health events though the yearly health questionnaire, through a specific health check-up questionnaire every six months, or at any time through a specific interface on the study website. They were also asked to declare all currently taken medications and treatments via the check-up and yearly questionnaires. A search engine with embedded exhaustive Vidal drug database is used to facilitate medication data entry for the participants. Besides, our research team was the first in France to obtain the authorization by Decree in the Council of State (n°2013-175) to link data from our general population-based cohorts to medico-administrative databases of the National health insurance (SNIIRAM database). Thus, data from the NutriNet-Santé cohort are linked every year to medico-administrative databases of the SNIIRAM, providing detailed information about the reimbursement of medication and medical consultations. Participants have been informed about this linkage and had the right to revoke their given consent at any time on a dedicated interface on the study website.

Regarding T2D specifically: all 969 cases were primarily detected through the declaration by the participants of a T2D diagnosed by a physician and/or diabetes medication use, in follow-up questionnaires. The questions were: “Have you been diagnosed with T2D (if yes, indicate the date of diagnosis)” and “Are you treated for T2D?”. ATC codes considered for T2D medication were A10AB01, A10AB03, A10AB04, A10AB05, A10AB06, A10AC01, A10AC03, A10AC04, A10AD01, A10AD03, A10AD04, A10AD05, A10AE01, A10AE02, A10AE03, A10AE04, A10AE05, A10AE30, A10BA02, A10BB01, A10BB03, A10BB04, A10BB06, A10BB07, A10BB09, A10BB12, A10BD02, A10BD03, A10BD05, A10BD07, A10BD08, A10BD10, A10BD15, A10BD16, A10BF01, A10BF02, A10BG02, A10BG03, A10BH01, A10BH02, A10BH03, A10BX02, A10BX04, A10BX07, A10BX09, A10BX10, A10BX11, A10BX12.

Following a T2D diagnosis and/or medication declaration, two additional sources of information were considered for confirmation. First, the linkage with the SNIIRAM National health insurance database allowed confirming 85.7% of investigated cases (ICD-10 codes E11). The sensitivity of SNIIRAM databases is not optimal if used without self-report data: of note, about 10-15% of the French population is covered by other social security regimen and would not be correctly captured by the SNIIRAM databases. Besides, the centralization of SNIIRAM data might take up to a year, leading to delays between reported T2D information and health insurance data.

Second, as regards biological samples, a subsample of participants (n=19,800) were invited to a clinical examination, during which they provided blood and urine samples (details below). Among the participants who provided blood sample during the clinical/biological examination, 232 had elevated fasting blood glucose (i.e. >1.26 g/L). Among them, 85.3% had consistently declared a T2D diagnosis and/or medication. Elevated blood glucose alone (i.e., without any declaration of T2D diagnosis or treatment), especially if issued from a single biological measurement, was not considered specific enough to classify the participant as a T2D case, as per the guidelines of the *French Haute Autorité de Santé* (8)*.*

**Biological data assessment**

During a clinical examination visit, blood samples were collected after at least a 6-h fast period and centralized and analyzed at a single laboratory (IRSA, Tours, France). Total serum cholesterol (cholesterol oxidase C8000, Abbott), high-density protein cholesterol (HDL-cholesterol) (direct accelerator C8000, Abbott), serum triglycerides (glycerol kinase C8000, Abbott), and fasting blood glucose were measured (hexokinase on C 8000 automat, Abbott, Suresnes, France). Low density protein cholesterol (LDL-cholesterol) was calculated using the Friedwald formula**.**

# Figure A: Flowchart for sample selection, NutriNet-Santé, 2009-2021


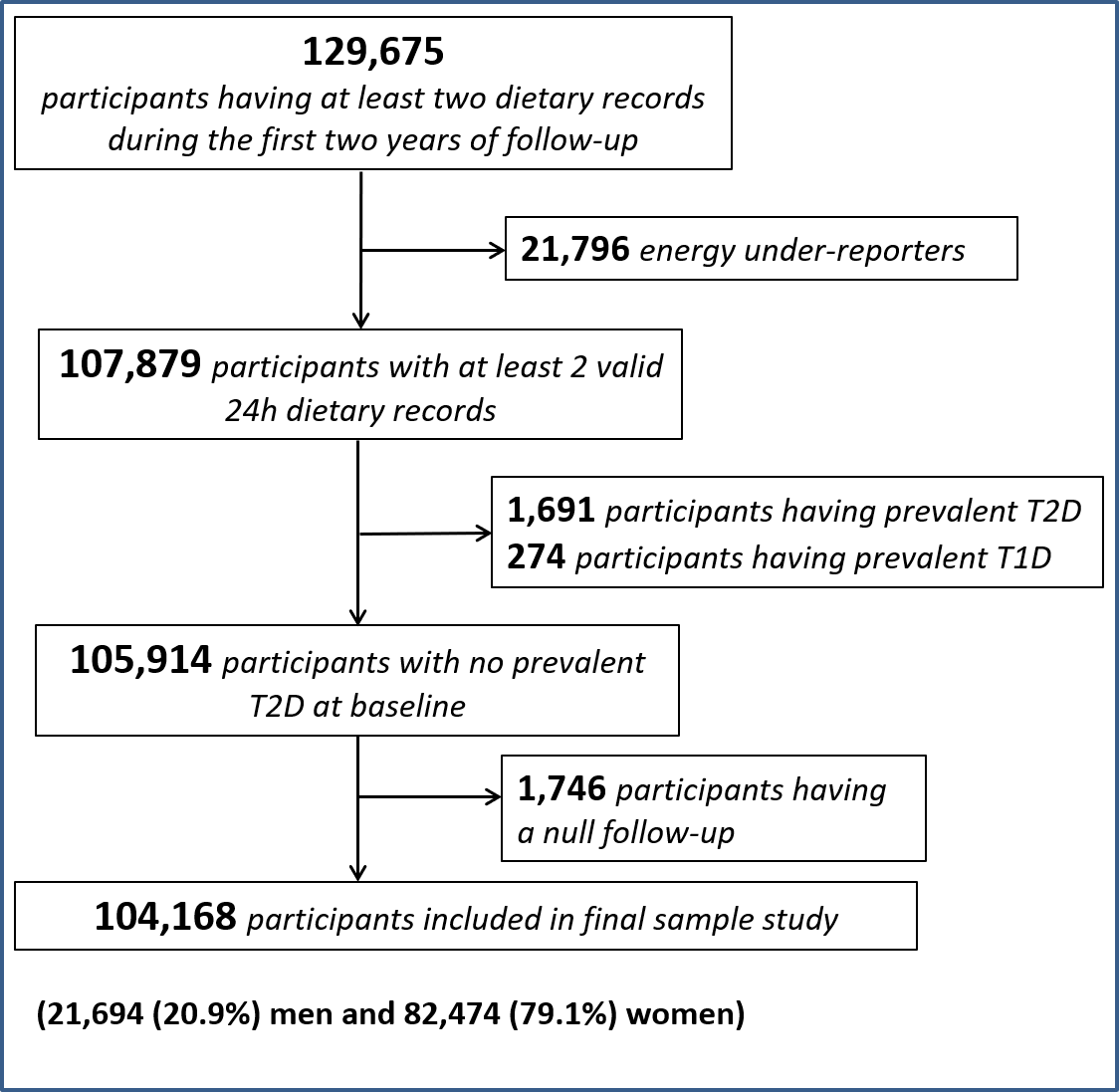


**Figure B: Age distribution of study participants, NutriNet-Santé cohort, 2009-2021 (N=104,168)**


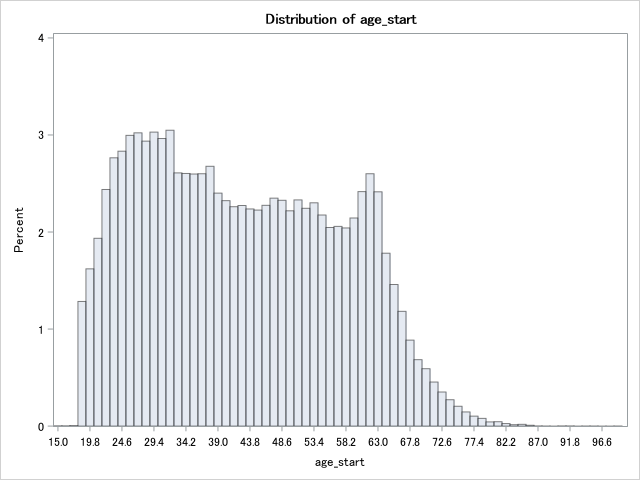


**Table A: Age and sex-adjusted models for associations between nitrite and nitrate exposures and type 2 diabetes risk, NutriNet-Santé cohort, France, 2009-2021 (n=104,168)**

| **Exposure** |  | **Sex-specific categories of exposure** | | | **P_trend_** |
| --- | --- | --- | --- | --- | --- |
|  |  | **1** | **2** | **3** |  |
| **Total nitrites** | N cases / person-years | 210/219252 | 327/246955 | 432/243915 | <0.001 |
|  | HR (95% CI) | Ref | 1.14 (0.96-1.36) | 1.54 (1.31-1.82) |  |
| Foods and water-originated nitrites | N cases / person-years | 203/218439 | 347/248755 | 419/242927 | <0.001 |
|  | HR (95% CI) | Ref | 1.19 (1.00-1.41) | 1.49 (1.26-1.76) |  |
| Additives-originated nitrites | N cases / person-years | 154/153197 | 367/287369 | 448/269555 | <0.001 |
|  | HR (95% CI) | Ref | 1.16 (0.96-1.39) | 1.80 (1.50-2.17) |  |
| Sodium nitrite (e250) | N cases / person-years | 154/153593 | 365/287578 | 450/268950 | <0.001 |
|  | HR (95% CI) | Ref | 1.15 (0.95-1.39) | 1.82 (1.51-2.18) |  |
| **Total nitrates** | N cases / person-years | 238/216427 | 322/248759 | 409/244936 | 0.5 |
|  | HR (95% CI) | Ref | 0.87 (0.74-1.04) | 0.93 (0.79-1.09) |  |
| Foods and water-originated nitrates | N cases / person-years | 239/216489 | 321/248675 | 409/244958 | 0.5 |
|  | HR (95% CI) | Ref | 0.87 (0.73-1.03) | 0.92 (0.79-1.09) |  |
| Additives-originated nitrates | N cases / person-years | 580/454464 | 210/138880 | 179/116777 | 0.02 |
|  | HR (95% CI) | Ref | 1.05 (0.89-1.23) | 1.22 (1.03-1.44) |  |
| Potassium nitrate (e252) | N cases / person-years | 580/454500 | 210/138895 | 179/116726 | 0.02 |
|  | HR (95% CI) | Ref | 1.05 (0.89-1.23) | 1.22 (1.03-1.45) |  |

HR: hazard ratio; 95% CI: 95% confidence interval

Cox proportional hazard model were adjusted for: age (time scale) and sex.

For exposure to total nitrites and nitrates and foods and water-originated nitrites and nitrates, sex-specific tertiles of exposure were defined. Cutoffs were: 4.03mg/d and 5.55mg/d in women and 5.18mg/d and 7.44mg/d in men for total nitrites, 150.09mg/d and 233.89mg/d in women and 162.11mg/d and 251.59mg/d in men for total nitrates, 3.83mg/d and 5.29mg/d in women and 4.92mg/d and 7.07mg/d in men for foods and water-originated nitrites, 149.91mg/d and 233.75mg/d in women and 161.94mg/d and 251.32mg/d in men for foods and water-originated nitrates.

For additives-originated nitrites and nitrates, 3 categories of exposure were defined: non-exposed, lower exposure and higher exposure (separated by sex-specific median among exposed participants). Cut-offs were: 0.19mg/d in women 0.25mg/d in men for additives-originated nitrites, 0.36mg/d in women and 0.46mg/d in men for additives originated nitrates, 0.19mg/d in women and 0.25mg/d in men for sodium nitrite (e250), 0.36mg/d in women and 0.46mg/d in men for potassium nitrate (e252).

# Figure C: Proportional hazard assumption testing using rescaled Schoenfeld residuals


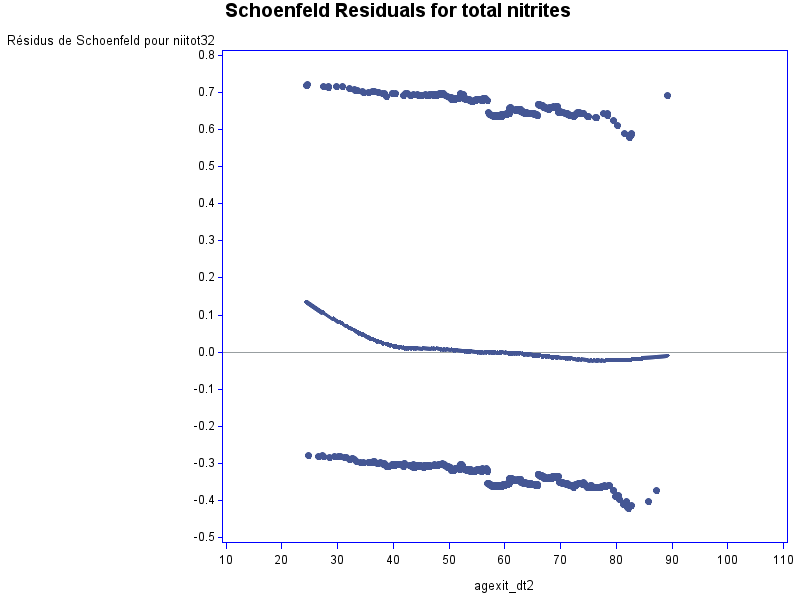


P-value for Pearson correlation between residuals and timescale =0.28

**Schoenfeld Residuals for foods and water-originated nitrites**


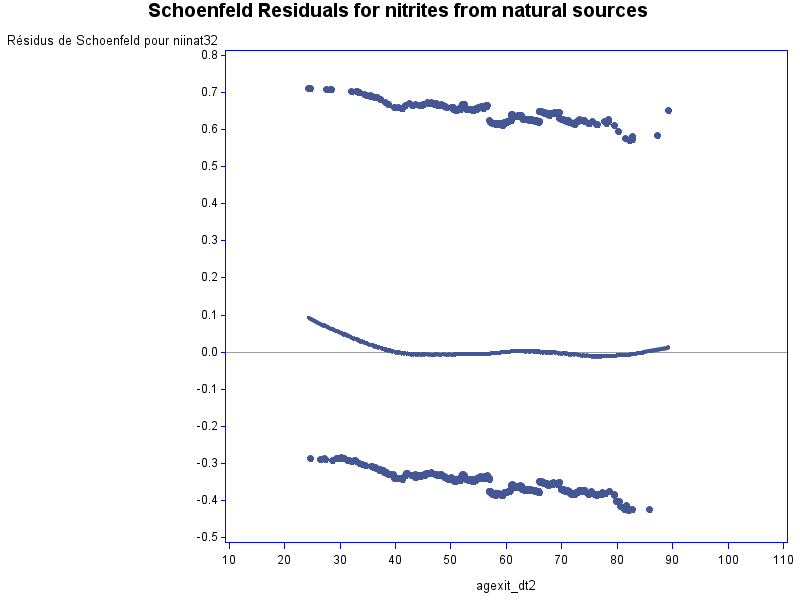


P-value for Pearson correlation between residuals and timescale =0.74

**Schoenfeld Residuals for additives-originated nitrites**


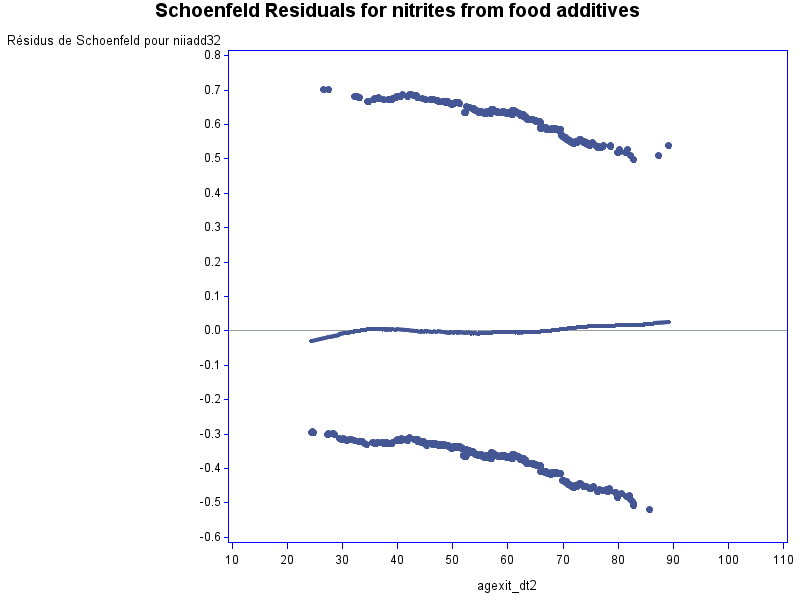


P-value for Pearson correlation between residuals and timescale =0.85


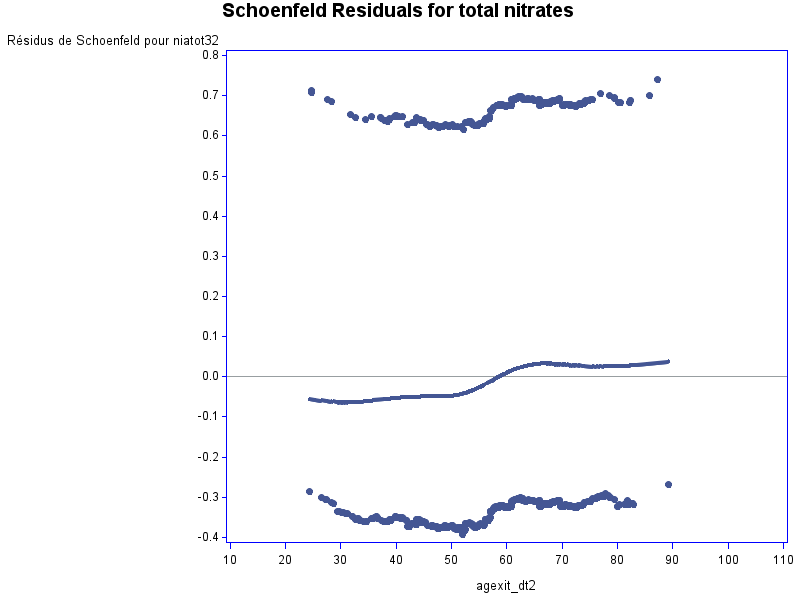


P-value for Pearson correlation between residuals and timescale =0.06

**Schoenfeld Residuals for foods and water-originated nitrates**


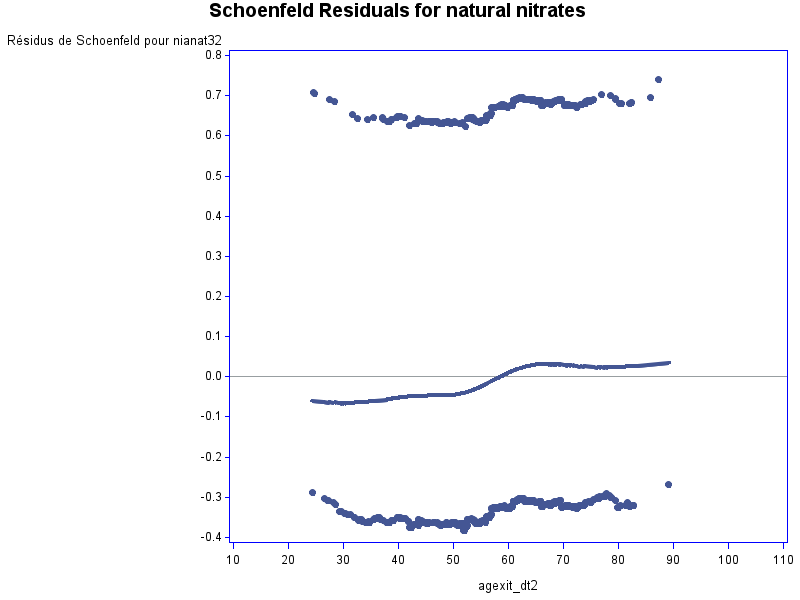


P-value for Pearson correlation between residuals and timescale =0.06

**Schoenfeld Residuals for additives-originated nitrates**


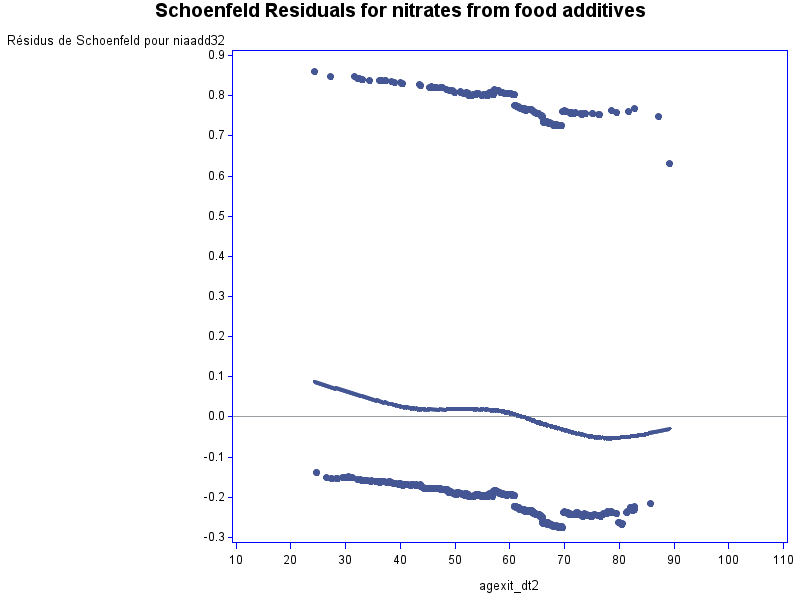


P-value for Pearson correlation between residuals and timescale =0.06

**Table B: Associations between nitrite and nitrate exposures from fruit and vegetables, and red and processed meats, and type 2 diabetes risk, NutriNet-Santé cohort, France, 2009-2021 (n=104,168)**

| **Exposure** |  | **Sex-specific categories of exposure** | | | **P_trend_** |
| --- | --- | --- | --- | --- | --- |
|  |  | **1** | **2** | **3** |  |
| Nitrites from fruits and vegetables | N cases / person-years | 215/213120 | 354/245850 | 400/251152 | 0.4 |
|  | HR (95% CI) | 1 | 1.04 (0.86-1.25) | 1.10 (0.88-1.37) |  |
| Nitrites from red and processed meat | N cases / person-years | 187/226618 | 329/250076 | 453/233429 | 0.01 |
|  | HR (95% CI) | 1 | 1.21 (1.01-1.46) | 1.30 (1.07-1.58) |  |
| Nitrates from fruits and vegetables | N cases / person-years | 245/216952 | 319/248276 | 405/244894 | 0.5 |
|  | HR (95% CI) | 1 | 0.92 (0.77-1.11) | 0.92 (0.74-1.14) |  |
| Nitrates from red and processed meat | N cases / person-years | 198/227554 | 329/247644 | 442/234923 | 0.03 |
|  | HR (95% CI) | 1 | 1.19 (0.99-1.43) | 1.34 (1.10-1.63) |  |

HR: hazard ratio; 95% CI: 95% confidence interval

Multivariable Cox proportional hazard model were adjusted for: age (time scale), sex, energy intake without alcohol (kcal/d, continuous), alcohol (including restricted cubic splines to account for non-linearity), sodium, natural sugars, added sugars, saturated fatty acids, and fiber intakes (g/d, continuous), heme iron intakes (mg/d, continuous), vitamin C intakes (mg/d, continuous), beta-carotene intakes (mg/d, continuous), BMI (kg/m^2^, continuous), physical activity (high, moderate, low, calculated according to IPAQ recommendations), smoking status (never, former, current daily, current occasional smokers), number of pack-years, number of 24 hour dietary records (continuous), family history of diabetes (yes/no), educational level (< High school degree, <2 years after high school, ≥2 years after high school), dietary supplement use (yes/no), artificial sweetener intake (mg/d), proportion of UPF in the diet. All models were mutually adjusted for nitrate/nitrite intakes from the other studied source, and total nitrites/nitrates. For instance, when evaluating nitrites from red and processed meats, we adjusted for nitrites from fruits and vegetables, nitrites from other sources, and total nitrates.

For exposure to nitrites and nitrates and from fruits and vegetables, sex-specific tertiles of exposure were defined. Cut-offs were: 1.2 mg/d and 2.0 mg/d in women and 1.3 mg/d and 2.1 mg/d in men for nitrites, 75.5 mg/d and 130.8 mg/d in women and 76.9 mg/d and 137.2 mg/d in men for nitrates.

For exposure to nitrites and nitrates and from red and processed meat, sex-specific tertiles of exposure were defined. Cut-offs were: 0.7 mg/d and 1.3 mg/d in women and 0.9 mg/d and 1.7 mg/d in men for nitrites, 1.6 mg/d and 3.1 mg/d in women and 2.4 mg/d and 4.3 mg/d in men for nitrates.

**Table C: Sex and antioxidant-stratified associations between exposure to nitrites/nitrates and T2D risk, NutriNet-Santé cohort, 2009-2021 (n=104,168)**

| **Interaction factor** | **Exposure** |  | **Sex-specific categories of exposure** | | | **P_trend_** | **P-interaction** |
| --- | --- | --- | --- | --- | --- | --- | --- |
|  |  |  |  |  |  |  |  |
| **Sex** |  |  | **1** | **2** | **3** |  |  |
| Women | Total nitrites | N cases / person-years | 120/172718 | 199/193922 | 299/192552 |  | 0.11 |
|  |  | HR (95% CI) | 1 | 1.18 (0.92-1.50) | 1.47 (1.14-1.90) | 0.002 |  |
| Men | Total nitrites | N cases / person-years | 90/46533 | 128/53032 | 133/51363 |  |  |
|  |  | HR (95% CI) | 1 | 0.89 (0.67-1.19) | 0.96 (0.70-1.31) | 0.8 |  |
| Women | Foods and water-originated nitrites | N cases / person-years | 117/172321 | 210/195507 | 291/191363 |  | 0.11 |
|  |  | HR (95% CI) | 1 | 1.22 (0.96-1.55) | 1.50 (1.15-1.94) | 0.002 |  |
| Men | Foods and water-originated nitrites | N cases / person-years | 86/46118 | 137/53247 | 128/51563 |  |  |
|  |  | HR (95% CI) | 1 | 0.94 (0.71-1.26) | 0.91 (0.66-1.26) | 0.6 |  |
| Women | Additives-originated nitrites | N cases / person-years | 103/121470 | 226/225573 | 289/212150 |  | 0.3 |
|  |  | HR (95% CI) | 1 | 1.11 (0.86-1.43) | 1.36 (1.06-1.75) | 0.006 |  |
| Men | Additives-originated nitrites | N cases / person-years | 51/31727 | 141/61796 | 159/57405 |  |  |
|  |  | HR (95% CI) | 1 | 1.44 (1.01-2.06) | 1.65 (1.15-2.36) | 0.009 |  |
| Women | Total nitrates | N cases / person-years | 146/170956 | 206/195761 | 270/192475 |  | 0.5 |
|  |  | HR (95% CI) | 1 | 0.97 (0.77-1.22) | 1.04 (0.81-1.33) | 0.7 |  |
| Men | Total nitrates | N cases / person-years | 92/45471 | 120/52997 | 139/52460 |  |  |
|  |  | HR (95% CI) | 1 | 0.88 (0.66-1.18) | 0.82 (0.60-1.13) | 0.2 |  |
| Women | Foods and water-originated nitrates | N cases / person-years | 146/171007 | 202/195690 | 270/192495 |  | 0.5 |
|  |  | HR (95% CI) | 1 | 1.00 (0.80-1.26) | 1.06 (0.83-1.36) | 0.6 |  |
| Men | Foods and water-originated nitrates | N cases / person-years | 93/45482 | 119/52984 | 139/52463 |  |  |
|  |  | HR (95% CI) | 1 | 0.87 (0.66-1.16) | 0.82 (0.60-1.13) | 0.2 |  |
| Women | Additives-originated nitrates | N cases / person-years | 389/366707 | 126/104724 | 103/87760 |  | 0.9 |
|  |  | HR (95% CI) | 1 | 0.94 (0.76-1.16) | 0.97 (0.77-1.22) | 0.7 |  |
| Men | Additives-originated nitrates | N cases / person-years | 191/87756 | 84/34156 | 76/29016 |  |  |
|  |  | HR (95% CI) | 1 | 0.93 (0.70-1.22) | 0.98 (0.73-1.31) | 0.8 |  |
| **Vitamin C** |  |  |  |  |  |  |  |
| ≥ median | Total nitrites | N cases / person-years | 72/84884 | 163/133472 | 284/152416 |  | 0.8 |
|  |  | HR (95% CI) | 1 | 1.00 (0.75-1.33) | 1.30 (0.97-1.75) | 0.02 |  |
| < median | Total nitrites | N cases / person-years | 138/134367 | 164/113482 | 148/91499 |  |  |
|  |  | HR (95% CI) | 1 | 1.03 (0.81-1.32) | 1.08 (0.82-1.42) | 0.6 |  |
| ≥ median | Foods and water-originated nitrites | N cases / person-years | 71/82436 | 169/134130 | 279/154206 |  | 0.7 |
|  |  | HR (95% CI) | 1 | 0.98 (0.74-1.31) | 1.21 (0.90-1.63) | 0.09 |  |
| < median | Foods and water-originated nitrites | N cases / person-years | 132/136003 | 178/114625 | 140/88720 |  |  |
|  |  | HR (95% CI) | 1 | 1.15 (0.90-1.47) | 1.13 (0.86-1.50) | 0.4 |  |
| ≥ median | Additives-originated nitrites | N cases / person-years | 72/81884 | 199/153421 | 248/135468 |  | 0.8 |
|  |  | HR (95% CI) | 1 | 1.32 (1.00-1.75) | 1.72 (1.30-2.28) | <.0001 |  |
| < median | Additives-originated nitrites | N cases / person-years | 82/71313 | 168/133948 | 200/134087 |  |  |
|  |  | HR (95% CI) | 1 | 1.02 (0.76-1.37) | 1.10 (0.82-1.47) | 0.5 |  |
| ≥ median | Total nitrates | N cases / person-years | 77/76573 | 169/132609 | 273/161590 |  | 0.8 |
|  |  | HR (95% CI) | 1 | 0.93 (0.70-1.23) | 0.94 (0.70-1.25) | 0.7 |  |
| < median | Total nitrates | N cases / person-years | 161/139853 | 153/116149 | 136/83345 |  |  |
|  |  | HR (95% CI) | 1 | 0.94 (0.74-1.19) | 0.97 (0.73-1.28) | 0.8 |  |
| ≥ median | Foods and water-originated nitrates | N cases / person-years | 77/76666 | 169/132494 | 273/161612 |  | 0.7 |
|  |  | HR (95% CI) | 1 | 0.93 (0.70-1.23) | 0.93 (0.70-1.25) | 0.7 |  |
| < median | Foods and water-originated nitrates | N cases / person-years | 162/139822 | 152/116180 | 136/83345 |  |  |
|  |  | HR (95% CI) | 1 | 0.94 (0.74-1.20) | 0.98 (0.74-1.29) | 0.8 |  |
| ≥ median | Additives-originated nitrates | N cases / person-years | 312/235037 | 112/74971 | 95/60764 |  | 0.3 |
|  |  | HR (95% CI) | 1 | 0.92 (0.73-1.15) | 0.96 (0.75-1.23) | 0.6 |  |
| < median | Additives-originated nitrates | N cases / person-years | 268/219426 | 98/63909 | 84/56012 |  |  |
|  |  | HR (95% CI) | 1 | 1.01 (0.79-1.29) | 1.01 (0.78-1.32) | 0.9 |  |
| **Vitamin A** |  |  |  |  |  |  |  |
| ≥ median | Total nitrites | N cases / person-years | 94/79999 | 211/137796 | 326/157358 |  | 0.8 |
|  |  | HR (95% CI) | 1 | 0.95 (0.74-1.22) | 1.23 (0.95-1.60) | 0.03 |  |
| < median | Total nitrites | N cases / person-years | 116/139253 | 116/109158 | 106/86556 |  |  |
|  |  | HR (95% CI) | 1 | 1.11 (0.84-1.47) | 1.24 (0.91-1.69) | 0.2 |  |
| ≥ median | Foods and water-originated nitrites | N cases / person-years | 93/78003 | 217/138349 | 321/158800 |  | 0.6 |
|  |  | HR (95% CI) | 1 | 0.94 (0.73-1.21) | 1.18 (0.91-1.54) | 0.07 |  |
| < median | Foods and water-originated nitrites | N cases / person-years | 110/140436 | 130/110405 | 98/84126 |  |  |
|  |  | HR (95% CI) | 1 | 1.28 (0.97-1.69) | 1.27 (0.92-1.75) | 0.1 |  |
| ≥ median | Additives-originated nitrites | N cases / person-years | 89/78715 | 239/156687 | 303/139750 |  | 0.2 |
|  |  | HR (95% CI) | 1 | 1.31 (1.00-1.71) | 1.67 (1.28-2.18) | <0.001 |  |
| < median | Additives-originated nitrites | N cases / person-years | 65/74481 | 128/130682 | 145/129804 |  |  |
|  |  | HR (95% CI) | 1 | 1.11 (0.80-1.54) | 1.25 (0.90-1.73) | 0.2 |  |
| ≥ median | Total nitrates | N cases / person-years | 101/61470 | 205/132762 | 325/180921 |  | 0.2 |
|  |  | HR (95% CI) | 1 | 0.86 (0.67-1.10) | 0.79 (0.61-1.03) | 0.09 |  |
| < median | Total nitrates | N cases / person-years | 137/154957 | 117115996 | 84/64014 |  |  |
|  |  | HR (95% CI) | 1 | 0.94 (0.72-1.24) | 1.09 (0.79-1.52) | 0.6 |  |
| ≥ median | Foods and water-originated nitrates | N cases / person-years | 102/61519 | 204/132675 | 325/180959 |  | 0.2 |
|  |  | HR (95% CI) | 1 | 0.85 (0.67-1.09) | 0.79 (0.61-1.02) | 0.09 |  |
| < median | Foods and water-originated nitrates | N cases / person-years | 137/154969 | 117/116000 | 84/63998 |  |  |
|  |  | HR (95% CI) | 1 | 0.96 (0.73-1.26) | 1.11 (0.80-1.54) | 0.6 |  |
| ≥ median | Additives-originated nitrates | N cases / person-years | 363/231933 | 143/79833 | 125/63386 |  | 0.1 |
|  |  | HR (95% CI) | 1 | 0.98 (0.80-1.20) | 1.02 (0.82-1.27) | 0.9 |  |
| < median | Additives-originated nitrates | N cases / person-years | 217/222531 | 67/59046 | 54/53390 |  |  |
|  |  | HR (95% CI) | 1 | 0.90 (0.67-1.22) | 0.96 (0.70-1.31) | 0.7 |  |
| **Vitamin E** |  |  |  |  |  |  |  |
| ≥ median | Total nitrites | N cases / person-years | 93/88759 | 188/130438 | 300/147202 |  | 0.1 |
|  |  | HR (95% CI) | 1 | 0.99 (0.77-1.29) | 1.31 (1.00-1.72) | 0.01 |  |
| < median | Total nitrites | N cases / person-years | 117/130492 | 139/116516 | 132/96712 |  |  |
|  |  | HR (95% CI) | 1 | 1.12 (0.85-1.46) | 1.22 (0.91-1.65) | 0.2 |  |
| ≥ median | Foods and water-originated nitrites | N cases / person-years | 91/87383 | 198/131052 | 292/147964 |  | 0.05 |
|  |  | HR (95% CI) | 1 | 1.02 (0.79-1.33) | 1.27 (0.96-1.67) | 0.04 |  |
| < median | Foods and water-originated nitrites | N cases / person-years | 112/131056 | 149/117702 | 127/94963 |  |  |
|  |  | HR (95% CI) | 1 | 1.21 (0.93-1.58) | 1.28 (0.94-1.73) | 0.1 |  |
| ≥ median | Additives-originated nitrites | N cases / person-years | 88/83301 | 216/145289 | 277/137809 |  | 0.5 |
|  |  | HR (95% CI) | 1 | 1.34 (1.02-1.76) | 1.61 (1.23-2.12) | <0.001 |  |
| < median | Additives-originated nitrites | N cases / person-years | 66/69896 | 151/142079 | 171/131745 |  |  |
|  |  | HR (95% CI) | 1 | 1.14 (0.83-1.56) | 1.38 (1.00-1.91) | 0.03 |  |
| ≥ median | Total nitrates | N cases / person-years | 106/80637 | 188/130619 | 287/155142 |  | 0.09 |
|  |  | HR (95% CI) | 1 | 0.87 (0.68-1.12) | 0.82 (0.63-1.07) | 0.2 |  |
| < median | Total nitrates | N cases / person-years | 132/135789 | 134/118139 | 122/89793 |  |  |
|  |  | HR (95% CI) | 1 | 1.06 (0.82-1.38) | 1.18 (0.87-1.60) | 0.3 |  |
| ≥ median | Foods and water-originated nitrates | N cases / person-years | 107/80661 | 187/130561 | 287/155176 |  | 0.07 |
|  |  | HR (95% CI) | 1 | 0.86 (0.67-1.11) | 0.82 (0.63-1.06) | 0.2 |  |
| < median | Foods and water-originated nitrates | N cases / person-years | 132/135827 | 134/118113 | 122/89781 |  |  |
|  |  | HR (95% CI) | 1 | 1.08 (0.83-1.39) | 1.18 (0.87-1.60) | 0.3 |  |
| ≥ median | Additives-originated nitrates | N cases / person-years | 341/231218 | 123/73399 | 117/61782 |  | 0.5 |
|  |  | HR (95% CI) | 1 | 0.96 (0.77-1.20) | 1.07 (0.85-1.35) | 0.6 |  |
| < median | Additives-originated nitrates | N cases / person-years | 239/223246 | 87/65481 | 62/54994 |  |  |
|  |  | HR (95% CI) | 1 | 0.93 (0.71-1.21) | 0.89 (0.66-1.20) | 0.4 |  |

Multivariable Cox proportional hazard model adjusted for age (time scale), sex, energy intake without alcohol (kcal/d, continuous), alcohol (including restricted cubic splines to account for non-linearity), sodium, natural sugars, added sugars, saturated fatty acids, and fiber intakes (g/d, continuous), heme iron intakes (mg/d, continuous), vitamin C intakes (mg/d, continuous), beta-carotene intakes (mg/d, continuous), BMI (kg/m^2^, continuous), physical activity (high, moderate, low, calculated according to IPAQ recommendations), smoking status (never, former, current daily, current occasional smokers), number of pack-years, number of 24 hour dietary records (continuous), family history of diabetes (yes/no), educational level (< High school degree, <2 years after high school, ≥2 years after high school), dietary supplement use (yes/no), artificial sweetener intake (mg/d), proportion of UPF in the diet. All models were mutually adjusted for nitrate/nitrite intakes other than the specific one studied. For instance, when evaluating additives-originated nitrites, we adjusted for foods and water-originated nitrites and for total nitrates. For exposure to total nitrites and nitrates and foods and water-originated nitrites and nitrates, sex-specific tertiles of exposure were defined. Cut-offs were: 4.03mg/d and 5.55mg/d in women and 5.18mg/d and 7.44mg/d in men for total nitrites, 150.09mg/d and 233.89mg/d in women and 162.11mg/d and 251.59mg/d in men for total nitrates, 3.83mg/d and 5.29mg/d in women and 4.92mg/d and 7.07mg/d in men for foods and water-originated nitrites, 149.91mg/d and 233.75mg/d in women and 161.94mg/d and 251.32mg/d in men for foods and water-originated nitrates. For additives-originated nitrites and nitrates, 3 categories of exposure were defined: non-exposed, lower exposure and higher exposure (separated by sex-specific median among exposed participants). Cut-offs were: 0.19mg/d in women and 0.25mg/d in men for additives-originated nitrites, 0.36mg/d in women and 0.46mg/d in men for additives originated nitrates. Interaction tests were performed using a sex-specific binary variable for antioxidants, the cut-off being the sex-specific median. Sex specific cut-offs for antioxidants were defined as follows: 1,014.7mg/d in men and 886.8mg/d in women for vitamin A, 112.9mg/d in men and 103.5mg/d in women for vitamin C, and 12.3mg/d in men and 10.6mg/d in women for vitamin E.

| **Table D: Associations between nitrite and nitrate exposures and type 2 diabetes risk - sensitivity analyses, NutriNet-Santé cohort, France, 2009-2021 (n=104,168)** | | | | | | |
| --- | --- | --- | --- | --- | --- | --- |
| **Model** | **Exposure** |  | **Sex-specific categories of exposure** | | | **P_trend_** |
|  |  |  | **1** | **2** | **3** |  |
| **1** | **Total nitrites** | N cases / Person-years | 148/219178 | 229/246852 | 309/243787 |  |
|  |  | HR (95% CI) | 1 | 1.01 (0.81-1.26) | 1.27 (1.00-1.60) | 0.02 |
|  | Foods and water-originated nitrites | N cases / Person-years | 142/218369 | 248/248651 | 296/242798 |  |
|  |  | HR (95% CI) | 1 | 1.09 (0.88-1.36) | 1.25 (0.98-1.58) | 0.05 |
|  | Additives-originated nitrites | N cases / Person-years | 104/153143 | 255/287242 | 327/269433 |  |
|  |  | HR (95% CI) | 1 | 1.27 (0.99-1.64) | 1.65 (1.28-2.12) | <0.001 |
|  | **Total nitrates** | N cases / Person-years | 167/216353 | 231/248658 | 288/244806 |  |
|  |  | HR (95% CI) | 1 | 0.98 (0.79-1.21) | 1.00 (0.79-1.26) | 0.9 |
|  | Foods and water-originated nitrates | N cases / Person-years | 168/216415 | 230/248574 | 288/244828 |  |
|  |  | HR (95% CI) | 1 | 0.98 (0.79-1.26) | 1.00 (0.79-1.26) | 0.9 |
|  | Additives-originated nitrates | N cases / Person-years | 413/454284 | 145/138809 | 128/116724 |  |
|  |  | HR (95% CI) | 1 | 0.84 (0.69-1.03) | 0.94 (0.76-1.16) | 0.3 |
| **2** | **Total nitrites** | N cases / Person-years | 210/219252 | 327/246955 | 432/243915 |  |
|  |  | HR (95% CI) | 1 | 1.02 (0.85-1.23) | 1.22 (1.00-1.48) | 0.02 |
|  | Foods and water-originated nitrites | N cases / Person-years | 203/218439 | 347/248755 | 419/242927 |  |
|  |  | HR (95% CI) | 1 | 1.08 (0.90-1.29) | 1.21 (0.99-1.48) | 0.04 |
|  | Additives-originated nitrites | N cases / Person-years | 154/153197 | 367/287369 | 448/269555 |  |
|  |  | HR (95% CI) | 1 | 1.24 (1.00-1.52) | 1.50 (1.22-1.85) | <0.001 |
|  | **Total nitrates** | N cases / Person-years | 238/216427 | 322/248759 | 409/244936 |  |
|  |  | HR (95% CI) | 1 | 0.96 (0.80-1.15) | 1.03 (0.84-1.26) | 0.7 |
|  | Foods and water-originated nitrates | N cases / Person-years | 239/216489 | 321/248675 | 409/244958 |  |
|  |  | HR (95% CI) | 1 | 0.97 (0.81-1.16) | 1.04 (0.85-1.27) | 0.6 |
|  | Additives-originated nitrates | N cases / Person-years | 580/454464 | 210/138880 | 179/116777 |  |
|  |  | HR (95% CI) | 1 | 0.91 (0.77-1.08) | 0.96 (0.80-1.15) | 0.5 |
| **3** | **Total nitrites** | N cases / Person-years | 210/219252 | 327/246955 | 432/243915 |  |
|  |  | HR (95% CI) | 1 | 1.07 (0.89-1.28) | 1.32 (1.08-1.60) | 0.002 |
|  | Foods and water-originated nitrites | N cases / Person-years | 203/218439 | 347/248755 | 419/242927 |  |
|  |  | HR (95% CI) | 1 | 1.13 (0.94-1.35) | 1.31 (1.07-1.60) | 0.006 |
|  | Additives-originated nitrites | N cases / Person-years | 154/153197 | 367/287369 | 448/269555 |  |
|  |  | HR (95% CI) | 1 | 1.23 (1.00-1.51) | 1.52 (1.24-1.87) | <0.001 |
|  | **Total nitrates** | N cases / Person-years | 238/216427 | 322/248759 | 409/244936 |  |
|  |  | HR (95% CI) | 1 | 0.96 (0.80-1.15) | 0.99 (0.81-1.20) | 0.9 |
|  | Foods and water-originated nitrates | N cases / Person-years | 239/216489 | 321/248675 | 409/244958 |  |
|  |  | HR (95% CI) | 1 | 0.97 (0.81-1.16) | 0.99 (0.82-1.21) | 0.9 |
|  | Additives-originated nitrates | N cases / Person-years | 580/454464 | 210/138880 | 179/116777 |  |
|  |  | HR (95% CI) | 1 | 0.90 (0.76-1.06) | 0.96 (0.80-1.14) | 0.4 |
| **4** | **Total nitrites** | N cases / Person-years | 210/219252 | 327/246955 | 432/243915 |  |
|  |  | HR (95% CI) | 1 | 1.04 (0.86-1.25) | 1.20 (0.99-1.46) | 0.05 |
|  | Foods and water-originated nitrites | N cases / Person-years | 203/218439 | 347/248755 | 419/242927 |  |
|  |  | HR (95% CI) | 1 | 1.09 (0.91-1.31) | 1.20 (0.98-1.46) | 0.07 |
|  | Additives-originated nitrites | N cases / Person-years | 154/153197 | 367/287369 | 448/269555 |  |
|  |  | HR (95% CI) | 1 | 1.17 (0.96-1.44) | 1.40 (1.14-1.72) | <0.001 |
|  | **Total nitrates** | N cases / Person-years | 238/216427 | 322/248759 | 409/244936 |  |
|  |  | HR (95% CI) | 1 | 0.96 (0.80-1.15) | 0.96 (0.79-1.17) | 0.7 |
|  | Foods and water-originated nitrates | N cases / Person-years | 239/216489 | 321/248675 | 409/244958 |  |
|  |  | HR (95% CI) | 1 | 0.96 (0.80-1.15) | 0.97 (0.80-1.18) | 0.8 |
|  | Additives-originated nitrates | N cases / Person-years | 580/454464 | 210/138880 | 179/116777 |  |
|  |  | HR (95% CI) | 1 | 0.94 (0.79-1.11) | 1.00 (0.83-1.20) | 0.8 |
| **5** | **Total nitrites** | N cases / Person-years | 199/158787 | 317/200071 | 425/196984 |  |
|  |  | HR (95% CI) | 1 | 1.05 (0.87-1.27) | 1.29 (1.05-1.57) | 0.007 |
|  | Foods and water-originated nitrites | N cases / Person-years | 191/155675 | 338/203234 | 412/196932 |  |
|  |  | HR (95% CI) | 1 | 1.12 (0.93-1.35) | 1.29 (1.05-1.58) | 0.01 |
|  | Additives-originated nitrites | N cases / Person-years | 144/119430 | 361/234144 | 436/202268 |  |
|  |  | HR (95% CI) | 1 | 1.33 (1.08-1.65) | 1.61 (1.30-1.99) | <0.001 |
|  | **Total nitrates** | N cases / Person-years | 224/143068 | 315/198859 | 402/213915 |  |
|  |  | HR (95% CI) | 1 | 0.96 (0.80-1.16) | 0.95 (0.78-1.16) | 0.6 |
|  | Foods and water-originated nitrates | N cases / Person-years | 225/143114 | 314/198789 | 402/213939 |  |
|  |  | HR (95% CI) | 1 | 0.97 (0.81-1.17) | 0.96 (0.78-1.17) | 0.7 |
|  | Additives-originated nitrates | N cases / Person-years | 561/353295 | 206/113569 | 174/88977 |  |
|  |  | HR (95% CI) | 1 | 0.94 (0.79-1.11) | 0.99 (0.82-1.19) | 0.7 |
| **6** | **Total nitrites** | N cases / Person-years | 205/204041 | 321/237612 | 419/233123 |  |
|  |  | HR (95% CI) | 1 | 1.03 (0.86-1.24) | 1.23 (1.01-1.50) | 0.03 |
|  | Foods and water-originated nitrites | N cases / Person-years | 197/203178 | 341/240002 | 407/231596 |  |
|  |  | HR (95% CI) | 1 | 1.10 (0.91-1.32) | 1.25 (1.02-1.53) | 0.03 |
|  | Additives-originated nitrites | N cases / Person-years | 144/135823 | 357/280217 | 444/258736 |  |
|  |  | HR (95% CI) | 1 | 1.22 (0.99-1.51) | 1.51 (1.22-1.87) | <0.001 |
|  | **Total nitrates** | N cases / Person-years | 232/200883 | 316/239870 | 397/234024 |  |
|  |  | HR (95% CI) | 1 | 0.94 (0.79-1.13) | 0.95 (0.78-1.16) | 0.7 |
|  | Foods and water-originated nitrates | N cases / Person-years | 233/200968 | 315/239761 | 397/234047 |  |
|  |  | HR (95% CI) | 1 | 0.95 (0.79-1.14) | 0.96 (0.79-1.17) | 0.7 |
|  | Additives-originated nitrates | N cases / Person-years | 558/424261 | 209/137930 | 178/112585 |  |
|  |  | HR (95% CI) | 1 | 0.93 (0.78-1.09) | 0.99 (0.82-1.18) | 0.7 |
| **7** | Foods and water-originated nitrites | N cases / Person-years | 203/218439 | 347/248755 | 419/242927 |  |
|  |  | HR (95% CI) | 1 | 1.09 (0.90-1.31) | 1.22 (1.00-1.49) | 0.05 |
|  | Additives-originated nitrites | N cases / Person-years | 154/153197 | 367/287369 | 448/269555 |  |
|  |  | HR (95% CI) | 1 | 1.24 (1.01-1.52) | 1.48 (1.21-1.82) | <0.001 |
|  | Foods and water-originated nitrates | N cases / Person-years | 239/216489 | 321/248675 | 409/244958 |  |
|  |  | HR (95% CI) | 1 | 0.94 (0.78-1.14) | 0.92 (0.70-1.24) | 0.5 |
|  | Additives-originated nitrates | N cases / Person-years | 580/454464 | 210/138880 | 179/116777 |  |
|  |  | HR (95% CI) | 1 | 0.92 (0.78-1.09) | 0.97 (0.81-1.16) | 0.6 |

Multivariable Cox proportional hazard model were adjusted for: age (time scale), sex, energy intake without alcohol (kcal/d, continuous), alcohol (including restricted cubic splines to account for non-linearity), sodium, natural sugars, added sugars, saturated fatty acids, and fiber intakes (g/d, continuous), heme iron intakes (mg/d, continuous), vitamin C intakes (mg/d, continuous), beta-carotene intakes (mg/d, continuous), BMI (kg/m^2^, continuous), physical activity (high, moderate, low, calculated according to IPAQ recommendations), smoking status (never, former, current daily, current occasional smokers), number of pack-years, number of 24 hour dietary records (continuous), family history of diabetes (yes/no), educational level (< High school degree, <2 years after high school, ≥2 years after high school), dietary supplement use (yes/no), artificial sweetener intake (mg/d), proportion of UPF in the diet. All models were mutually adjusted for nitrate/nitrite intakes other than the specific one studied. For instance, when evaluating additives-originated nitrites, we adjusted for foods and water-originated nitrites and for total nitrates.

For exposure to total nitrites and nitrates and foods and water-originated nitrites and nitrates, sex-specific tertiles of exposure were defined. Cut-offs were: 4.03mg/d and 5.55mg/d in women and 5.18mg/d and 7.44mg/d in men for total nitrites, 150.09mg/d and 233.89mg/d in women and 162.11mg/d and 251.59mg/d in men for total nitrates, 3.83mg/d and 5.29mg/d in women and 4.92mg/d and 7.07mg/d in men for foods and water-originated nitrites, 149.91mg/d and 233.75mg/d in women and 161.94mg/d and 251.32mg/d in men for foods and water-originated nitrates.

For additives-originated nitrites and nitrates, 3 categories of exposure were defined: non-exposed, lower exposure and higher exposure (separated by sex-specific median among exposed participants). Cut-offs were: 0.19mg/d in women and 0.25mg/d in men for additives-originated nitrites, 0.36mg/d in women and 0.46mg/d in men for additives originated nitrates.

Model 1: main model after excluding the first 2 years of follow-up

Model 2: main model after adjustment for a Healthy dietary pattern, derived from a principal component analysis (unadjusted for ultra-processed food as a pattern)

Model 3: main model after adjustment for sugar-sweetened beverage (unadjusted for added sugars)

Model 4: main model after adjustment for prevalent hypertension, cardiovascular diseases and hypertriglyceridemia

Model 5: main model after restriction to participants older than 30 years old

Model 6: main model after restriction to participants with at least three 24-hr dietary records

Model 7: main model with mutual adjustments between foods and water-originated nitrites, additives-originated nitrites, foods and water-originated nitrates and additives-originated nitrates

**Table E: Associations between dietary exposure to nitrates with T2D risk, adjusted stratified for mouthwash use, France, 2009-2021 (n=25,328)**

| **Exposure** |  | **Sex-specific categories of exposure** | | | **P_trend_** | **P_interaction_** |
| --- | --- | --- | --- | --- | --- | --- |
|  |  | **1** | **2** | **3** |  |  |
| **In mouthwash users** |  |  |  |  |  |  |
| **Total nitrates** | N cases / Person-years | 50/22472 | 60/32589 | 70/34367 |  | 0.4 |
|  | HR (95% CI) | 1 | 0.80 (0.54-1.20) | 0.77 (0.49-1.21) | 0.3 |  |
| Foods and water-originated nitrates | N cases / Person-years | 50/22459 | 60/32603 | 70/34367 |  | 0.4 |
|  | HR (95% CI) | 1 | 0.95 (0.70-1.28) | 1.06 (0.76-1.48) | 0.8 |  |
| Additives-originated nitrates | N cases / Person-years | 98/51231 | 45/22856 | 37/15341 |  | 0.9 |
|  | HR (95% CI) | 1 | 0.95 (0.65-1.38) | 0.85 (0.55-1.30) | 0.5 |  |
| **In mouthwash non-users** |  |  |  |  |  |  |
| **Total nitrates** | N cases / Person-years | 57/43771 | 110/66598 | 115/68582 |  | 0.4 |
|  | HR (95% CI) | 1 | 1.12 (0.80-1.57) | 0.89 (0.60-1.31) | 0.4 |  |
| Foods and water-originated nitrates | N cases / Person-years | 57/43830 | 110/66518 | 115/68603 |  | 0.4 |
|  | HR (95% CI) | 1 | 0.81 (0.54-1.21) | 0.78 (0.50-1.22) | 0.3 |  |
| Additives-originated nitrates | N cases / Person-years | 162/102656 | 67/46513 | 53/29783 |  | 0.9 |
|  | HR (95% CI) | 1 | 1.14 (0.81-1.59) | 0.90 (0.61-1.33) | 0.5 |  |
| **Adjustment for mouthwash use** |  |  |  |  |  |  |
| **Total nitrates** | N cases / Person-years | 107/66243 | 170/99188 | 185/102949 |  |  |
|  | HR (95% CI) | 1 | 0.99 (0.77-1.28) | 0.85 (0.64-1.14) | 0.2 |  |
| Foods and water-originated nitrates | N cases / Person-years | 107/66289 | 170/99121 | 185/102970 |  |  |
|  | HR (95% CI) | 1 | 1.00 (0.77-1.29) | 0.86 (0.64-1.15) | 0.2 |  |
| Additives-originated nitrates | N cases / Person-years | 260/153887 | 112/69369 | 90/45124 |  |  |
|  | HR (95% CI) | 1 | 0.96 (0.76-1.21) | 1.02 (0.78-1.32) | 0.9 |  |

HR: cause-specific hazard ratio; 95% CI: 95% confidence interval

Multivariable Cox proportional hazard model were adjusted for: age (time scale), sex, energy intake without alcohol (kcal/d, continuous), alcohol (including restricted cubic splines to account for non-linearity), sodium, natural sugars, added sugars, saturated fatty acids, and fiber intakes (g/d, continuous), heme iron intakes (mg/d, continuous), vitamin C intakes (mg/d, continuous), beta-carotene intakes (mg/d, continuous), BMI (kg/m^2^, continuous), physical activity (high, moderate, low, calculated according to IPAQ recommendations), smoking status (never, former, current daily, current occasional smokers), number of pack-years, number of 24 hour dietary records (continuous), family history of diabetes (yes/no), educational level (< High school degree, <2 years after high school, ≥2 years after high school), dietary supplement use (yes/no), artificial sweetener intake (mg/d), proportion of UPF in the diet, and mouthwash use (never, ever). All models were mutually adjusted for nitrate/nitrite intakes other than the specific one studied. For instance, when evaluating additives-originated nitrites, we adjusted for foods and water-originated nitrites and for total nitrates.

For exposure to total nitrates and foods and water-originated nitrates, sex-specific tertiles of exposure were defined. Cut-offs were: 150.09mg/d and 233.89mg/d in women and 162.11mg/d and 251.59mg/d in men for total nitrates, 149.91mg/d and 233.75mg/d in women and 161.94mg/d and 251.32mg/d in men for foods and water-originated nitrates.

For additives-originated nitrites and nitrates, 3 categories of exposure were defined: non-exposed, lower exposure and higher exposure (separated by sex-specific median among exposed participants). Cut-offs were: 0.36mg/d in women and 0.46mg/d in men for additives originated nitrates.

**Table F: Cross-sectional associations between dietary exposure to nitrites and nitrates with metabolic syndrome prevalence, NutriNet-Santé cohort, France, 2009-2021 (n=16,450)**

| **Exposure** |  | **Sex-specific categories of exposure** | | | **P_trend_** |
| --- | --- | --- | --- | --- | --- |
|  |  | **1** | **2** | **3** |  |
| **Total nitrites** | N cases / N total | 382/4269 | 711/6087 | 775/6094 |  |
|  | OR (95% CI) | 1 | 1.31 (1.14-1.51) | 1.35 (1.16-1.58) | <0.001 |
| Foods and water-originated nitrites | N cases / N total | 356/4135 | 744/6256 | 768/6059 |  |
|  | OR (95% CI) | 1 | 1.42 (1.23-1.64) | 1.45 (1.24-1.71) | <0.001 |
| Additives-originated nitrites | N cases / N total | 292/302 | 847/7556 | 729/5872 |  |
|  | OR (95% CI) | 1 | 1.06 (0.91-1.24) | 1.06 (0.90-1.25) | 0.5 |
| **Total nitrates** | N cases / N total | 408/3478 | 646/5899 | 814/7073 |  |
|  | OR (95% CI) | 1 | 1.02 (0.88-1.18) | 1.12 (0.96-1.31) | 0.1 |
| Foods and water-originated nitrates | N cases / N total | 410/3478 | 643/5898 | 815/7074 |  |
|  | OR (95% CI) | 1 | 1.01 (0.87-1.16) | 1.11 (0.95-1.30) | 0.1 |
| Additives-originated nitrates | N cases / N total | 1027/9697 | 504/4186 | 337/2567 |  |
|  | OR (95% CI) | 1 | 1.07 (0.95-1.21) | 1.03 (0.89-1.19) | 0.5 |

OR: odds ratio; 95% CI: 95% confidence interval

Logistic regressions were adjusted for: age (time scale), sex, energy intake without alcohol (kcal/d, continuous), alcohol (including restricted cubic splines to account for non-linearity), sodium, natural sugars, added sugars, saturated fatty acids, and fiber intakes (g/d, continuous), heme iron intakes (mg/d, continuous), vitamin C intakes (mg/d, continuous), beta-carotene intakes (mg/d, continuous), physical activity (high, moderate, low, calculated according to IPAQ recommendations), smoking status (never, former, current daily, current occasional smokers), number of pack-years, number of 24 hour dietary records (continuous), educational level (< High school degree, <2 years after high school, ≥2 years after high school), dietary supplement use (yes/no), artificial sweetener intake (mg/d), proportion of UPF in the diet. All models were mutually adjusted for nitrate/nitrite intakes other than the specific one studied. For instance, when evaluating additives-originated nitrites, we adjusted for foods and water-originated nitrites and for total nitrates.

For exposure to total nitrites and nitrates and foods and water-originated nitrites and nitrates, sex-specific tertiles of exposure were defined. Cut-offs were: 4.03mg/d and 5.55mg/d in women and 5.18mg/d and 7.44mg/d in men for total nitrites, 150.09mg/d and 233.89mg/d in women and 162.11mg/d and 251.59mg/d in men for total nitrates, 3.83mg/d and 5.29mg/d in women and 4.92mg/d and 7.07mg/d in men for foods and water-originated nitrites, 149.91mg/d and 233.75mg/d in women and 161.94mg/d and 251.32mg/d in men for foods and water-originated nitrates.

For additives-originated nitrites and nitrates, 3 categories of exposure were defined: non-exposed, lower exposure and higher exposure (separated by sex-specific median among exposed participants). Cut-offs were: 0.19mg/d in women and 0.25mg/d in men for additives-originated nitrites, 0.36mg/d in women and 0.46mg/d in men for additives originated nitrates.

**Table G: Cause-specific associations between dietary exposure to nitrites and nitrates with mortality risk as a competing risk, NutriNet-Santé cohort, France, 2009-2021 (n=104,168)**

| **Exposure** |  | **Sex-specific categories of exposure** | | | **P_trend_** |
| --- | --- | --- | --- | --- | --- |
|  |  | **1** | **2** | **3** |  |
| **Total nitrites** | N cases / Person-years | 346/219252 | 403/246955 | 351/243915 |  |
|  | HR (95% CI) | 1 | 1.05 (0.90-1.23) | 0.95 (0.80-1.13) | 0.6 |
| Foods and water-originated nitrites | N cases / Person-years | 341/218439 | 403/248755 | 356/242927 |  |
|  | HR (95% CI) | 1 | 1.02 (0.88-1.19) | 0.93 (0.78-1.11) | 0.4 |
| Additives-originated nitrites | N cases / Person-years | 296/153197 | 434/287369 | 370/269555 |  |
|  | HR (95% CI) | 1 | 1.03 (0.88-1.20) | 1.09 (0.92-1.29) | 0.3 |
| Sodium nitrite (e250) | N cases / Person-years | 296/153593 | 435/287578 | 369/268950 |  |
|  | HR (95% CI) | 1 | 1.03 (0.88-1.21) | 1.08 (0.92-1.28) | 0.3 |
| **Total nitrates** | N cases / Person-years | 288/216427 | 379/248759 | 433/244936 |  |
|  | HR (95% CI) | 1 | 1.17 (1.00-1.37) | 1.10 (0.93-1.32) | 0.3 |
| Foods and water-originated nitrates | N cases / Person-years | 289/216489 | 381/248675 | 430/244958 |  |
|  | HR (95% CI) | 1 | 1.17 (1.00-1.37) | 1.09 (0.92-1.30) | 0.4 |
| Additives-originated nitrates | N cases / Person-years | 717/454464 | 187/138880 | 196/116777 |  |
|  | HR (95% CI) | 1 | 1.12 (0.94-1.33) | 1.16 (0.98-1.37) | 0.06 |
| Potassium nitrate (e252) | N cases / Person-years | 718/454500 | 186/138895 | 196/116726 |  |
|  | HR (95% CI) | 1 | 1.11 (0.93-1.32) | 1.16 (0.98-1.37) | 0.06 |

HR: cause-specific hazard ratio; 95% CI: 95% confidence interval

Multivariable Cox proportional hazard model were adjusted for: age (time scale), sex, energy intake without alcohol (kcal/d, continuous), alcohol (including restricted cubic splines to account for non-linearity), sodium, natural sugars, added sugars, saturated fatty acids, and fiber intakes (g/d, continuous), heme iron intakes (mg/d, continuous), vitamin C intakes (mg/d, continuous), beta-carotene intakes (mg/d, continuous), BMI (kg/m^2^, continuous), physical activity (high, moderate, low, calculated according to IPAQ recommendations), smoking status (never, former, current daily, current occasional smokers), number of pack-years, number of 24 hour dietary records (continuous), family history of diabetes (yes/no), educational level (< High school degree, <2 years after high school, ≥2 years after high school), dietary supplement use (yes/no), artificial sweetener intake (mg/d), proportion of UPF in the diet. All models were mutually adjusted for nitrate/nitrite intakes other than the specific one studied. For instance, when evaluating additives-originated nitrites, we adjusted for foods and water-originated nitrites and for total nitrates.

For exposure to total nitrites and nitrates and foods and water-originated nitrites and nitrates, sex-specific tertiles of exposure were defined. Cut-offs were: 4.03mg/d and 5.55mg/d in women and 5.18mg/d and 7.44mg/d in men for total nitrites, 150.09mg/d and 233.89mg/d in women and 162.11mg/d and 251.59mg/d in men for total nitrates, 3.83mg/d and 5.29mg/d in women and 4.92mg/d and 7.07mg/d in men for foods and water-originated nitrites, 149.91mg/d and 233.75mg/d in women and 161.94mg/d and 251.32mg/d in men for foods and water-originated nitrates.

For additives-originated nitrites and nitrates, 3 categories of exposure were defined: non-exposed, lower exposure and higher exposure (separated by sex-specific median among exposed participants). Cut-offs were: 0.19mg/d in women and 0.25mg/d in men for additives-originated nitrites, 0.36mg/d in women and 0.46mg/d in men for additives originated nitrates, 0.19mg/d in women and 0.25mg/d in men for sodium nitrite (e250), 0.36mg/d in women and 0.46mg/d in men for potassium nitrate (e252).

**REFERENCES**

1. Black AE. Critical evaluation of energy intake using the Goldberg cut-off for energy intake:basal metabolic rate. A practical guide to its calculation, use and limitations. IntJObesRelat Metab Disord. 2000 Sep;24(0307-0565 (Print)):1119–30.

2. Black AE. The sensitivity and specificity of the Goldberg cut-off for EI:BMR for identifying diet reports of poor validity. European Journal of Clinical Nutrition. 2000 May;54(5):395–404.

3. Goldberg GR, Black AE, Jebb SA, Cole TJ, Murgatroyd PR, Coward WA, et al. Critical evaluation of energy intake data using fundamental principles of energy physiology: 1. Derivation of cut-off limits to identify under-recording. Eur J Clin Nutr. 1991 Dec;45(12):569–81.

4. Schofield WN. Predicting basal metabolic rate, new standards and review of previous work. HumNutr Clin Nutr. 1985;39 Suppl 1(0263-8290 (Print)):5–41.

5. Anses. Etude Individuelle Nationale des Consommations Alimentaires 3 (INCA 3). 2017.

6. European Commission. Database [Internet]. Food Safety - European Commission. 2016 [cited 2020 Oct 7]. Available from: https://ec.europa.eu/food/safety/food_improvement_agents/additives/database_en

7. Food and Agriculture Organization/World Health Organization (FAO/WHO). Codex General Standard for Food Additives (GSFA, Codex STAN 192-1995) [Internet]. Codex Alimentarius Commission; 2019 [cited 2018 Sep 19]. Available from: http://www.fao.org/fao-who-codexalimentarius/sh-proxy/en/?lnk=1&url=https%253A%252F%252Fworkspace.fao.org%252Fsites%252Fcodex%252FStandards%252FCODEX%2BSTAN%2B192-1995%252FCXS_192e.pdf

8. Haute Autorité de Santé. Actualisation du référentiel de pratiques de l’examen périodique de santé: prévention et dépistage du diabète de type 2 et des maladies liées au diabète [Internet]. 2014 Oct [cited 2022 Nov 9]. Available from: https://www.has-sante.fr/upload/docs/application/pdf/2015-02/7v_referentiel_2clics_diabete_060215.pdf
