# Supplementary material for: Dietary exposure to nitrites and nitrates in association with type 2 diabetes risk: Results from the NutriNet-Santé population-based cohort study
Source: PLoS Med. 2023 Jan 17;20(1):e1004149. doi: 10.1371/journal.pmed.1004149 (PMC9844911; doi:10.1371/journal.pmed.1004149)
Supplement: Attachment — Submitted filename: Joshipura_Dietary_Nitrate_Nitrite_Review.docx [file pmed.1004149.s003.docx]

This is a very large well done prospective study evaluating the association between intake of nitrates, and nitrates and risk of type two diabetes over a seven-year follow up. This is an important and understudied area, and the authors also evaluate nitrates/nitrites from food additives separately, which is an added strength. The major limitation that limits the interpretation and validity of the conclusions is the lack of data on mouthwash use, which may likely be a major confounder leading to the lack of association see between intake of nitrates (which is mostly from vegetables) and diabetes. The study also has a few other limitations as described below.

1. The main concern of the 24 hour diet records is the day to day variability in diet and limitation of diet records to capture long-term intake, which may be partly countered by multiple diet records over time that were averaged. Importantly, in the Author Summary, they imply in line 76 that the semi quantitative food frequency questionnaire used by another study is a limitation, whereas it is really a strength. This should be reworded, and the pros and cons of the dietary assessment methods need to be mentioned in the discussion.
2. The authors have attempted to separate nitrate/nitrite sources. However, they have still combined water contamination, processed meat, seasoning, and intake from vegetables, into a category called “natural sources”. The word “natural” does not seem appropriate for water contamination, processed meat, and seasoning. Importantly, it would add a lot of value to separate these sources and evaluate the individual impacts from vegetables, especially since beetroots and green leafy vegetables (two potentially beneficial vegetables) are the main sources of dietary nitrate.
3. Two validation studies are referenced, but they do not validate the assessment of nitrate/nitrite, which may be much more challenging given the different sources.
4. Natural sources should be defined in the abstract. Line 54 of the abstract should specify the sources evaluated instead of saying “any” source.
5. The authors mention soil contamination in the Author Summary, but do not discuss this anywhere else. If this is important, please elaborate in the manuscript.
6. The water nitrate and nitrite were assessed from the records of tap-water levels based on the participants’ residence location. This is a limitation as people may consume varying amounts of tap-water, and some may spend substantial or most time outside their residence. The nitrate and nitrite in the water in their location may not adequately reflect individual consumption. This limitation needs to be discussed.
7. This is a low-risk group with high socioeconomic status and healthier diets. The related concerns about generalizability need to be directly mentioned.
8. The discussion focuses on nitrites, and does not go into details about the lack of association with nitrates. The literature suggests that beetroot and spinach may have a beneficial role in cardiometabolic conditions, partly due to their high nitrate content. The lack of association with nitrates and the implications for diet should be discussed.
9. Importantly, the potential beneficial role of nitrates may be countered by mouthwash use, which may be high in this high income population. Most over-the-counter mouthwash have antibacterial properties which impact nitrate reduction in the mouth by oral bacteria, and impact nitric oxide metabolism. Recent studies have shown that regular over-the-counter mouthwash use is associated with increased risk of diabetes. Hence, the null results may be partly attributed to mouthwash use. If data is available, the authors should control for mouthwash use, and also presents analyses stratified by mouthwash use. If data is not available to control/stratify on mouthwash use, this should be discussed as a major limitation in the abstract, discussion and conclusion, as this may explain the null association with nitrates.
10. Appendix 7 should show effect estimates, not just p-values. Also, it would be helpful to discuss differences in effect estimates across the antioxidant subgroups, as interaction tests often do not have enough power.
11. Appendix 9 title mentions “Cause-specific…” This should be reworded, as one cannot interpret causality from this study. Also, the manuscript erroneously refers to Appendix 9 as Appendix 8.
