# Supplementary material for: Dietary exposure to nitrites and nitrates in association with type 2 diabetes risk: Results from the NutriNet-Santé population-based cohort study
Source: PLoS Med. 2023 Jan 17;20(1):e1004149. doi: 10.1371/journal.pmed.1004149 (PMC9844911; doi:10.1371/journal.pmed.1004149)
Supplement: Attachment — Submitted filename: response_V1609.docx [file pmed.1004149.s004.docx]

-----------------------------------------------------------

Comments from the Academic Editor:

The authors conducted novel observational analyses. Besides the comments from the other reviewers, the following comments should be accounted for to identify the appropriateness of this manuscript for potential publication.

+++ We thank the Academic Editor for his/her comments. All of them have been addressed below.

The major comments are as follows:

The authors should revise the covariate set to adjust for in their regression analyses. Some concerns are present.

1-1. First, an association of alcohol consumption with T2D is known to be non-linear. The authors should fit restricted cubic spline terms.

+++ We have now fitted restricted cubic spline terms for alcohol consumption in all the models to account for non-linearity. Figures have been modified throughout the text and tables. This modification did not change the main results.

1-2. Second, the authors should control for sugar-sweetened beverage (SSB) consumption. It is unlikely to be a source of nitrates and nitrites, unless some products uniquely contain those as ingredients, so its adjustment should be fine. Similarly or alternatively, the authors should control for added sugars from beverages and added sugars from solid foods (e.g. cakes). Sugars naturally occurring in foods and added sugars may have differential associations with T2D, as implicated in studies on SSB. Moreover, this current study is relevant to food processing or additives, and thus separation between added sugars and natural sugars is critical in covariate adjustment. In general, authors should be equally careful in exposure and covariate treatments.

+++ In the main model, we have now replaced the adjustment for total sugars with two separate variables: natural sugars and added sugars. Besides, we tested further adjustment for SSB (in ml/d) in a sensitivity analysis (Table D in S1-Appendix, model 3). The results remained unchanged.

1-3. Third, when the authors evaluated naturally-occurring nitrites and nitrites from food additives, they should adjust for those mutually. It was great that the authors clarified the application of mutual adjustment between nitrates and nitrites. This care is necessary for the analysis of sources of nitrates and nitrites. The authors should consider a time-to-event regression model to include nitrites from natural sources, nitrites from additives, nitrates from natural sources, and nitrates from additives.

+++ We apologize if our wording was not accurate. This is indeed what has been done: for instance, when evaluating nitrites from food additives, we adjusted for foods and water-originated nitrites and for total nitrates. We have now clarified our adjustment strategy in the methods page 10 and in the footnotes to figure 3.

Additionally, as a sensitivity analysis, we have now built a Cox model including simultaneously the four exposure variables: foods and water-originated nitrites, additives-originates nitrites, foods and water-originated nitrates and additives-originated nitrates. In this mutually adjusted model (model 7), associations were detected for foods and water-originated nitrites (HR_tertile 3 vs.1_= 1.22 (95% CI 1.00-1.49), P_-trend_=0.05), and additives-originated nitrites (HR_higher consumers vs. non-consumers_ = 1.48 (95% CI 1.21-1.82, P_-trend_=<0.001), but not for foods and water-originated or additives-originated nitrates (P_-trend_ >0.5).

This has been added to the Results section page 14 and to Table F in S1-Appendix, Model 7.

1-4. The authors should control for intakes of vitamin C and carotenoids from foods, at least. Those intakes or biomarkers are known to be associated with incident T2D. They are likely confounders influencing the potential effect of fruits and vegetables that provide nitrates or nitrites.

+++ We now adjust all models for intakes of vitamin C and beta-carotene (as total carotenoid intake was not available in our food composition database). This did not modify the findings. As we had already a sensitivity model adjusted for antioxidant intakes (previously model 2 in sensitivity analyses), this model was not deleted.

1-5. The authors should control for supplement use, artificial sweetener intakes, and others which the authors may come up with as possible indicators of health consciousness. In this study, health consciousness remains a source of unmeasured confounders, and the authors must want to minimize the effect as much as possible.

+++ We thank the Academic Editor for this comment. We now adjust our models for dietary supplement use and total artificial sweetener intakes as potential markers of health consciousness, and describe these variables in Table 1. These further adjustments did not substantially modify the results.

2. The authors should evaluate nitrates and nitrites from different food sources in another way additionally. As expected, those chemicals mainly come from meat/processed meat consumption, regardless of how the authors identified or defined natural sources or food additives. In general, the positive associations of meat/processed meat consumption with incident T2D have been well-recognized. If those food groups were major drivers of the observed associations of nitrites, the inference from this study would not impact general dietary recommendations.

To clarify if that is the case, the authors should evaluate, for example, nitrites from fruits and vegetables and nitrites from unprocessed and processed meat products separately, with mutual adjustment.

Of note, if nitrite intake from fruits and vegetables showed positive associations with T2D incidence, that would be a meaningful finding that scientists should get aware of for further research.

The authors may find that nitrite intakes from fruits and vegetables showed no significant associations or negative associations (after adjustment for vitamin C, carotenoids, nitrites from other sources, and nitrates from different sources). This finding would be important to clarify no evidence for harm from nitrites in fruits and vegetables.

Collinearity is likely between nitrites from fruits and vegetables, vitamin C, and carotenoids. The authors should present results for the indication, if any, and state the weaknesses of the evidence for nitrites/nitrates from fruits and vegetables.

+++ We thank the Academic Editor for this suggestion. We have now performed additional analyses by evaluating nitrites and nitrates from fruits and vegetables and those from unprocessed and processed meats, separately, with mutual adjustment. These results are presented in a new table: Table B in S1-Appendix and they are mentioned in the manuscript page 14.

As expected by the Academic Editor, statistically significant associations were observed for nitrites from red and processed meats (HR_tertile 3 vs.1_= 1.30 (95% CI 1.30-1.58), P-trend=0.01) and nitrates from red and processed meats (HR_tertile 3 vs.1_= 1.34 (95% CI 1.10-1.63), P-trend=0.03), but not for nitrites from fruits and vegetables (HR_tertile 3 vs.1_= 1.10 (95% CI 0.88-1.37), P-trend=0.4) or nitrates from fruits and vegetables (HR_tertile 3 vs.1_= 0.92 (95% CI 0.74-1.14), P-trend=0.5).

Pearson correlation coefficients were 0.41 for vitamin C, and 0.50 for beta-carotene with nitrites from fruits and vegetables, and 0.31 for vitamin C, and 0.58 for beta-carotene with nitrates from fruits and vegetables. This has been added in the discussion page 16.

It is indeed challenging to disentangle the individual contributions of different compounds of a food group (i.e., red and processed meats or fruits and vegetables) in modulating T2D risk in observational studies: as regards red and processed meats, our models were adjusted for heme iron, saturated fatty acids, and sodium intakes, and the associations with nitrites and nitrates coming from these sources remained significant. Even though these results would not directly impact the existing dietary guidelines (recommending to reduce the consumption of red and processed meats), they provide new evidence in the context of current discussions regarding the need for a further regulation of nitrites and nitrates use, from both food additives and soil and water contamination. On the other hand, the absence of an association in our study between nitrites and nitrates from fruits and vegetables and T2D risk is in line with dietary guidelines encouraging their consumption, even though the evidence from our study is relatively weak given the moderate-to-strong correlations between nitrites and nitrates from fruits and vegetables with their other components.

We have now discussed this point in the discussion, that now reads:

*‘Green leafy vegetables are the major source of dietary nitrates. Even though their role in reducing T2D risk has been suggested* [1,2]*, the most recent meta-analyses* [3–5] *have reported low to very low quality of evidence for the association of incidence of cruciferous and green leafy vegetables with T2D risk. This is in line with our findings regarding the lack of association between foods-originated nitrates with T2D risk, or fruits and vegetables-originated nitrites and nitrates with T2D risk. In any case, even though the evidence from our study for these specific analyses might be relatively weak given the 1) moderate-to-strong correlations between nitrites and nitrates from fruits and vegetables with their other components (e.g., vitamin C [Pearson correlation coefficients = 0.41 for nitrites and 0.31 for nitrates], beta-carotene [Pearson correlation coefficients = 0.50 for nitrites and 0.58 for nitrates], dietary fibers [Pearson correlation coefficients = 0.63 for nitrites and 0.57 for nitrates]), 2) the possible lack of statistical power, and 3) potential residual confounding or classification biases, vegetables remain important sources of fibers and antioxidants, and are beneficial components of a healthy diet playing an important role in the prevention of chronic diseases such as cardiovascular diseases and some cancers. Previously published meta-analyses* [3,6] *reported high evidence levels for the role of processed meat, an important contributor to intakes of nitrites as food additives, as a risk factor for T2D risk. Although it is not possible to fully disentangle the effects of each processed meat constituent, the fact that our models were adjusted for others components that could play a role in T2D aetiology (e.g. pro-oxidant heme iron, saturated fatty acids, sodium) suggests that nitrites contained in processed meat might causally contribute to processed meat - T2D associations, which is in line with dietary guidelines recommending to reduce red and processed meats consumption.’*

3.

In the presentation of hazard ratios, the authors should replace the numbers of participants (denominator) with person-time values. The authors evaluated hazard ratios which have close relevance to incidence (N events/ person-time) more than risk (N events/ specific time length). Thus, the authors should show person-time values to allow interpretations of incidence measures.

+++ We have now replaced the number of participants with person-years throughout the manuscript and supplementary materials.

4.

The authors should present the results from cubic spline analyses and food sources of nitrites and nitrates in the main manuscript, not in the supplementary materials. Those are critical information to present.

+++ We have now moved results from cubic splines and food sources of nitrites and nitrates to the main manuscript (figures 1, 2 and 4).

Minor comments:

Table 1. The authors can take out the column of P-value and mention that all trends were significant in the footnote.

+++ This modification has been done accordingly

Requests from the editors:

Please revise your title according to PLOS Medicine's style. Your title must be nondeclarative and not a question. It should begin with main concept if possible. "Effect of" should be used only if causality can be inferred, i.e., for an RCT. Please place the study design ("A randomized controlled trial," "A retrospective study," "A modelling study," etc.) in the subtitle (ie, after a colon).

+++ The title has been revised as: “Dietary exposure to nitrites and nitrates in association with type-2 diabetes risk: Results from the NutriNet-Santé population-based cohort study ” (page 1)

Abstract Methods and Findings:

* Please include the length of follow up (information found at line 260).

+++ Length of follow-up has been added to the abstract.

* In the last sentence of the Abstract Methods and Findings section, please describe the main limitation(s) of the study's methodology.

+++ The following sentence has been added to the abstract:

*‘No causal link can be established from this observational study. Main limitations include potential selection bias linked to the healthier behaviours of the cohort’s participants compared to the general population, potential residual confounding linked to the observational design, as well as a self-reported, yet cross-checked, case ascertainment.’*

Lines 83-86: Please confirm the correct HR is presented in this bullet point as the abstract suggests it ought to be ‘1.60 (95% CI 1.29-54 1.98), P¬trend<0.001’ for e250 specifically.

+++ This has been corrected.

Citations should be placed within square brackets.

+++ Done throughout the manuscript

Line 273: Please round p values to 3 decimal places (state p<0.001).

+++ This has been corrected throughout the manuscript and the supplementary material.

Table 1 and Figure 1: Footnote ‘*’ is missing the corresponding flag in the table/figure.

+++ This has been corrected.

For the adjusted analyses in Figure 1, please also present the crude (unadjusted) analyses in the Supporting Information and cite them in your results.

+++ Age and sex adjusted analyses were now presented in Table A in S1-Appendix and cited in the results.

Figure 1 legend should refer to Appendix 9 rather than 8.

+++ This has been corrected.

Discussion: Please ensure any claims of primacy are tempered by the addition of ‘to our knowledge’, or similar.

+++ All claims of primacy have been tempered by adding ‘to our knowledge’.

Pages 17-19: Please remove the ‘Funding’, ‘Contributorship statement and guarantor’, ‘Transparency statement’, and ‘IARC disclaimer’ subsections – this information will be captured via the submission form and presented as metadata in the event of publication. The ‘Patient involvement statement’ can be relocated to the Methods section.

+++ These sections have been removed.

Appendix 6: Please enlarge the labels for your axes and your title font. Please also refer to p<0.001 rather than P=0.0009.

+++ These edits have been performed.

Please ensure that the study is reported according to the STROBE guideline, and include the completed STROBE checklist as Supporting Information. Please add the following statement, or similar, to the Methods: "This study is reported as per the Strengthening the Reporting of Observational Studies in Epidemiology (STROBE) guideline (S1 Checklist)."

The STROBE guideline can be found here: <http://www.equator-network.org/reporting-guidelines/strobe/>

When completing the checklist, please use section and paragraph numbers, rather than page numbers.

+++ We ensured that the study was reported according to the STROBE-nut guideline, included the completed STROBE-nut checklist as Supporting Information and added a statement to the Methods section, page 11: *“**All methods have been described in line with the Strengthening the Reporting of Observational Studies in nutritional Epidemiology guidelines (S1 STROBE-nut Checklist)”.*

Did your study have a prospective protocol or analysis plan? Please state this (either way) early in the Methods section.

a) If a prospective analysis plan (from your funding proposal, IRB or other ethics committee submission, study protocol, or other planning document written before analyzing the data) was used in designing the study, please include the relevant prospectively written document with your revised manuscript as a Supporting Information file to be published alongside your study, and cite it in the Methods section. A legend for this file should be included at the end of your manuscript.

b) If no such document exists, please make sure that the Methods section transparently describes when analyses were planned, and when/why any data-driven changes to analyses took place.

c) In either case, changes in the analysis-- including those made in response to peer review comments-- should be identified as such in the Methods section of the paper, with rationale.

+++ Details have been added in the Methods section, page 7 as follows: *“The NutriNet-Santé study has been developed to investigate the relationships between multiple dietary exposures and the incidence of chronic diseases, such as cancer, using a prospective approach. The general protocol of the cohort, written in 2008 before the beginning of the study, is available online* [7]*. Regarding food additives specifically, the present work is part of a series of pre-specified analyses that are included in a project funded by the European Research Council (ERC, https://erc.europa.eu/news-events/magazine/erc-2019-consolidator-grants-examples#ADDITIVES).”*

And page 11 as follows:

*‘Multi-adjusted Cox models for several confounders were pre-specified. The main analyses added following the review process were as follows: further adjustments (for dietary supplement use, artificial sweeteners intake, vitamin C and beta-carotene intakes, added sugar intake, restricted cubic splines for alcohol consumption, and dietary pattern using ultra-processed food consumption in the main model, prevalent hypertension, cardiovascular diseases and hypertriglyceridemia, and consumption of sugar sweetened beverages in sensitivity analyses), analyses by food sources (fruits and vegetables, red and processed meats), the mutually adjusted model, adjustment and stratification for mouthwash use in sensitivity analyses, and cross-sectional analyses with metabolic syndrome in exploratory analyses.’*

To an extent that is reasonably practical, please avoid textual overlap with your prior publication (Chazelas et al., Int J Epidemiol, 2022; <https://academic.oup.com/ije/advance-article/doi/10.1093/ije/dyac046/6550543>).

+++ We have made some reformulations throughout the text to limit overlap with this prior publication in the methods, whenever possible. According to our anti-plagiarism software (Compilatio), we have now reached only 3% textual overlap with our prior publication, only in the generic methods section.

Comments from the reviewers:

Reviewer #1: This is an interesting manuscript analyzing the prospective association between dietary nitrites and nitrates from natural sources and food additives and type 2 diabetes in the NutriNet-Sante cohort with a huge sample size. Overall the manuscript is clear and well written, delivering important public health relevant message about the health effect of dietary nitrites and nitrates especially from the food additives. This reviewer has several comments to help improve the manuscript:

+++ We thank Reviewer #1 for his/her positive general comment and for the suggestions to improve the manuscript. All have been accounted for below.

1)	Exposure assessment. The reviewer appreciates their effort to validate the web-based 24h dietary records. It seems that the specifical validation for the nitrites and nitrates is lacking? Is there any way to validate these measurements, say, against biomarkers? The results were based on "at least 2 valid 24 dietary records during their first t years of follow-up", so it might be rather random.

+++ Indeed, prior to the launching of the NutriNet-Santé cohort in 2008-2009, two studies have been performed to validate the web-based dietary records against biomarkers: one against plasma biomarkers [8] suggested that this tool provides reasonable estimates of true intake of fruits, vegetables, fish, beta carotene, vitamin C, and n-3 fatty acids. The other one against urinary biomarkers [9] showed that these web-based dietary records appear to be valid for estimating usual intakes of proteins and potassium. Besides, the self-administered web-based dietary record tool has also been validated against an interview by a trained dietician showing high correlations for all food groups, energy, macro and micro-nutrients [10]. This has been clarified in the Methods page 7.

The accuracy of the qualitative and quantitative food additives exposure assessment of the NutriNet-Santé cohort represents an important strength of the study, thanks to detailed and repeated 24h-dietary records, links to multiple food composition databases (OQALI [11], Open Food Facts [12], GNPD [13], EFSA [14], and GSFA [15]), ad-hoc laboratory assays, and dynamic matching to account for reformulations of industrial food items over time [16]. We fully agree with Reviewer #1 that validation of additive exposure versus plasmatic or urinary biomarkers would be ideal, however, it is not possible for many food additives, among which nitrites and nitrates.

Indeed, specific validation of nitrites/nitrates intakes versus biomarkers has been envisioned at the beginning of the ADDITIVES ERC Program, but after careful examination of biomarker literature and scientific discussions among partners, it has been judged not feasible. Indeed, as for many food additives, it was not possible to identify markers in blood or urine that would be specific enough to reflect exogenous dietary exposure and not endogenous metabolism. More specifically, for instance, plasma nitrate would not only be a suitable marker for dietary sources, but would also reflect nitrate derived from various individual (patho‐) physiological pathways, as well as the individual capacity to eliminate nitrate [17–19]. Similarly for urinary nitrites, in addition to the fact that urinary tract infections are an important driver of urine nitrites levels [20], therefore providing biased estimates of dietary exposure.

We have added these elements to the discussion page 20:

*‘**Lastly, exposure to nitrates and nitrates has not been validated with blood or urine assays due to lack of biomarkers that would be specific enough to reflect exogenous dietary exposure and not endogenous metabolism. For instance, plasma nitrate would not only be a suitable marker for dietary sources, but would also reflect nitrate derived from various individual (patho‐) physiological pathways, as well as the individual capacity to eliminate nitrate* [17–19]*. Similarly for urinary nitrites, in addition to the fact that urinary tract infections are an important driver of urine nitrites levels* [20]*, therefore providing biased estimates of dietary exposure.’*

We have chosen to keep participants having at least 2 valid 24h dietary records in the main analysis to ensure a good compromise between data precision and statistical power / limitation of selection bias. However, to comply with this comment, we have now performed a sensitivity analysis in participants having at least three 24h dietary records: results were unchanged. This has been added to the methods page 11, the results page 14 and Table D in S1-Appendix, model 6.

2)	Outcome ascertainment. It seems that this was based on the self-reported data. It is not clear about the "biological data". Do they provide blood sample for the measurement of glucose or HbA1c? Would underreporting at baseline be possible?

+++ T2D case ascertainment was based on self-report, but was confirmed using a multi-source strategy. Reporting both incident T2D and the use of T2D medication via the baseline and health follow-up questionnaire (administered every 3 months) or the dedicated health events platform, was mandatory for the case to be considered as a confirmed T2D case. The questions were: “Have you been diagnosed with T2D (if yes, indicate the date of diagnosis)” and “Are you treated for T2D ?”. Participants are asked to record all currently taken medications (T2D medications and others). A search engine with embedded exhaustive Vidal® drug database is used to facilitate medication data entry for the participants. ATC codes considered for T2D medication were A10AB01, A10AB03, A10AB04, A10AB05, A10AB06, A10AC01, A10AC03, A10AC04, A10AD01, A10AD03, A10AD04, A10AD05, A10AE01, A10AE02, A10AE03, A10AE04, A10AE05, A10AE30, A10BA02, A10BB01, A10BB03, A10BB04, A10BB06, A10BB07, A10BB09, A10BB12, A10BD02, A10BD03, A10BD05, A10BD07, A10BD08, A10BD10, A10BD15, A10BD16, A10BF01, A10BF02, A10BG02, A10BG03, A10BH01, A10BH02, A10BH03, A10BX02, A10BX04, A10BX07, A10BX09, A10BX10, A10BX11, A10BX12.

Following a T2D diagnosis and/or medication declaration, two additional sources of information were considered for confirmation. First, the linkage with the SNIIRAM National health insurance database allowed confirming 85.7% of investigated cases (ICD-10 codes E11). The sensitivity of SNIIRAM databases is not optimal if used without self-report data: of note, about 10-15% of the French population is covered by other social security regimen and would not be correctly captured by the SNIIRAM databases. Besides, the centralization of SNIIRAM data might take up to a year, leading to delays between reported T2D information and health insurance data.

Second, as regards biological samples, a subsample of participants (n=19,800) were invited to a clinical examination, during which they provided blood and urine samples. We have added details about biological assessments in the Methods. Among the participants who provided blood sample during the clinical/biological examination, 232 had elevated fasting blood glucose (i.e. >1.26 g/L). Among them, 85.3% had consistently declared a T2D diagnosis and/or medication. Elevated blood glucose alone (i.e., without any declaration of T2D diagnosis or treatment) was not considered specific enough to classify the participant as a T2D case.

We have clarified this point in Method C in S1-Appendix and added a paragraph about biological assessments.

Although under-detection of some diagnosed T2D cases is still possible at baseline and during follow-up, it is likely to have been limited thanks to this multi-source strategy for case ascertainment (combining self-report of disease, self-report of medication, link to national health insurance database, and fasting blood glucose on a subsample). The fact that exhaustiveness of T2D case detection cannot be guaranteed (especially those that have never been diagnosed or treated) would mainly have led to reduced statistical power and non-differential classification errors and thus likely to an underestimation of the observed associations. In contrast, it is not likely to explain the statistically significant associations observed. This point has been added in the discussion section page 20.

3)	Confounders. They include different covariates in the models, which is great. But this reviewer believes that there are several more important confounders for the associations. For examples, those did not consume food additives/nitrites are more likely to have healthy lifestyle/dietary quality and lower risk of diabetes. Therefore, adjustment of dietary pattern and dietary quality seems an important step in the main statistical model. In the sensitivity analysis, they additional adjusted for antioxidant intakes as a proxy for fruit and vegie intake. Why not directly adjust fruit and vegie, which is also indicator of lifestyle?

+++ We thank Reviewer #1 for this interesting comment that allowed us to improve our models and further challenge the stability of the results. The adjustment for the “share of ultra-processed food in the overall individual diet” that we had performed in sensitivity analysis #3 was a way to account for an overall dietary pattern. We have now moved this adjustment to the main model. Besides, we added a new sensitivity model (Table D in S1-Appendix, model 2) adjusted for a “Healthy” dietary pattern that we derived by principal component analysis. The results remained unchanged.

As regards fruit and vegetable intakes, since these are the main sources of naturally occurring nitrites and nitrates, we preferred to adjust for components of fruits and vegetables (antioxidant intakes) rather than this food group, to avoid direct collinearity.

4)	Discussion. They should avoid using the word "robust", as they did not have external validation to prove the robustness. They did not adjust for important lifestyle/ diet quality confounders.

+++ We have now removed the word “robust” throughout the discussion, as requested.

Regarding adjustment, as requested by Reviewer #1 and by other comments, the main analysis along with the multiple sensitivity analyses have been upgraded and now include extensive adjustment for 24 socio-demographic, anthropometric, lifestyle, dietary and health parameters. Results remained stable across these analyses.

Reviewer #2: In the study entitled: Nitrites and nitrates dietary exposure from natural sources and food additives and type-2 diabetes risk in the NutriNet-Santé cohort, the authors aimed to investigate the associations between the amount of nitrite and nitrate intakes and their specific food sources (i.e., natural versus additive food sources) and new-onset diabetes, using data of 104168 adults from the French NutriNet-Santé cohort study (2009-2021). They finally concluded that regardless of its sources, higher intake of nitrite exhibited an adverse effect on the development of type 2 diabetes (T2D). No interaction was detected between exposure to nitrate/nitrite and antioxidants (Vitamin C, E and A) in relation to developing T2DM. Use of a national database (i.e., NutriNet-Santé food composition database) for estimation of nutrients, esp. nitrate and nitrites, and assessment of details of commercial names/brand of industrial products to estimate individual additive exposure provides a strong support for estimation of nitrate and nitrites in this population. Although the study design seems well and a relatively-large sample size and use of national databases of food composition are the important strengths of the study, some issues (listed below) may decrease generalizability of the study findings and its reliability.

+++ We thank Reviewer #2 for this overall positive comment.

Major comments:

1.	A low incidence rate of T2D, i.e., lower than 1% over a median of 7.3 years of follow-up (969 cases from 104168 subjects) needs explanation. In addition, the prevalence of T2D is lower in this population even compared to French population (1.6% vs. 6%) as authors acknowledged in their previous study (BMJ 2019;365:l1451; doi: 10.1136/bmj.l1451). Therefore, it should be justified that this cohort is representative of the population. This needs for generalization of data.

+++ One of the main objectives of the NutriNet-Santé cohort is to investigate the relationship between diet and health. NutriNet-Santé is a web-based cohort, and participants are recruited on a voluntary basis. Therefore, NutriNet-Santé does not aim to be representative of the general French population, but instead to have enough contrast between different categories of dietary exposures to conduct etiological analyses, while accounting for a wide diversity of lifestyle profiles. As it is usually the case in population-based cohorts, participants in NutriNet-Santé were younger, more often women, and had higher socio-professional and educational levels than the general (French) population [21]. NutriNet-Santé shows a good representativeness of adults aged below 75 years old, as this population has mostly home access to the Internet [22], and underrepresents participants above 75 years old, who have less access to regular home Internet connection. Therefore, caution is needed when extrapolating results from this cohort to participants aged above 75 years old. Nevertheless, the NutriNet-Santé cohort study provides an access to a broad diversity of populations, including disadvantaged socio-professional categories (for instance, the proportion of unemployed participants (6.4%) is lower than that of the general population (10%) [21], but is higher than what is obtained in most population-based studies using traditional methods of recruitment). NutriNet-Santé’s participants were less likely to smoke [23], and to be overweight or obese (28.2% of men and 29.4% of women in NutriNet-Santé versus 54% and 44% in the French population [23]). In addition, participants in the NutriNet-Santé cohort also had in average healthier dietary habits than the French population (reporting higher intakes of fruit, vegetables, and fish, and lower intakes of red meat and added fats) [24]. These aforementioned factors explain the lower T2D incidence in our cohort (179 cases per 100,000 person-years after age and sex standardization) in comparison with the general French population (289 cases per 100,000 person-years [25]). This overall healthier cohort profile is not specific to the NutriNet-Santé study but generally observed in other well-known large-scale cohorts (e.g. Nurses Health study of Health Professional Follow-up study in the USA, Epic cohort in Europe, etc.). Still, a substantial proportion of participants developed T2D during follow-up and a substantial proportion consumed important amounts of processed meat, nitrites, nitrates, etc. (not all participants had a healthy diet)…, thereby allowing us to perform inter-group comparisons. We have added some discussion about this point in the discussion section page 18:

*‘Second, as in every population-based observational cohort, generalizability of the results to the whole population should be discussed. Compared with the general French population, NutriNet-Santé participants were younger, more often women, with higher educational and socio-professional levels* [21]*. NutriNet-Santé shows a good representativeness of adults aged below 75 years old, as this population has mostly home access to the Internet* [22]*, and underrepresents participants above 75 years old, who have less access to regular home Internet connection. Therefore, caution is needed when extrapolating results from this cohort to participants aged above 75 years old. Nevertheless, the NutriNet-Santé cohort study provides an access to a broad diversity of populations, including disadvantaged socio-professional categories (for instance, the proportion of unemployed participants (6.4%) is lower than that of the general population (10%)* [21]*, but is higher than what is obtained in most population-based studies using traditional methods of recruitment). NutriNet-Santé’s participants were less likely to smoke* [23]*, and to be overweight or obese (28.2% of men and 29.4% of women in NutriNet-Santé versus 54% and 44% in French population* [23]*). These aforementioned factors explain the lower T2D incidence in our cohort (179 cases per 100,000 person-years after age and sex standardization) in comparison with the general French population (289 cases per 100,000 person-years* [25]*). Participants had also healthier dietary habits* [24]*, as they had higher consumption levels of fruit and vegetables and lower levels of processed meats consumption compared to the general population, which led to higher levels of exposure to total nitrites and nitrates and lower levels of exposure to food additives in NutriNet-Santé, compared with nationally representative surveys: indeed, exposure levels in the French INCA2 nationally representative survey were 0.04 mg/kg of body weight (BW) for total nitrites* [26] *and 1.6 mg/kg BW for total nitrates* [27]*, versus 0.09 and 3.34 mg/kg BW in NutriNet-Santé, respectively. Similarly, average intakes of nitrates in our study were higher than those reported in a systematic review by Babateen et al.* [28]*: average intake of total nitrates in healthy European populations = 107.21 mg/d vs. 213.2 mg/d in our study. In contrast, median intakes of nitrites and nitrates in an Iranian study were higher than those in our study (410 mg/d vs. 194 mg/d in our study for nitrates and 8.8 mg/d vs. 5.0 mg/d in our study for nitrites)* [29]*. As regards food additives specifically, exposure levels were 0.01 to 0.04 mg/kg BW for nitrites from additives and 0.05 to 0.10 mg/kg BW for nitrates from additives in the multi-country EFSA report, versus 0.004 mg/kg BW and 0.003 mg/kg BW in NutriNet-Santé, respectively. This might have led to an underestimation of true associations with nitrites and nitrates from food additives (due to the reduced contrast between compared groups). In contrast, regarding foods and water-originated nitrites and nitrates, the distribution of exposure in our population allowed us to have enough high consumers to properly explore this relatively extreme part of the curve, which exists (to a lesser extent) in the general population.’*

2.	Another concern about the study findings is that the main outcome was self-reported, however the authors claimed that self-report of T2D was tried to be confirmed by supportive data such as National health insurance (SNIIRAM) database or measurement of fasting blood glucose in a sub-sample of the participants (the number of participants with FBS was not reported). Nevertheless, SNIIRAM database only confirmed T2D diagnosis of about 80% of the cases surveyed, and among those who had biochemical measurements, only 85.3 % of those with elevated fasting blood glucose had consistently reported a diagnosis of T2DM and/or medication. Reporting bias of the outcome in this study seem resulted in a considerable underestimation of the outcome that seriously may affect validity and reliability of the study findings. This important issue, therefore, should be discussed by providing a number of relevant evidence indicated that whether such limitation can affect the study findings validity. They should also support their findings by providing national data regarding the prevalence and annual incidence if diabetes in their country. I wonder if undiagnosed type diabetes has been considered in this study.

+++ As explained in our response to Reviewer #2, point 1*,* as it is usually the case in population-based cohorts, *“participants in NutriNet-Santé were younger, more often women, and had higher socio-professional and educational levels than the general (French) population* [21]*. They were also less likely to smoke* [23]*, and to be overweight or obese (28.2% of men and 29.4% of women in NutriNet-Santé versus 54% and 44% in the French population* [23]*). In addition, participants in the NutriNet-Santé cohort also had in average healthier dietary habits than the French population (reporting higher intakes of fruit, vegetables, and fish, and lower intakes of red meat and added fats)* [24]*. This explains the lower T2D incidence in our cohort (179 cases per 100,000 person-years after age and sex standardization) in comparison with the general French population (289 cases per 100,000 person-years* [25]*).*

This overall healthier cohort profile is not specific to the NutriNet-Santé study but generally observed in other well-known large-scale cohorts (e.g. Nurses Health study of Health Professional Follow-up study in the USA, Epic cohort in Europe, etc.).”

Besides, as explained above in our response to Reviewer #1, point 2: “*T2D case ascertainment was based on self-report, but was confirmed using a multi-source strategy. Reporting both incident T2D and the use of T2D medication via the baseline and health follow-up questionnaire (administered every 3 months) or the dedicated health events platform, was mandatory for the case to be considered as a confirmed T2D case. The questions were: “Have you been diagnosed with T2D (if yes, indicate the date of diagnosis)” and “Are you treated for T2D ?”. Participants are asked to record all currently taken medications (T2D medications and others). A search engine with embedded exhaustive Vidal® drug database is used to facilitate medication data entry for the participants. ATC codes considered for T2D medication were A10AB01, A10AB03, A10AB04, A10AB05, A10AB06, A10AC01, A10AC03, A10AC04, A10AD01, A10AD03, A10AD04, A10AD05, A10AE01, A10AE02, A10AE03, A10AE04, A10AE05, A10AE30, A10BA02, A10BB01, A10BB03, A10BB04, A10BB06, A10BB07, A10BB09, A10BB12, A10BD02, A10BD03, A10BD05, A10BD07, A10BD08, A10BD10, A10BD15, A10BD16, A10BF01, A10BF02, A10BG02, A10BG03, A10BH01, A10BH02, A10BH03, A10BX02, A10BX04, A10BX07, A10BX09, A10BX10, A10BX11, A10BX12.*

*Following a T2D diagnosis and/or medication declaration, two additional sources of information were considered for confirmation. First, the linkage with the SNIIRAM National health insurance database allowed confirming 85.7% of investigated cases (ICD-10 codes E11). The sensitivity of SNIIRAM databases is not optimal if used without self-report data: of note, about 10-15% of the French population is covered by other social security regimen and would not be correctly captured by the SNIIRAM databases. Besides, the centralization of SNIIRAM data might take up to a year, leading to delays between reported T2D information and health insurance data.*

*Second, as regards biological samples, a subsample of participants (n=19,800) were invited to a clinical examination, during which they provided blood and urine samples. We have added details about biological assessments in the Methods. Among the participants who provided blood sample during the clinical/biological examination, 232 had elevated fasting blood glucose (i.e. >1.26 g/L). Among them, 85.3% had consistently declared a T2D diagnosis and/or medication. Elevated blood glucose alone (i.e., without any declaration of T2D diagnosis or treatment) was not considered specific enough to classify the participant as a T2D case. We have clarified this point in Method C in S1-Appendix.*

*Although under detection of some diagnosed T2D cases is still possible at baseline and during follow-up, it is likely to have been limited thanks to this multi-source strategy for case ascertainment (combining self-report of disease, self-report of medication, link to national health insurance database, and fasting blood glucose on a subsample). The fact that exhaustiveness of T2D case detection cannot be guaranteed (especially those that have never been diagnosed or treated) would mainly have led to reduced statistical power and non-differential classification errors and thus likely to an underestimation of the observed associations. In contrast, it is not likely to explain the statistically significant associations observed. This point has been added in the discussion section page 20.”*

3.	Another issue is existing of an imbalanced-proportion of male and female (79.1% female). Furthermore, as the authors reported, compared with the general French population, NutriNet-Santé participants were younger (included of subjects > 15y), with higher educational and socio-professional levels and healthier dietary habits. They also had higher consumption levels of fruit and vegetables and lower levels of processed meats consumption. These may result in a bias in estimation of both exposure and outcome. As indicated by the authors (P12, lines 328-330), the characteristics of the study population might have led to an underestimation of associations with nitrites/nitrates from food additives, and an overestimation of those with nitrites and nitrates from natural sources.

+++ Please refer to our response to point 1 (Reviewer #2), in which we grouped and detailed our answers to the questions regarding representativity and generalizability.

4.	A practical approach to response to some of the aforementioned concerns may be excluding younger adults (<30 y) from the analyses, and also conducting sensitivity analyses by separating male and female (use of sex-specific tertiles of exposure that were considered in categorizing nitrate/nitrite levels could not per se eliminate the concern).

+++ We verified that there was no statistically significant interaction between the studied exposures and sex regarding T2D risk (all P for interaction > 0.1). This has been added to the Methods page 11 and Results page 14.

However, in order to comply with Reviewer #2’s request, we have now tested a sensitivity analysis by excluding participants aged <30 years old (Table D in S1-Appendix, model 5). Results were not substantially modified.

Besides, despite a non-significant interaction test (p>0.1), sex-specific models were performed: in women, associations were found between total nitrites (HR_tertile 3 vs. 1_ = 1.47 (1.14-1.90), P_-trend_=0.002), nitrites from additives (HR_higher consumers vs. non-consumers_ = 1.36 (1.06-1.75), P_-trend_=0.006) and foods and water-originated nitrites ((HR_tertile 3 vs. 1_ = 1.50 (1.15-1.94), P_-trend_=0.002). In men, associations were only significant for nitrites from food additives (HR_higher consumers vs. non-consumers_ = 1.65 (1.15-2.36), P_-trend_=0.009).

These results were presented in page 14 and Table C in S1-Appendix.

5.	The authors considered both food products and water as naturally occurring sources of nitrite (or non-additive sources); I have concern about such categorization, because nitrite is considered as a contaminant agent in drinking water that is originated from fertilizers through run-off water, sewage, and mineral deposits. Labeling that nitrite, therefore, as natural source-originated may be misleading. A good suggestion may be separating sources of nitrite as foods-originated, water-originated and additives-originated, however, the estimated nitrite intake from drinking water was negligible (< 0.01% compared to 95.3% of total nitrite intakes from food sources).

+++ We agree that the term “natural” may be misleading. Thus, we have now changed the name of the categories as recommended. Foods and water-originated nitrites had to be pooled since the latter was too low to be considered separately, as pointed out by Reviewer #2. The names of the categories are now: 1) foods and water-originated nitrates/nitrites (referring to non-additive exposures), and additives-originated nitrates/nitrites. This has been clarified in the Methods page 8 and changed throughout the manuscript.

6.	Although a main strength of the study is using a web-based self-administered 24h-dietary records (repeated at 6-month intervals) with a valid and reliable estimations of dietary intakes (validated by face-to-face dietician interview, and biological biomarkers), under-reporting of energy intakes (about 17%, as reported in the study flowchart) is questionable.

+++ Energy under-reporting is a classical phenomenon in nutrition studies and cohorts. The prevalence of energy under-reports in our study (17%) is in the range of those reported in other similar studies around the world: for instance, the prevalence of under-reporters ranged between 3 and 20% in the multicentric European EPIC cohort [30], 25.1% in the American NHANES study [31], and 18% in the Norwegian Breast Cancer Screening Program [32]. In the nationally representative INCA 3 study conducted in 2016 by the French Food Safety Agency [33] 18% of adults participants were identified as under-reporters using the Black method. We have added the detailed methodology for under-energy reporting detection in Method A in S1-Appendix.

Besides, we have now compared baseline characteristics between under-reporters and the participants included in our study sample. These results are presented in Method A in S1-Appendix, and we have discussed these points in the discussion, page 19:

*‘**As classically observed in nutritional epidemiology studies, a significant sub-sample of the cohort (17%) was flagged as energy under-reporters and were excluded from the final sample. Different reasons might explain this phenomenon, mainly social desirability bias, but also input errors while using the online tool. These participants usually declare abnormally low energy intakes, implausible even in case of restrictive diets. Participants were also asked whether they were following any caloric restriction; in that case, they were included back in the analyses. This ensured that flagged under-reporters have true incoherent reporting, and must be excluded. The prevalence of energy under-reports in our study (17%) is in the range of those reported in other similar studies around the world: for instance, the prevalence of under-reporters ranged between 3 and 20% in the multicentric European EPIC cohort* [30]*, 25.1% in the American NHANES study* [31]*, and 18% in the Norwegian Breast Cancer Screening Program* [32]*. In the nationally representative INCA 3 study conducted in 2016 by the French Food Safety Agency* [33] *18% of adults participants were identified as under-reporters using the Black method.* *Under-reporters in our study were older, were more inclined to be male and current smokers, to have a higher BMI and alcohol intake, and a lower educational level and monthly income. Although their exclusion may limit the generalizability of the findings, it was necessary, in order to avoid important exposure classification bias.*

7.	The authors claimed that they used the French official regional sanitary control of tap water to estimate intakes of nitrite from drinking water. They need to provide more details regarding individual estimation of water-originated nitrite.

+++ Indeed, the French official regional sanitary control of tap water was used to estimate intakes via water consumption, by region of residence [34]. We have added this information to the Methods section page 8.

*‘The French official regional sanitary control of tap water was used to estimate intakes via water consumption, by region of residence* [34]*: SISE-Eaux is a French governmental database including data on quality control of tap water in the 34,955 French municipalities. It is coordinated by the Regional Health Agencies and consists of a collection of 310,000 samples per year (12 million samples overall). Data is available publicly* [34] *and a municipality-specific merge according to the NutriNet-Santé participants’ postal code has been performed, as well as a dynamic temporal merge according to the year of dietary records.’*

Besides, we have now provided the detail of water and food-originated nitrates and nitrites separately in Table 1.

8.	In the introduction section, authors stated that "In the mouth, much of the ingested exogenous nitrate is reduced to nitrite by commercial bacteria…."; this sentence need to be corrected as of ingested nitrate, ~25 % is extracted by the salivary glands and of which ~20 % is converted nitrite. It means that about 5 % of ingested nitrate is converted to nitrite in the oral cavity. This nitrite then is converted to NO in the stomach providing a continuous source of NO for human body. (see PMID: 35391922 for more details).

+++ We have edited this statement accordingly. It now reads:

*“In the mouth, about 5% of the ingested exogenous nitrate is reduced to nitrite by commensal bacteria residing in that area, and is then converted to nitric-oxide (NO) in the stomach, through the nitrate-nitrite-NO pathway, providing a continuous source of NO for human body, in addition to NO production through the L-arginine oxidative pathway* [35,36]*.”*

9.	In the introduction authors state that eNOS is responsible for endogenous nitrate regulation; although eNOS is considered as the main source of NO production, which is converted to nitrite and then nitrate, the sentence is not factually correct that eNOS regulated nitrate.

+++ We have edited this statement accordingly. It now reads:

*“For instance,* *in mice lacking endothelial NO synthase enzymes (eNOS) (considered as the main source of endogenous nitric oxide (NO) production, through the L-arginine oxidative pathway) at high risk of metabolic syndrome- like phenotype, nitrate supplementation, allowing the endogenous production of NO by the sequential reduction of nitrate to nitrite and then NO, through the alternative nitrate-nitrite-NO pathway, reduced body weight, improved glucose tolerance, and decreased plasma triglycerides and levels of glycated hemoglobin (HbA1c).”*

10.	Authors need to compare nitrate/nitrite intakes in this population with those from other populations (see for example, Babateen AM, Fornelli G, Donini LM, Mathers JC, Siervo M. Assessment of dietary nitrate intake in humans: a systematic review. Am J Clin Nutr. 2018;108:878-88).

+++ We thank the Reviewer for pointing out this publication.

As stated in our responses to Reviewer 2 point 1, the corresponding paragraph of the discussion page 18 now reads:

*“Participants had also healthier dietary habits* [24]*, as they had higher consumption levels of fruit and vegetables and lower levels of processed meats consumption compared to the general population, which led to higher levels of exposure to total nitrites and nitrates and lower levels of exposure to food additives in NutriNet-Santé, compared with nationally representative surveys: indeed, exposure levels in the French INCA2 nationally representative survey were 0.04 mg/kg of body weight (BW) for total nitrites* [26] *and 1.6 mg/kg BW for total nitrates* [27]*, versus 0.09 and 3.34 mg/kg BW in NutriNet-Santé, respectively. Similarly, average intakes of nitrates in our study were higher than those reported in a systematic review by Babateen et al.* [28]*: average intake of total nitrates in healthy European populations = 107.21 mg/d vs. 213.2 mg/d in our study. In contrast, median intakes of nitrites and nitrates in an Iranian study were higher than those in our study (410 mg/d vs. 194 mg/d in our study for nitrates and 8.8 mg/d vs. 5.0 mg/d in our study for nitrites)* [29]*. As regards food additives specifically, exposure levels were 0.01 to 0.04 mg/kg BW for nitrites from additives and 0.05 to 0.10 mg/kg BW for nitrates from additives in the multi-country EFSA report, versus 0.004 mg/kg BW and 0.003 mg/kg BW in NutriNet-Santé, respectively. This might have led to an underestimation of true associations with nitrites and nitrates from food additives (due to the reduced contrast between compared groups). In contrast, regarding foods and water-originated nitrites and nitrates, the distribution of exposure in our population allowed us to have enough strong consumers to properly explore this extreme part of the curve, which exists (to a lesser extent) in the general population.”*

11.	Page 8, line 213, authors state that date of an incident type-1 diabetes was considered for person time calculation, please provide data on incidence of T1D in the population.

+++ In all, 88 incident cases of T1D were detected during follow-up. The crude incidence rate of T1D in our sample was 12.3 for 100,000 person-years. Since the outcome of interest was T2D for this study, these incident T1D cases were censored at the date of diagnosis. These precisions have been added page 10.

Minor comments

1.	In the most Figures presented in the supplementary material, Y-axis is not started from zero. I strongly suggested that in all cases the start oy Y-axis to be zero.

+++ Unfortunately, these settings are the default settings of the used Macro, and cannot be modified. The important Y-value for these plots is 1 (corresponding to a HR of 1, and to a null-association), and a HR of 0 is not relevant nor plausible.

2.	Page 10, lines 272-273: "As regards nitrites from food additives, the curve showed a logarithmic shape 273 with a plateau in the highly exposed participants (p for non-linearity =0.0009)". I did not find a figure in supplementation with logarithmic scales. Please add such a figure.

+++ We apologize for the inaccuracy in our wording. We have now replaced this statement with:

*"As regards nitrites from food additives, the curve showed a non-linear shape with a plateau in the highly exposed participants (p for non-linearity < 0.0001)".* As requested by the Editor, RCS curves are now presented in the main manuscript, figure 4.

3.	I suggest that authors measure urine nitrate and nitrate in sub-sample to provide better indication of exposure to nitrate and nitrite.

+++ As explained in our response to Reviewer #1, point 1: *“The accuracy of the qualitative and quantitative food additives exposure assessment of the NutriNet-Santé cohort represents an important strength of the study, thanks to detailed and repeated 24h-dietary records, links to multiple food composition databases (OQALI* [11]*, Open Food Facts* [12]*, GNPD* [13]*, EFSA* [14]*, and GSFA* [15]*), ad-hoc laboratory assays, and dynamic matching to account for reformulations of industrial food items over time* [16]*. We fully agree with Reviewer #1 that validation of additive exposure versus plasmatic or urinary biomarkers would be ideal, however, it is not possible for many food additives, among which nitrites and nitrates.*

*Indeed, specific validation of nitrites/nitrates intakes versus biomarkers has been envisioned at the beginning of the ADDITIVES ERC Program, but after careful examination of biomarker literature and scientific discussions among partners, it has been judged not feasible. Indeed, as for many food additives, it was not possible to identify markers in blood or urine that would be specific enough to reflect exogenous dietary exposure and not endogenous metabolism. More specifically, for instance, plasma nitrate would not only be a suitable marker for dietary sources, but would also reflect nitrate derived from various individual (patho‐) physiological pathways, as well as the individual capacity to eliminate nitrate* [17–19]*. Similarly for urinary nitrites, in addition to the fact that urinary tract infections are an important driver of urine nitrites levels* [20]*, therefore providing biased estimates of dietary exposure.*

*We have added these elements to the discussion page 20:*

*‘Lastly, exposure to nitrates and nitrates has not been validated with blood or urine assays due to lack of biomarkers that would be specific enough to reflect exogenous dietary exposure and not endogenous metabolism. For instance, plasma nitrate would not only be a suitable marker for dietary sources, but would also reflect nitrate derived from various individual (patho‐) physiological pathways, as well as the individual capacity to eliminate nitrate* [17–19]*. Similarly for urinary nitrites, in addition to the fact that urinary tract infections are an important driver of urine nitrites levels* [20]*, therefore providing biased estimates of dietary exposure.’”*

Reviewer #3: The authors investigated the possible associations between nitrite/nitrate intakes and their sources with development of type 2 diabetes in the framework of the French NutriNet-Santé cohort study (2009-2021). They reported an estimated population-mean intake of 194 and 5 mg/d for nitrate and nitrite, respectively and concluded that higher intake of nitrite from both natural and food additive sources increased risk of type 2 diabetes. Furthermore, they reported that there was no interaction between nitrate/nitrite intakes and antioxidants in relation to the development of diabetes, unlike those previously reported in a population-based cohort for nitrite and vitamin C.

One of the main strength of the study was use of a national database for estimation of nitrate and nitrites, and assessment of details of commercial names/brand of industrial products to estimate individual additive exposure provides a strong support for estimation of nitrate and nitrites in this population. Some issues, however, should be considered for potential further evaluation:

1.	A low incidence rate of diabetes (~0.09%) during the study follow-up, an imbalanced-proportion of male and female in the study (79.1% female), including adolescents in the study participants, and self-reporting of the main outcome which seems resulted in an underestimation of the outcome, may decrease validity and reliability of the study findings.

2.	The authors discussed that the study participants had higher educational and socio-professional levels and healthier dietary habits (e.g., higher consumption levels of fruit and vegetables and lower levels of processed meats consumption); these characteristics may seriously affect estimation of nitrites/nitrates intakes from food additives that was reported about 4.7% of total intakes.

+++ Answers to questions 1 and 2:

As explained in our response to Reviewer #2, point 1:

*+++ One of the main objectives of the NutriNet-Santé cohort is to investigate the relationship between diet and health. NutriNet-Santé is a web-based cohort, and participants are recruited on a voluntary basis. Therefore, NutriNet-Santé does not aim to be representative of the general French population, but instead to have enough contrast between different categories of dietary exposures to conduct etiological analyses, while accounting for a wide diversity of lifestyle profiles. As it is usually the case in population-based cohorts, participants in NutriNet-Santé were younger, more often women, and had higher socio-professional and educational levels than the general (French) population* [21]*. NutriNet-Santé shows a good representativeness of adults aged below 75 years old, as this population has mostly home access to the Internet* [22]*, and underrepresents participants above 75 years old, who have less access to regular home Internet connection. Therefore, caution is needed when extrapolating results from this cohort to participants aged above 75 years old. Nevertheless, the NutriNet-Santé cohort study provides an access to a broad diversity of populations, including disadvantaged socio-professional categories (for instance, the proportion of unemployed participants (6.4%) is lower than that of the general population (10%)* [21]*, but is higher than what is obtained in most population-based studies using traditional methods of recruitment). NutriNet-Santé’s participants were less likely to smoke* [23]*, and to be overweight or obese (28.2% of men and 29.4% of women in NutriNet-Santé versus 54% and 44% in the French population* [23]*). In addition, participants in the NutriNet-Santé cohort also had in average healthier dietary habits than the French population (reporting higher intakes of fruit, vegetables, and fish, and lower intakes of red meat and added fats)* [24]*. These aforementioned factors explain the lower T2D incidence in our cohort (179 cases per 100,000 person-years after age and sex standardization) in comparison with the general French population (289 cases per 100,000 person-years* [25]*). This overall healthier cohort profile is not specific to the NutriNet-Santé study but generally observed in other well-known large-scale cohorts (e.g. Nurses Health study of Health Professional Follow-up study in the USA, Epic cohort in Europe, etc.). Still, a substantial proportion of participants developed T2D during follow-up and a substantial proportion consumed important amounts of processed meat, nitrites, nitrates, etc. (not all participants had a healthy diet)…, thereby allowing us to perform inter-group comparisons. We have added some discussion about this point in the discussion section page 18:*

*‘Second, as in every population-based observational cohort, generalizability of the results to the whole population should be discussed. Compared with the general French population, NutriNet-Santé participants were younger, more often women, with higher educational and socio-professional levels* [21]*. NutriNet-Santé shows a good representativeness of adults aged below 75 years old, as this population has mostly home access to the Internet* [22]*, and underrepresents participants above 75 years old, who have less access to regular home Internet connection. Therefore, caution is needed when extrapolating results from this cohort to participants aged above 75 years old. Nevertheless, the NutriNet-Santé cohort study provides an access to a broad diversity of populations, including disadvantaged socio-professional categories (for instance, the proportion of unemployed participants (6.4%) is lower than that of the general population (10%)* [21]*, but is higher than what is obtained in most population-based studies using traditional methods of recruitment). NutriNet-Santé’s participants were less likely to smoke* [23]*, and to be overweight or obese (28.2% of men and 29.4% of women in NutriNet-Santé versus 54% and 44% in French population* [23]*). These aforementioned factors explain the lower T2D incidence in our cohort (179 cases per 100,000 person-years after age and sex standardization) in comparison with the general French population (289 cases per 100,000 person-years* [25]*). Participants had also healthier dietary habits* [24]*, as they had higher consumption levels of fruit and vegetables and lower levels of processed meats consumption compared to the general population, which led to higher levels of exposure to total nitrites and nitrates and lower levels of exposure to food additives in NutriNet-Santé, compared with nationally representative surveys: indeed, exposure levels in the French INCA2 nationally representative survey were 0.04 mg/kg of body weight (BW) for total nitrites* [26] *and 1.6 mg/kg BW for total nitrates* [27]*, versus 0.09 and 3.34 mg/kg BW in NutriNet-Santé, respectively. Similarly, average intakes of nitrates in our study were higher than those reported in a systematic review by Babateen et al.* [28]*: average intake of total nitrates in healthy European populations = 107.21 mg/d vs. 213.2 mg/d in our study. In contrast, median intakes of nitrites and nitrates in an Iranian study were higher than those in our study (410 mg/d vs. 194 mg/d in our study for nitrates and 8.8 mg/d vs. 5.0 mg/d in our study for nitrites)* [29]*. As regards food additives specifically, exposure levels were 0.01 to 0.04 mg/kg BW for nitrites from additives and 0.05 to 0.10 mg/kg BW for nitrates from additives in the multi-country EFSA report, versus 0.004 mg/kg BW and 0.003 mg/kg BW in NutriNet-Santé, respectively. This might have led to an underestimation of true associations with nitrites and nitrates from food additives (due to the reduced contrast between compared groups). In contrast, regarding foods and water-originated nitrites and nitrates, the distribution of exposure in our population allowed us to have enough high consumers to properly explore this relatively extreme part of the curve, which exists (to a lesser extent) in the general population.’*

Besides, as stated in our response to Reviewer #2, point 4:

*“We verified that there was no interaction between the studied exposures and sex regarding T2D risk (all P for interaction > 0.1). This has been added to the Methods page 11 and Results page 14.*

*However, in order to comply with Reviewer #2’s request, we have now tested a sensitivity analysis by excluding participants aged <30 years old (Table D in S1-Appendix, model 5). Results were not substantially modified.*

*Besides, despite non-significant interactions with sex (p>0.1), sex-specific models were performed: in women, associations were found between total nitrites (HR_tertile 3 vs. 1_ = 1.47 (95% CI 1.14-1.90), P_-trend_=0.002), nitrites from additives (HR_higher consumers vs. non-consumers_ = 1.36 (95% CI 1.06-1.75), P_-trend_=0.006) and foods and water-originated nitrites ((HR_tertile 3 vs. 1_ = 1.50 (95% CI 1.15-1.94), P_-trend_=0.002). In men, associations were only significant for nitrites from food additives (HR_higher consumers vs. non-consumers_ = 1.65 (95% CI 1.15-2.36), P_-trend_=0.009). These results were presented in page 14 and Table C in S1-Appendix.“*

Last, as presented in our response to Reviewer #1, point 2: “*T2D case ascertainment was based on self-report, but was confirmed using a multi-source strategy. Reporting both incident T2D and the use of T2D medication via the baseline and health follow-up questionnaire (administered every 3 months) or the dedicated health events platform, was mandatory for the case to be considered as a confirmed T2D case. The questions were: “Have you been diagnosed with T2D (if yes, indicate the date of diagnosis)” and “Are you treated for T2D ?”. Participants are asked to record all currently taken medications (T2D medications and others). A search engine with embedded exhaustive Vidal® drug database is used to facilitate medication data entry for the participants. ATC codes considered for T2D medication were A10AB01, A10AB03, A10AB04, A10AB05, A10AB06, A10AC01, A10AC03, A10AC04, A10AD01, A10AD03, A10AD04, A10AD05, A10AE01, A10AE02, A10AE03, A10AE04, A10AE05, A10AE30, A10BA02, A10BB01, A10BB03, A10BB04, A10BB06, A10BB07, A10BB09, A10BB12, A10BD02, A10BD03, A10BD05, A10BD07, A10BD08, A10BD10, A10BD15, A10BD16, A10BF01, A10BF02, A10BG02, A10BG03, A10BH01, A10BH02, A10BH03, A10BX02, A10BX04, A10BX07, A10BX09, A10BX10, A10BX11, A10BX12.*

*Following a T2D diagnosis and/or medication declaration, two additional sources of information were considered for confirmation. First, the linkage with the SNIIRAM National health insurance database allowed confirming 85.7% of investigated cases (ICD-10 codes E11). The sensitivity of SNIIRAM databases is not optimal if used without self-report data: of note, about 10-15% of the French population is covered by other social security regimen and would not be correctly captured by the SNIIRAM databases. Besides, the centralization of SNIIRAM data might take up to a year, leading to delays between reported T2D information and health insurance data.*

*Second, as regards biological samples, a subsample of participants (n=19,800) were invited to a clinical examination, during which they provided blood and urine samples. We have added details about biological assessments in the Methods. Among the participants who provided blood sample during the clinical/biological examination, 232 had elevated fasting blood glucose (i.e. >1.26 g/L). Among them, 85.3% had consistently declared a T2D diagnosis and/or medication. Elevated blood glucose alone (i.e., without any declaration of T2D diagnosis or treatment) was not considered specific enough to classify the participant as a T2D case. We have clarified this point in Method C in S-Appendix.*

*Although under detection of some diagnosed T2D cases is still possible at baseline and during follow-up, it is likely to have been limited thanks to this multi-source strategy for case ascertainment (combining self-report of disease, self-report of medication, link to national health insurance database, and fasting blood glucose on a subsample). The fact that exhaustiveness of T2D case detection cannot be guaranteed (especially those that have never been diagnosed or treated) would mainly have led to reduced statistical power and non-differential classification errors and thus likely to an underestimation of the observed associations. In contrast, it is not likely to explain the statistically significant associations observed. This point has been added in the discussion section page 20.*”

3.	The other confounders such as hypertension, history of CVD, TG/HDL-C and FPG level did not consider in their data analysis.

+++ We have now added in a sensitivity model further adjustment for prevalent hypertension, CVD and hypertriglyceridemia. The associations were slightly attenuated but remained significant.

This has been added in the Methods section page 11 and the results shown in Table D in S1-Appendix (model 4).

4.	I do not agree with the authors regarding their definition about naturally occurring sources of nitrite (food products + water); nitrite is a contaminant agent in drinking water and categorizing it as a natural-originated compound is not acceptable. Moreover, I have some concerns about defining sources of nitrate/nitrite at all; as illustrated in Appendix 4a (Natural food (solid) sources of nitrites and nitrates) and Appendix 4b (Food sources of nitrites as food additives), "processed meats" were considered in both categories. Please be clearer about defining sources of nitrite and nitrate.

+++ As also requested by Reviewer #2, point 5: *“We agree that the term “natural” may be misleading. Thus, we have now changed the name of the categories as recommended. The names of the categories are now: 1) foods and water-originated nitrates/nitrites (referring to non-additive exposures), and additives-originated nitrates/nitrites. This has been clarified in the Methods page 8 and changed throughout the manuscript.”*

As regards meat products, in fact, they are sources of both “foods-originated nitrates/nitrites” (even before adding nitrates/nitrites additives, since animals consume themselves sources of nitrates/nitrites such as contaminated water or plant-based foods) and “nitrates/nitrites from food additives”. We have clarified this point in the Results section.

5.	The authors should also examine the association between dietary exposure to nitrites/nitrates with incident prediabetes or metabolic syndrome.

+++ We agree with Reviewer #3 that this would be of high interest but it was not possible as such in the cohort. Indeed, FPG, TG, HDL and waist circumference were only measured at one time-point during the clinical examination, and only for a sub-sample of participants (n=16,450 having valid dietary data for nitrites and nitrates).

However, in order to comply with Reviewer #3’s request, we performed a cross-sectional analysis of the association between nitrites/nitrates intake and clinically measured metabolic syndrome on this sub-sample. Statistically significant associations were found for total nitrites (OR _tertile 3 vs. 1_ = 1.35 (95% CI 1.16-1.58), P_-trend_<0.001), and foods and water-originated nitrites (OR _tertile 3 vs. 1_ = 1.45 (95% CI 1.24-1.71), P_-trend_<0.001), but not for additives-originated nitrites (OR _tertile 3 vs. 1_ = 1.06 (95% CI 0.90-1.25), P_-trend_ = 0.5), or nitrates (P_-trend_ >0.1) However, the evidence that can be drawn out of these findings is weak, given the reduced sample size and the absence of a prospective design. This information has been added in the Methods section (page 11) and results are presented in Table F in S1-Appendix.

6.	The authors need to make a comparison between their estimations about dietary intakes of nitrate/nitrite with other populations (especially European populations) and probably previous estimations from their country.

+++ We have extended the discussion section page 18 with such information, as detailed above in our responses to point 1&2 (Reviewer#3).

7.	Regardless of using a validated method for estimating dietary intakes, the percent of the study participants that were excluded from the study due to under-report of energy intakes (about 17%), is considerable. Please discuss the issue and provide potential reasons, and address whether this missing information may affect the study generalizability. I strongly suggest that the authors make a comparison between “reporters and other participant.

+++ As stated in our response to Reviewer #2, point 6: *“Energy under-reporting is a classical phenomenon in nutrition studies and cohorts. The prevalence of energy under-reports in our study (17%) is in the range of those reported in other similar studies around the world: for instance, the prevalence of under-reporters ranged between 3 and 20% in the multicentric European EPIC cohort* [30]*, 25.1% in the American NHANES study* [31]*, and 18% in the Norwegian Breast Cancer Screening Program* [32]*. In the nationally representative INCA 3 study conducted in 2016 by the French Food Safety Agency* [33] *18% of adults participants were identified as under-reporters using the Black method. We have added the detailed methodology for under-energy reporting detection in Method A in S1-Appendix.*

*Besides, we have now compared baseline characteristics between under-reporters and the participants included in our study sample. These results are presented in Method A in S1-Appendix, and we have discussed these points in the discussion, page 19:*

*‘‘As classically observed in nutritional epidemiology studies, a significant sub-sample of the cohort (17%) was flagged as energy under-reporters and were excluded from the final sample. Different reasons might explain this phenomenon, mainly social desirability bias, but also input errors while using the online tool. These participants usually declare abnormally low energy intakes, implausible even in case of restrictive diets. Participants were also asked whether they were following any caloric restriction; in that case, they were included back in the analyses. This ensured that flagged under-reporters have true incoherent reporting, and must be excluded. The prevalence of energy under-reports in our study (17%) is in the range of those reported in other similar studies around the world: for instance, the prevalence of under-reporters ranged between 3 and 20% in the multicentric European EPIC cohort* [30]*, 25.1% in the American NHANES study* [31]*, and 18% in the Norwegian Breast Cancer Screening Program* [32]*. In the nationally representative INCA 3 study conducted in 2016 by the French Food Safety Agency* [33] *18% of adults participants were identified as under-reporters using the Black method.* *Under-reporters in our study were older and were more inclined to be male and current smokers, to have a higher BMI and alcohol intake, and a lower educational level and monthly income. Although their exclusion may limit the generalizability of the findings, it was necessary, in order to avoid important exposure classification bias, which is more crucial than a potential selection bias, given that the main aim of our cohort was not to achieve representativeness of the general population.”*

Reviewer #4: See attachment.

This is a very large well done prospective study evaluating the association between intake of nitrates, and nitrates and risk of type two diabetes over a seven-year follow up. This is an important and understudied area, and the authors also evaluate nitrates/nitrites from food additives separately, which is an added strength. The major limitation that limits the interpretation and validity of the conclusions is the lack of data on mouthwash use, which may likely be a major confounder leading to the lack of association see between intake of nitrates (which is mostly from vegetables) and diabetes. The study also has a few other limitations as described below.

+++ We thank Reviewer #4 for this overall positive comment.

The question regarding mouthwash use is addressed below.

1.	The main concern of the 24 hour diet records is the day to day variability in diet and limitation of diet records to capture long-term intake, which may be partly countered by multiple diet records over time that were averaged. Importantly, in the Author Summary, they imply in line 76 that the semi quantitative food frequency questionnaire used by another study is a limitation, whereas it is really a strength. This should be reworded, and the pros and cons of the dietary assessment methods need to be mentioned in the discussion.

+++ We totally agree with Reviewer #4 that each dietary assessment tool presents advantages and limitations. FFQs are cost-effective tools that have been shown to be particularly adapted to assess usual dietary habits in terms of broad food groups, and (semi)quantitative FFQs can provide reliable estimates of nutrient intakes [37]. Dietary records or recalls are generally more costly, but have been recognized as appropriate tools to capture usual dietary habits, when administered in a repeated manner [38]; in addition, estimates from repeated dietary records showed better correlations with urinary biomarkers due to their greater level of detail in the collected dietary data [39]. More specifically regarding food additives, even though FFQs could provide interesting insights into usual nutritional exposure at a “generic” food group level, they are less appropriate to properly evaluate exposure to specific food additives coming from several commercial products with different brands, as available in the NutriNet-Santé’s dietary records. In addition to the 3,500 generic food items, we collected specific information about the consumed commercial brands/names, which is crucial given the heterogeneity of food additives between the available brands. Furthermore, the several repeated 24h dietary records (6 on average, maximum 18) allowed us to capture dietary habits. As requested by Reviewer #1, point 1, we verified that the results remained unchanged when the minimal number of dietary records was increased to 3 (sensitivity analyses, Table D in S1-Appendix, model 6).

We have now rephrased the statement in the Author Summary, which now reads:

*However, this study made no difference between foods and water-originated nitrites and food additives-originated nitrites, and did not collect data on specific consumed brands.*

We have also discussed these 2 tools in the discussion section, page 15:

*“**The Iranian study assessed diet using food frequency questionnaires, which are recognized to provide reliable estimates of dietary habits in terms of “generic” food group consumption* [37]*. Dietary records are also recognized as appropriate tools to capture usual dietary habits, when administered in a repeated manner (as done in the present study: mean number of records/participant=6, maximum=18)* [38,39]. *Besides, records collecting brand-specific information for commercial products provide greater accuracy to evaluate exposure to specific food additives coming from several commercial products with different brands (given the substantial variation of additive content between commercial brands)”.*

2.	The authors have attempted to separate nitrate/nitrite sources. However, they have still combined water contamination, processed meat, seasoning, and intake from vegetables, into a category called “natural sources”. The word “natural” does not seem appropriate for water contamination, processed meat, and seasoning. Importantly, it would add a lot of value to separate these sources and evaluate the individual impacts from vegetables, especially since beetroots and green leafy vegetables (two potentially beneficial vegetables) are the main sources of dietary nitrate.

+++ As stated in our answer to Reviewer #2, point 5, we have now renamed the two categories into 1) foods and water-originated nitrates/nitrites, and additives-originated nitrates/nitrites. Of note, processed meat for instance might contain both foods-originated nitrites and additives-originated nitrites, hence its presence in the 2 categories, as explained in response to Reviewer #3, point 4.

Furthermore, we have now performed additional analyses as also advised by the Academic Editor, point 2, looking into associations between type-2 diabetes risk and nitrites/nitrates from different sources (including fruits and vegetables):

*Statistically signification associations were observed for nitrites from red and processed meats (HR_tertile 3 vs.1_= 1.30 (95% CI 1.30-1.58), P-trend=0.01) and nitrates from red and processed meats (HR_tertile 3 vs.1_= 1.34 (95% CI 1.10-1.63), P-trend=0.03), but not for nitrites from fruits and vegetables (HR_tertile 3 vs.1_= 1.10 (95% CI 0.88-1.37), P-trend=0.4) or nitrates from fruits and vegetables (HR_tertile 3 vs.1_= 0.92 (95% CI 0.74-1.14), P-trend=0.5).*

3.	Two validation studies are referenced, but they do not validate the assessment of nitrate/nitrite, which may be much more challenging given the different sources.

+++ As explained in our answer to Reviewer #1, point 1:

*“The accuracy of the qualitative and quantitative food additives exposure assessment of the NutriNet-Santé cohort represents an important strength of the study, thanks to detailed and repeated 24h-dietary records, links to multiple food composition databases (OQALI* [11]*, Open Food Facts* [12]*, GNPD* [13]*, EFSA* [14]*, and GSFA* [15]*), ad-hoc laboratory assays, and dynamic matching to account for reformulations of industrial food items over time* [16]*. We fully agree with Reviewer #1 that validation of additive exposure versus plasmatic or urinary biomarkers would be ideal, however, it is not possible for many food additives, among which nitrites and nitrates.*

*Indeed, specific validation of nitrites/nitrates intakes versus biomarkers has been envisioned at the beginning of the ADDITIVES ERC Program, but after careful examination of biomarker literature and scientific discussions among partners, it has been judged not feasible. Indeed, as for many food additives, it was not possible to identify markers in blood or urine that would be specific enough to reflect exogenous dietary exposure and not endogenous metabolism. More specifically, for instance, plasma nitrate would not only be a suitable marker for dietary sources, but would also reflect nitrate derived from various individual (patho‐) physiological pathways, as well as the individual capacity to eliminate nitrate* [17–19]*. Similarly for urinary nitrites, in addition to the fact that urinary tract infections are an important driver of urine nitrites levels* [20]*, therefore providing biased estimates of dietary exposure.”*

*We have added these elements to the discussion page 20:*

*‘Lastly, exposure to nitrates and nitrates has not been validated with blood or urine assays due to lack of biomarkers that would be specific enough to reflect exogenous dietary exposure and not endogenous metabolism. For instance, plasma nitrate would not only be a suitable marker for dietary sources, but would also reflect nitrate derived from various individual (patho‐) physiological pathways, as well as the individual capacity to eliminate nitrate* [17–19]*. Similarly for urinary nitrites, in addition to the fact that urinary tract infections are an important driver of urine nitrites levels* [20]*, therefore providing biased estimates of dietary exposure.’ ”*

4.	Natural sources should be defined in the abstract. Line 54 of the abstract should specify the sources evaluated instead of saying “any” source.

+++ The term “natural sources” is no longer used (see above). Besides, we have now rephrased the sentence as requested, page 2:

*“**There was no evidence for an association between total, foods and water-originated, or additives-originated nitrates and T2D risk (all P-trend>0.4).”*

5.	The authors mention soil contamination in the Author Summary, but do not discuss this anywhere else. If this is important, please elaborate in the manuscript.

+++ The use of nitrogen fertilizers during agricultural practices is responsible for groundwater and surface water pollution by nitrates as well as soil health degradation. We have now added sentences in the introduction regarding soil and water contamination by nitrates.

*“[…] as they are naturally present in water and soil (1).* *The use of nitrogen fertilizers during agricultural practices is responsible for groundwater and surface water pollution by nitrates as well as soil health degradation, which is a rising problem for both human health and ecosystems* [40]*.”*

6.	The water nitrate and nitrite were assessed from the records of tap-water levels based on the participants’ residence location. This is a limitation as people may consume varying amounts of tap-water, and some may spend substantial or most time outside their residence. The nitrate and nitrite in the water in their location may not adequately reflect individual consumption. This limitation needs to be discussed.

+++ We have added some precision regarding estimation of nitrites/nitrates from water in our response to Reviewer #2, point 7:

*‘The French official regional sanitary control of tap water was used to estimate intakes via water consumption, by region of residence* [34]*: SISE-Eaux is a French governmental database including data on quality control of tap water in the 34,955 French municipalities. It is coordinated by the Regional Health Agencies and consists of a collection of 310,000 samples per year (12 million samples overall). Data is available publicly* [34] *and a municipality-specific merge according to the NutriNet-Santé participants’ postal code has been performed. A dynamic temporal merge according to the year of dietary records was also considered.’*

Besides we added some discussion about this point page 20: *“**Another limitation is possible classification bias in the estimation of water-originated exposure to nitrites/nitrates based on regional and temporal information about tap water contamination, since some individuals may have spent time outside their region of residence. However, French national statistics show that the average French person spends less than a month (27.9 days) outside their residence* [41]*. Moreover, water-originated nitrites and nitrates represent a small minority of foods and water-originated nitrites and nitrates intakes (<0.01% and 6.9%, respectively). This potential bias was therefore not likely to have a significant effect on our estimates.”*

7.	This is a low-risk group with high socioeconomic status and healthier diets. The related concerns about generalizability need to be directly mentioned.

+++ As explained in our response to Reviewer #2, point 1:

*+++ One of the main objectives of the NutriNet-Santé cohort is to investigate the relationship between diet and health. NutriNet-Santé is a web-based cohort, and participants are recruited on a voluntary basis. Therefore, NutriNet-Santé does not aim to be representative of the general French population, but instead to have enough contrast between different categories of dietary exposures to conduct etiological analyses, while accounting for a wide diversity of lifestyle profiles. As it is usually the case in population-based cohorts, participants in NutriNet-Santé were younger, more often women, and had higher socio-professional and educational levels than the general (French) population* [21]*. NutriNet-Santé shows a good representativeness of adults aged below 75 years old, as this population has mostly home access to the Internet* [22]*, and underrepresents participants above 75 years old, who have less access to regular home Internet connection. Therefore, caution is needed when extrapolating results from this cohort to participants aged above 75 years old. Nevertheless, the NutriNet-Santé cohort study provides an access to a broad diversity of populations, including disadvantaged socio-professional categories (for instance, the proportion of unemployed participants (6.4%) is lower than that of the general population (10%)* [21]*, but is higher than what is obtained in most population-based studies using traditional methods of recruitment). NutriNet-Santé’s participants were less likely to smoke* [23]*, and to be overweight or obese (28.2% of men and 29.4% of women in NutriNet-Santé versus 54% and 44% in the French population* [23]*). In addition, participants in the NutriNet-Santé cohort also had in average healthier dietary habits than the French population (reporting higher intakes of fruit, vegetables, and fish, and lower intakes of red meat and added fats)* [24]*. These aforementioned factors explain the lower T2D incidence in our cohort (179 cases per 100,000 person-years after age and sex standardization) in comparison with the general French population (289 cases per 100,000 person-years* [25]*). This overall healthier cohort profile is not specific to the NutriNet-Santé study but generally observed in other well-known large-scale cohorts (e.g. Nurses Health study of Health Professional Follow-up study in the USA, Epic cohort in Europe, etc.). Still, a substantial proportion of participants developed T2D during follow-up and a substantial proportion consumed important amounts of processed meat, nitrites, nitrates, etc. (not all participants had a healthy diet)…, thereby allowing us to perform inter-group comparisons. We have added some discussion about this point in the discussion section page 18:*

*‘Second, as in every population-based observational cohort, generalizability of the results to the whole population should be discussed. Compared with the general French population, NutriNet-Santé participants were younger, more often women, with higher educational and socio-professional levels* [21]*. NutriNet-Santé shows a good representativeness of adults aged below 75 years old, as this population has mostly home access to the Internet* [22]*, and underrepresents participants above 75 years old, who have less access to regular home Internet connection. Therefore, caution is needed when extrapolating results from this cohort to participants aged above 75 years old. Nevertheless, the NutriNet-Santé cohort study provides an access to a broad diversity of populations, including disadvantaged socio-professional categories (for instance, the proportion of unemployed participants (6.4%) is lower than that of the general population (10%)* [21]*, but is higher than what is obtained in most population-based studies using traditional methods of recruitment). NutriNet-Santé’s participants were less likely to smoke* [23]*, and to be overweight or obese (28.2% of men and 29.4% of women in NutriNet-Santé versus 54% and 44% in French population* [23]*). These aforementioned factors explain the lower T2D incidence in our cohort (179 cases per 100,000 person-years after age and sex standardization) in comparison with the general French population (289 cases per 100,000 person-years* [25]*). Participants had also healthier dietary habits* [24]*, as they had higher consumption levels of fruit and vegetables and lower levels of processed meats consumption compared to the general population, which led to higher levels of exposure to total nitrites and nitrates and lower levels of exposure to food additives in NutriNet-Santé, compared with nationally representative surveys: indeed, exposure levels in the French INCA2 nationally representative survey were 0.04 mg/kg of body weight (BW) for total nitrites* [26] *and 1.6 mg/kg BW for total nitrates* [27]*, versus 0.09 and 3.34 mg/kg BW in NutriNet-Santé, respectively. Similarly, average intakes of nitrates in our study were higher than those reported in a systematic review by Babateen et al.* [28]*: average intake of total nitrates in healthy European populations = 107.21 mg/d vs. 213.2 mg/d in our study. In contrast, median intakes of nitrites and nitrates in an Iranian study were higher than those in our study (410 mg/d vs. 194 mg/d in our study for nitrates and 8.8 mg/d vs. 5.0 mg/d in our study for nitrites)* [29]*. As regards food additives specifically, exposure levels were 0.01 to 0.04 mg/kg BW for nitrites from additives and 0.05 to 0.10 mg/kg BW for nitrates from additives in the multi-country EFSA report, versus 0.004 mg/kg BW and 0.003 mg/kg BW in NutriNet-Santé, respectively. This might have led to an underestimation of true associations with nitrites and nitrates from food additives (due to the reduced contrast between compared groups). In contrast, regarding foods and water-originated nitrites and nitrates, the distribution of exposure in our population allowed us to have enough high consumers to properly explore this relatively extreme part of the curve, which exists (to a lesser extent) in the general population.’*

8.	The discussion focuses on nitrites, and does not go into details about the lack of association with nitrates. The literature suggests that beetroot and spinach may have a beneficial role in cardiometabolic conditions, partly due to their high nitrate content. The lack of association with nitrates and the implications for diet should be discussed.

We have now added a discussion (page 16) on the lack of association with nitrates:

*‘Green leafy vegetables are the major source of dietary nitrates. Even though their role in reducing T2D risk has been suggested* [1,2]*, the most recent meta-analyses* [3–5] *have reported low to very low quality of evidence for the association of incidence of cruciferous and green leafy vegetables with T2D risk. This is in line with our findings regarding the lack of association between foods-originated nitrates with T2D risk, or fruits and vegetables-originated nitrites and nitrates with T2D risk. In any case, even though the evidence from our study for these specific analyses might be relatively weak given the 1) moderate-to-strong correlations between nitrites and nitrates from fruits and vegetables with their other components (e.g., vitamin C [Pearson correlation coefficients = 0.41 for nitrites and 0.31 for nitrates], beta-carotene [Pearson correlation coefficients = 0.50 for nitrites and 0.58 for nitrates], dietary fibers [Pearson correlation coefficients = 0.63 for nitrites and 0.57 for nitrates]), 2) the possible lack of statistical power, and 3) potential residual confounding or classification biases, vegetables remain important sources of fibers and antioxidants, and are beneficial components of a healthy diet playing an important role in the prevention of chronic diseases such as cardiovascular diseases and some cancers.’*

9.	Importantly, the potential beneficial role of nitrates may be countered by mouthwash use, which may be high in this high income population. Most over-the-counter mouthwash have antibacterial properties which impact nitrate reduction in the mouth by oral bacteria, and impact nitric oxide metabolism. Recent studies have shown that regular over-the-counter mouthwash use is associated with increased risk of diabetes. Hence, the null results may be partly attributed to mouthwash use. If data is available, the authors should control for mouthwash use, and also presents analyses stratified by mouthwash use. If data is not available to control/stratify on mouthwash use, this should be discussed as a major limitation in the abstract, discussion and conclusion, as this may explain the null association with nitrates.

+++ We thank Reviewer #4 for this original and interesting hypothesis. We read with great interest the longitudinal study conducted in Puerto-Rico exploring associations between regular ruse of mouthwash and risk of prediabetes/diabetes. Data about mouthwash is available only for a subsample of participants (n=25,328) through an ad-hoc questionnaire administered in 2021. We have performed sensitivity analyses by adjusting or stratifying for mouthwash use. We observed no associations in this sample between nitrates (whatever the source) and R2D risk after adjusting (all p>0.2) or stratifying (all P_-interaction_ >0.4) for mouthwash use.

We have now added these results to Table E in S1-Appendix and added some discussion:

*“Furthermore, a recent study suggested positive associations between regular mouthwash use and type-2 diabetes through an anti-bacterial oral effect that might stop the reduction of nitrates to nitrites in the oral cavity* [42]*. Our results showed no association with nitrates, independently of regular mouthwash use. However, data on mouthwash use was available only in a small subgroup of participants; therefore, we might have lacked statistical power to accurately investigate this assumption. In addition, only 15% of the sample reported using mouthwash at least once a week.”*

10.	Appendix 7 should show effect estimates, not just p-values. Also, it would be helpful to discuss differences in effect estimates across the antioxidant subgroups, as interaction tests often do not have enough power.

+++ We have now presented the full results for stratified analyses by antioxidant intakes in Table C in S1-Appendix. In stratified models, associations between nitrites and T2D risk appeared to be more pronounced in participants with vitamin C intake ≥ median compared to those with lower intakes, in contrast with the study by Bahadoran [29] finding associations only in the bottom subgroup (these results should be interpreted with caution since interaction tests were not significant). Several hypotheses could explain these results in our population: first, fruit and vegetables are an important source of both nitrites and vitamin C. Therefore, individuals in the top median of vitamin C intakes are also those with higher values of nitrites intake (mean food and water-originated nitrite intake = 5.8 mg/d versus 4.9 mg/d in participants with vitamin C intake below the median), thus, we were more inclined to detect associations between nitrite intake and T2D risk in this subgroup. Second, participants with higher antioxidant intakes were older (43.6 years in the top vitamin C category vs. 41.7 in the bottom category) and had higher energy intakes (1,934 Kcal/day in the top vitamin C category vs. 1,757 in the bottom category), which has led to a higher number of cases and a stronger statistical power in participants with higher antioxidant intakes. We have added some discussion about this point page 16.

11.	Appendix 9 title mentions “Cause-specific…” This should be reworded, as one cannot interpret causality from this study. Also, the manuscript erroneously refers to Appendix 9 as Appendix 8.

+++ In fact, “cause-specific Cox models” is the appropriate technical term for the method used to handle competing risks [43]. This terminology is independent from the design of the study and does not imply causality. Of course, we fully agree that one cannot conclude in a causal link from this type of (observational) study.

We have clarified this point in the Methods page 10: “*Cause-specific Cox models were applied, with death considered as a competing event.* *In observational cohort studies, these models do not allow to interpret data in terms of direct causal inference but are designed to account for competing risks* [43]*.*”

Besides, we have now renumbered the Appendices accordingly, we apologize for this typo.

Reviewer #5: Since the health effect of nitrites and nitrates on incident type-2 diabetes (T2D) is inconclusive and epidemiological and clinical evidence are lacking, this study aimed to examine the association between nitrites/nitrates and T2D through a population based prospective cohort study. The study also distinguished the effects of natural and food additive sources of nitrites and nitrates on T2D. Overall, I think this is a carefully designed and conducted cohort study and the research topic is of importance to population health. However, I am having a little concern on the web-based cohort and the self-administered questionnaire as I think the cohort may not be well representative of the population. Particularly, I think the study population under-represents the old people and people with low or no education. Below please find my specific comments.

+++ We thank Reviewer #5 for his/her positive comment. Please find our responses below.

1. Competing Interests: please make sure that the funding from the IFIP does not have conflict of interest with this study.

+++ The funding from IFIP was only granted to Dr. Fabrice Pierre (at Inrae Toxalim), who is not part of the research laboratory that conducted the work (EREN lab). This specific funding concerned another experimental research project on processed meats and colorectal cancer. It aimed at evaluating technical solutions (e.g. agricultural practices, formulation) to mitigate the well-established deleterious impact of processed meat on colorectal cancer risk. It is not related at all to the present project/manuscript nor to type-2 diabetes. IFIP had no role in designing the present study nor funding it. The results of this study have never been communicated to IFIP. Dr Pierre only mentioned this link for pure transparency, but it does not constitute a conflict of interest for the present work.

The EREN laboratory, coordinator of the present project, is only funded by public and non-profit associative grants.

Besides, our results provide additional evidence for health risks (here, T2D) of processed meat (and the nitrites they contain), which goes against the economic interests of IFIP.

2. Data Availability: noted that data are not fully publicly available.

+++ Data from the NutriNet-Santé cohort is not publicly available online in an open repository, as this would not be allowed by French regulation for sensitive personal data protection and as this was not part of the consent collected from participants at the moment of the cohort’s design and launching in 2009. However, as explained in the “Data availability” section page 26, researchers from public institutions can submit a collaboration request including information on the institution and a brief description of the project to collaboration@etude-nutrinet-sante.fr. All requests will be reviewed by the steering committee of the NutriNet-Santé study. If the collaboration request is accepted, a data access agreement will be necessary and appropriate authorizations from the competent administrative authorities may be needed. In accordance with existing regulations, no personal identification data (name, address, etc.) will be accessible.

3. Line 139-140: given that the cohort is web-based and only participants with access to internet are recruited, is there any concern regarding the representativeness of the cohort. Since young people tend to use internet more than the old people, is there skewed age distribution in the recruited cohort?

4. Line 156-158: since the surveys are all self-administered, I am worried whether the cohort will be representative of people with low or no education? Also, is there any concern on the quality of the self-administered survey?

Response to 3 and 4:

+++ We have now provided age distribution in Figure B in S1-Appendix. Our sample shows a good representativeness of adults below 70 years old. French national statistics show that between 61.9 et 96.2 % of adults below 75 years old have home Internet access, compared with 23.4% in participants aged above 75 years old [22]. This would lead to an underrepresentation of older adults (above 75 years old). Mean age at T2D diagnosis in France is 65 years; our cohort was therefore adapted to study this outcome, even though we might have underestimated the incidence compared to the general population. As regards representativeness, and as explained in our answer to Reviewer #2, point 1:

*+++ One of the main objectives of the NutriNet-Santé cohort is to investigate the relationship between diet and health. NutriNet-Santé is a web-based cohort, and participants are recruited on a voluntary basis. Therefore, NutriNet-Santé does not aim to be representative of the general French population, but instead to have enough contrast between different categories of dietary exposures to conduct etiological analyses, while accounting for a wide diversity of lifestyle profiles. As it is usually the case in population-based cohorts, participants in NutriNet-Santé were younger, more often women, and had higher socio-professional and educational levels than the general (French) population* [21]*. NutriNet-Santé shows a good representativeness of adults aged below 75 years old, as this population has mostly home access to the Internet* [22]*, and underrepresents participants above 75 years old, who have less access to regular home Internet connection. Therefore, caution is needed when extrapolating results from this cohort to participants aged above 75 years old. Nevertheless, the NutriNet-Santé cohort study provides an access to a broad diversity of populations, including disadvantaged socio-professional categories (for instance, the proportion of unemployed participants (6.4%) is lower than that of the general population (10%)* [21]*, but is higher than what is obtained in most population-based studies using traditional methods of recruitment). NutriNet-Santé’s participants were less likely to smoke* [23]*, and to be overweight or obese (28.2% of men and 29.4% of women in NutriNet-Santé versus 54% and 44% in the French population* [23]*). In addition, participants in the NutriNet-Santé cohort also had in average healthier dietary habits than the French population (reporting higher intakes of fruit, vegetables, and fish, and lower intakes of red meat and added fats)* [24]*. These aforementioned factors explain the lower T2D incidence in our cohort (179 cases per 100,000 person-years after age and sex standardization) in comparison with the general French population (289 cases per 100,000 person-years* [25]*). This overall healthier cohort profile is not specific to the NutriNet-Santé study but generally observed in other well-known large-scale cohorts (e.g. Nurses Health study of Health Professional Follow-up study in the USA, Epic cohort in Europe, etc.). Still, a substantial proportion of participants developed T2D during follow-up and a substantial proportion consumed important amounts of processed meat, nitrites, nitrates, etc. (not all participants had a healthy diet)…, thereby allowing us to perform inter-group comparisons. We have added some discussion about this point in the discussion section page 18:*

*‘Second, as in every population-based observational cohort, generalizability of the results to the whole population should be discussed. Compared with the general French population, NutriNet-Santé participants were younger, more often women, with higher educational and socio-professional levels* [21]*. NutriNet-Santé shows a good representativeness of adults aged below 75 years old, as this population has mostly home access to the Internet* [22]*, and underrepresents participants above 75 years old, who have less access to regular home Internet connection. Therefore, caution is needed when extrapolating results from this cohort to participants aged above 75 years old. Nevertheless, the NutriNet-Santé cohort study provides an access to a broad diversity of populations, including disadvantaged socio-professional categories (for instance, the proportion of unemployed participants (6.4%) is lower than that of the general population (10%)* [21]*, but is higher than what is obtained in most population-based studies using traditional methods of recruitment). NutriNet-Santé’s participants were less likely to smoke* [23]*, and to be overweight or obese (28.2% of men and 29.4% of women in NutriNet-Santé versus 54% and 44% in French population* [23]*). These aforementioned factors explain the lower T2D incidence in our cohort (179 cases per 100,000 person-years after age and sex standardization) in comparison with the general French population (289 cases per 100,000 person-years* [25]*). Participants had also healthier dietary habits* [24]*, as they had higher consumption levels of fruit and vegetables and lower levels of processed meats consumption compared to the general population, which led to higher levels of exposure to total nitrites and nitrates and lower levels of exposure to food additives in NutriNet-Santé, compared with nationally representative surveys: indeed, exposure levels in the French INCA2 nationally representative survey were 0.04 mg/kg of body weight (BW) for total nitrites* [26] *and 1.6 mg/kg BW for total nitrates* [27]*, versus 0.09 and 3.34 mg/kg BW in NutriNet-Santé, respectively. Similarly, average intakes of nitrates in our study were higher than those reported in a systematic review by Babateen et al.* [28]*: average intake of total nitrates in healthy European populations = 107.21 mg/d vs. 213.2 mg/d in our study. In contrast, median intakes of nitrites and nitrates in an Iranian study were higher than those in our study (410 mg/d vs. 194 mg/d in our study for nitrates and 8.8 mg/d vs. 5.0 mg/d in our study for nitrites)* [29]*. As regards food additives specifically, exposure levels were 0.01 to 0.04 mg/kg BW for nitrites from additives and 0.05 to 0.10 mg/kg BW for nitrates from additives in the multi-country EFSA report, versus 0.004 mg/kg BW and 0.003 mg/kg BW in NutriNet-Santé, respectively. This might have led to an underestimation of true associations with nitrites and nitrates from food additives (due to the reduced contrast between compared groups). In contrast, regarding foods and water-originated nitrites and nitrates, the distribution of exposure in our population allowed us to have enough high consumers to properly explore this relatively extreme part of the curve, which exists (to a lesser extent) in the general population.’*

As regards the self-administered questionnaires, our team is pioneer in e-epidemiology tools and NutriNet-Santé was the largescale first e-cohort in the world investigating the relationships between nutrition and health, and its architecture is currently used by several other e-cohorts worldwide.

Several validation studies have been performed. As described in our response to Reviewer #1, point 1:

*‘Prior to the launching of the NutriNet-Santé cohort in 2008-2009, two studies have been performed to validate the web-based dietary records against biomarkers: one against plasma biomarkers* [8] *suggested that this tool provides reasonable estimates of true intake of fruits, vegetables, fish, beta carotene, vitamin C, and n-3 fatty acids. The other one against urinary biomarkers* [9] *showed that these web-based dietary records appear to be valid for estimating usual intakes of proteins and potassium. Besides, the self-administered web-based dietary record tool has also been validated against an interview by a trained dietician showing high correlations for all food groups, energy, macro and micro-nutrients* [10]*.’*

In addition, it has been demonstrated that Internet use reduces social desirability bias. Indeed, people being less exposed to direct judgment by an interviewer tend to more and better declare sensitive information such as ‘at risk’ practices (consumption of “unhealthy” fatty and sweet foods, alcohol use, tobacco smoke) but also their social status (unemployment, income) [44,45].

The self-administered socio-demographic and anthropometric questionnaires were also validated against paper questionnaires and an interview with a dietitian [10,46], which has showed the advantage of online web-questionnaires in increasing the quality of collected data and reducing input error and outliers thanks to integrated field controls. Anthropometric self-reported data were also validated against clinical measures [47].

All these validation studies ensured that collected data has a high reliable quality, that has been used in the framework of several high quality epidemiological studies [48–50].

5. Line 195-198: I don't feel comfortable to ascertain people's T2D status through reimbursement of medication. First of all, if people do not want to disclose their disease status, is it ethical to track their medication reimbursement records to expose their disease status? Second, I am hesitant whether this can accurately ascertain people's T2D onset.

+++ In France, our research team was the first to obtain the authorization by decree in the Council of State (n°12013236) to link data from a population-based cohort to external medico-administrative databases of the national health insurance. Now, the system is been generalized to other cohorts, provided compliance with procedures that ensure strict respect of confidentiality and individual rights. Participants have been informed about this linkage and have the right to revoke their given consent at any time on a dedicated interface on the study website. We have added this precision in Method C in S1-Appendix.

*Besides, as regards case ascertainment, please refer to our response to Reviewer #1 point 2:*

*‘T2D case ascertainment was based on self-report, but was confirmed using a multi-source strategy. Reporting both incident T2D and the use of T2D medication via the baseline and health follow-up questionnaire (administered every 3 months) or the dedicated health events platform, was mandatory for the case to be considered as a confirmed T2D case. The questions were: “Have you been diagnosed with T2D (if yes, indicate the date of diagnosis)” and “Are you treated for T2D ?”. Participants are asked to record all currently taken medications (T2D medications and others). A search engine with embedded exhaustive Vidal® drug database is used to facilitate medication data entry for the participants. ATC codes considered for T2D medication were A10AB01, A10AB03, A10AB04, A10AB05, A10AB06, A10AC01, A10AC03, A10AC04, A10AD01, A10AD03, A10AD04, A10AD05, A10AE01, A10AE02, A10AE03, A10AE04, A10AE05, A10AE30, A10BA02, A10BB01, A10BB03, A10BB04, A10BB06, A10BB07, A10BB09, A10BB12, A10BD02, A10BD03, A10BD05, A10BD07, A10BD08, A10BD10, A10BD15, A10BD16, A10BF01, A10BF02, A10BG02, A10BG03, A10BH01, A10BH02, A10BH03, A10BX02, A10BX04, A10BX07, A10BX09, A10BX10, A10BX11, A10BX12.*

*Following a T2D diagnosis and/or medication declaration, two additional sources of information were considered for confirmation. First, the linkage with the SNIIRAM National health insurance database allowed confirming 85.7% of investigated cases (ICD-10 codes E11). The sensitivity of SNIIRAM databases is not optimal if used without self-report data: of note, about 10-15% of the French population is covered by other social security regimen and would not be correctly captured by the SNIIRAM databases. Besides, the centralization of SNIIRAM data might take up to a year, leading to delays between reported T2D information and health insurance data.*

*Second, as regards biological samples, a subsample of participants (n=19,800) were invited to a clinical examination, during which they provided blood and urine samples. We have added details about biological assessments in the Methods. Among the participants who provided blood sample during the clinical/biological examination, 232 had elevated fasting blood glucose (i.e. >1.26 g/L). Among them, 85.3% had consistently declared a T2D diagnosis and/or medication. Elevated blood glucose alone (i.e., without any declaration of T2D diagnosis or treatment) was not considered specific enough to classify the participant as a T2D case.’*

*Although under-detection of some diagnosed T2D cases is still possible at baseline and during follow-up, it is likely to have been limited thanks to this multi-source strategy for case ascertainment (combining self-report of disease, self-report of medication, link to national health insurance database, and fasting blood glucose on a subsample). The fact that exhaustiveness of T2D case detection cannot be guaranteed (especially those that have never been diagnosed or treated) would mainly have led to reduced statistical power and non-differential classification errors and thus likely to an underestimation of the observed associations. In contrast, it is not likely to explain the statistically significant associations observed. This point has been added in the discussion section page 20.’*

6. Line 222-224: when studying sodium nitrite, will adjusting for potassium nitrite (e249), natural nitrite, and overall nitrate intakes blind/obscure the effects of sodium nitrite?

+++ Mutual adjustment for other types or sources of nitrites/nitrates allows to focus on the additive that is investigated and its potentially specific and maybe differential association with T2D, avoiding/limiting confounding by other types or sources. This strategy was also recommended by the Academic Editor.

Any attachments provided with reviews can be seen via the following link:

<https://www.editorialmanager.com/pmedicine/l.asp?i=803522&l=85AXTB0C>

**References**

1.	Carter P, Gray LJ, Troughton J, Khunti K, Davies MJ. Fruit and vegetable intake and incidence of type 2 diabetes mellitus: systematic review and meta-analysis. BMJ. 2010;341: c4229. doi:10.1136/bmj.c4229

2.	Li M, Fan Y, Zhang X, Hou W, Tang Z. Fruit and vegetable intake and risk of type 2 diabetes mellitus: meta-analysis of prospective cohort studies. BMJ Open. 2014;4: e005497. doi:10.1136/bmjopen-2014-005497

3.	Schwingshackl L, Hoffmann G, Lampousi A-M, Knüppel S, Iqbal K, Schwedhelm C, et al. Food groups and risk of type 2 diabetes mellitus: a systematic review and meta-analysis of prospective studies. Eur J Epidemiol. 2017;32: 363–375. doi:10.1007/s10654-017-0246-y

4.	Neuenschwander M, Ballon A, Weber KS, Norat T, Aune D, Schwingshackl L, et al. Role of diet in type 2 diabetes incidence: umbrella review of meta-analyses of prospective observational studies. BMJ. 2019;366: l2368. doi:10.1136/bmj.l2368

5.	Chen G-C, Koh W-P, Yuan J-M, Qin L-Q, Dam RM van. Green leafy and cruciferous vegetable consumption and risk of type 2 diabetes: results from the Singapore Chinese Health Study and meta-analysis. British Journal of Nutrition. 2018;119: 1057–1067. doi:10.1017/S0007114518000119

6.	Micha R, Wallace SK, Mozaffarian D. Red and Processed Meat Consumption and Risk of Incident Coronary Heart Disease, Stroke, and Diabetes Mellitus. Circulation. 2010;121: 2271–2283. doi:10.1161/CIRCULATIONAHA.109.924977

7.	Hercberg S, Galan P, Touvier M, Méjean C, Kesse E, Fezeu LK, et al. The Nutrinet-Santé study: a web-based prospective cohort study of the relationship between nutrition and health and of dietary patterns and nutritional status predictors. Paris: Nutritional Epidemiology Research Unit INSERM U557/INRA 1125/CBAM/University of Paris 13; 2009 p. 38. Available: https://info.etude-nutrinet-sante.fr/upload/siteinfo/files/ck/files/protocole%20general%20en%20anglais%20NutriNet_Aug2013_EM_03_09_13.pdf

8.	Lassale C, Castetbon K, Laporte F, Deschamps V, Vernay M, Camilleri GM, et al. Correlations between Fruit, Vegetables, Fish, Vitamins, and Fatty Acids Estimated by Web-Based Nonconsecutive Dietary Records and Respective Biomarkers of Nutritional Status. JAcadNutr Diet. 2016;116: 427–438. doi:10.1016/j.jand.2015.09.017

9.	Lassale C, Castetbon K, Laporte F, Camilleri GM, Deschamps V, Vernay M, et al. Validation of a Web-based, self-administered, non-consecutive-day dietary record tool against urinary biomarkers. BrJNutr. 2015;113: 953–962. doi:10.1017/S0007114515000057

10.	Touvier M, Kesse-Guyot E, Mejean C, Pollet C, Malon A, Castetbon K, et al. Comparison between an interactive web-based self-administered 24 h dietary record and an interview by a dietitian for large-scale epidemiological studies. BrJNutr. 2011;105: 1055–1064. doi:10.1017/S0007114510004617

11.	Observatoire de l’alimentation (Oqali). OQALI - Home page. [cited 22 Oct 2020]. Available: https://www.oqali.fr/oqali_eng/

12.	Open Food Facts. [cited 22 Oct 2020]. Available: https://world.openfoodfacts.org/discover

13.	Global New Products Database (GNPD). Banque de données mondiale de nouveaux produits, suivi des tendances nouveaux produits et innovations. [cited 22 Oct 2020]. Available: https://www.gnpd.com/sinatra/anonymous_frontpage/

14.	EFSA ANS Panel. (EFSA Panel on Food Additives and Nutrient Sources added to Food) - Scientific Opinion on the re-evaluation of aspartame (E 951) as a food additive. EFSA Journal 2013;11(12):3496, 263 pp. doi:10.2903/j.efsa.2013.3496. 2013.

15.	Food and Agriculture Organization/World Health Organization (FAO/WHO). Codex General Standard for Food Additives (GSFA, Codex STAN 192-1995). Codex Alimentarius Commission; 2019. Available: http://www.fao.org/fao-who-codexalimentarius/sh-proxy/en/?lnk=1&url=https%253A%252F%252Fworkspace.fao.org%252Fsites%252Fcodex%252FStandards%252FCODEX%2BSTAN%2B192-1995%252FCXS_192e.pdf

16.	Chazelas E, Druesne-Pecollo N, Esseddik Y, Szabo de Edelenyi F, Agaësse C, De Sa A, et al. Exposure to food additive mixtures in 106,000 French adults from the NutriNet-Santé cohort. Sci rep. 2021;11: 19680. doi:10.1038/s41598-021-98496-6

17.	Yoon Y, Song J, Hong SH o, Kim JQ. Plasma Nitric Oxide Concentrations and Nitric Oxide Synthase Gene Polymorphisms in Coronary Artery Disease. Clinical Chemistry. 2000;46: 1626–1630. doi:10.1093/clinchem/46.10.1626

18.	Baylis C, Vallance P. Measurement of nitrite and nitrate levels in plasma and urine — what does this measure tell us about the activity of the endogenous nitric oxite system? Current Opinion in Nephrology and Hypertension. 1998;7: 59–62.

19.	Minamino T, Kitakaze M, Sato H, Asanuma H, Funaya H, Koretsune Y, et al. Plasma Levels of Nitrite/Nitrate and Platelet cGMP Levels Are Decreased in Patients With Atrial Fibrillation. Arteriosclerosis, Thrombosis, and Vascular Biology. 1997;17: 3191–3195. doi:10.1161/01.ATV.17.11.3191

20.	Nitrites in Urine: MedlinePlus Medical Test. [cited 17 Aug 2022]. Available: https://medlineplus.gov/lab-tests/nitrites-in-urine/

21.	Andreeva VA, Salanave B, Castetbon K, Deschamps V, Vernay M, Kesse-Guyot E, et al. Comparison of the sociodemographic characteristics of the large NutriNet-Sante e-cohort with French Census data: the issue of volunteer bias revisited. JEpidemiolCommunity Health. 2015;69: 893–898. doi:10.1136/jech-2014-205263

22.	L’internet de plus en plus prisé, l’internaute de plus en plus mobile - Insee Première - 1452. [cited 2 Sep 2022]. Available: https://www.insee.fr/fr/statistiques/1281312

23.	Santé Publique France. ESTEBAN - A health study on environment, biosurveillance, physical activity and nutrition 2014-2016 (Original title in French: Etude de santé sur l’environnement, la biosurveillance, l’activité physique et la nutrition (Esteban). 2017 [cited 17 Oct 2018]. Available: http://invs.santepubliquefrance.fr/Publications-et-outils/Rapports-et-syntheses/Environnement-et-sante/2017/Etude-de-sante-sur-l-environnement-la-biosurveillance-l-activite-physique-et-la-nutrition-Esteban-2014-2016

24.	Andreeva VA, Deschamps V, Salanave B, Castetbon K, Verdot C, Kesse-Guyot E, et al. Comparison of Dietary Intakes Between a Large Online Cohort Study (Etude NutriNet-Santé) and a Nationally Representative Cross-Sectional Study (Etude Nationale Nutrition Santé) in France: Addressing the Issue of Generalizability in E-Epidemiology. Am J Epidemiol. 2016;184: 660–669. doi:10.1093/aje/kww016

25.	Fagot-Campagna A, Fosse S, Roudier C, Romon I. Prévalence et incidence du diabète, et mortalité liée au diabète en France. 2010 Nov. Available: http://invs.santepubliquefrance.fr/Publications-et-outils/Rapports-et-syntheses/Maladies-chroniques-et-traumatismes/2010/Prevalence-et-incidence-du-diabete-et-mortalite-liee-au-diabete-en-France

26.	Mortensen A, Aguilar F, Crebelli R, Domenico AD, Dusemund B, Frutos MJ, et al. Re-evaluation of potassium nitrite (E 249) and sodium nitrite (E 250) as food additives. EFSA Journal. 2017;15: e04786. doi:10.2903/j.efsa.2017.4786

27.	Mortensen A, Aguilar F, Crebelli R, Domenico AD, Dusemund B, Frutos MJ, et al. Re-evaluation of sodium nitrate (E 251) and potassium nitrate (E 252) as food additives. EFSA Journal. 2017;15: e04787. doi:10.2903/j.efsa.2017.4787

28.	Babateen AM, Fornelli G, Donini LM, Mathers JC, Siervo M. Assessment of dietary nitrate intake in humans: a systematic review. The American Journal of Clinical Nutrition. 2018;108: 878–888. doi:10.1093/ajcn/nqy108

29.	Bahadoran Z, Mirmiran P, Ghasemi A, Carlström M, Azizi F, Hadaegh F. Vitamin C intake modify the impact of dietary nitrite on the incidence of type 2 diabetes: A 6-year follow-up in Tehran Lipid and Glucose Study. Nitric Oxide. 2017;62: 24–31. doi:10.1016/j.niox.2016.11.005

30.	Ferrari P, Slimani N, Ciampi A, Trichopoulou A, Naska A, Lauria C, et al. Evaluation of under- and overreporting of energy intake in the 24-hour diet recalls in the European Prospective Investigation into Cancer and Nutrition (EPIC). Public Health Nutrition. 2002;5: 1329–1345. doi:10.1079/PHN2002409

31.	Murakami K, Livingstone MBE. Prevalence and characteristics of misreporting of energy intake in US adults: NHANES 2003-2012. Br J Nutr. 2015;114: 1294–1303. doi:10.1017/S0007114515002706

32.	Markussen MS, Veierød MB, Ursin G, Andersen LF. The effect of under-reporting of energy intake on dietary patterns and on the associations between dietary patterns and self-reported chronic disease in women aged 50–69 years. British Journal of Nutrition. 2016;116: 547–558. doi:10.1017/S000711451600218X

33.	Anses. Etude Individuelle Nationale des Consommations Alimentaires 3 (INCA 3). 2017.

34.	Résultats du contrôle sanitaire de l’eau du robinet - data.gouv.fr. [cited 16 Feb 2021]. Available: /en/datasets/resultats-du-controle-sanitaire-de-leau-du-robinet/

35.	Lundberg JO, Carlström M, Weitzberg E. Metabolic Effects of Dietary Nitrate in Health and Disease. Cell Metab. 2018;28: 9–22. doi:10.1016/j.cmet.2018.06.007

36.	Ghasemi A. Quantitative aspects of nitric oxide production from nitrate and nitrite. EXCLI J. 2022;21: 470–486. doi:10.17179/excli2022-4727

37.	Willett W. Nutritional Epidemiology. Oxford University Press; 2012. doi:10.1093/acprof:oso/9780199754038.001.0001

38.	Bingham SA, Gill C, Welch A, Day K, Cassidy A, Khaw KT, et al. Comparison of dietary assessment methods in nutritional epidemiology: weighed records v. 24 h recalls, food-frequency questionnaires and estimated-diet records. British Journal of Nutrition. 1994;72: 619–643. doi:10.1079/BJN19940064

39.	Prentice RL, Mossavar-Rahmani Y, Huang Y, Van Horn L, Beresford SAA, Caan B, et al. Evaluation and Comparison of Food Records, Recalls, and Frequencies for Energy and Protein Assessment by Using Recovery Biomarkers. Am J Epidemiol. 2011;174: 591–603. doi:10.1093/aje/kwr140

40.	Mateo-Sagasta J, M Z S, H T. More people, more food, worse water? a global review of water pollution from agriculture. Rome, Italy: FAO Colombo, Sri Lanka: International Water Management Institute (IWMI). CGIAR Research Program on Water, Land and Ecosystems (WLE).; 2018.

41.	Enquête nationale transports et déplacements | Insee. [cited 2 Sep 2022]. Available: https://www.insee.fr/fr/metadonnees/source/serie/s1277

42.	Joshipura KJ, Muñoz-Torres FJ, Morou-Bermudez E, Patel RP. Over-the-counter mouthwash use and risk of pre-diabetes/diabetes. Nitric Oxide. 2017;71: 14–20. doi:10.1016/j.niox.2017.09.004

43.	Andersen PK, Geskus RB, de Witte T, Putter H. Competing risks in epidemiology: possibilities and pitfalls. International Journal of Epidemiology. 2012;41: 861–870. doi:10.1093/ije/dyr213

44.	Joinson A. Social desirability, anonymity, and Internet-based questionnaires. BehavResMethods InstrumComput. 1999;31: 433–438.

45.	Wang YC, Lee CM, Lew-Ting CY, Hsiao CK, Chen DR, Chen WJ. Survey of substance use among high school students in Taipei: web-based questionnaire versus paper-and-pencil questionnaire. JAdolescHealth. 2005;37: 289–295.

46.	Touvier M, Mejean C, Kesse-Guyot E, Pollet C, Malon A, Castetbon K, et al. Comparison between web-based and paper versions of a self-administered anthropometric questionnaire. EurJEpidemiol. 2010;25: 287–296. doi:10.1007/s10654-010-9433-9

47.	Lassale C, Péneau S, Touvier M, Julia C, Galan P, Hercberg S, et al. Validity of web-based self-reported weight and height: results of the Nutrinet-Santé study. J Med Internet Res. 2013;15: e152. doi:10.2196/jmir.2575

48.	Beslay M, Srour B, Méjean C, Allès B, Fiolet T, Debras C, et al. Ultra-processed food intake in association with BMI change and risk of overweight and obesity: A prospective analysis of the French NutriNet-Santé cohort. PLoS Med. 2020;17: e1003256. doi:10.1371/journal.pmed.1003256

49.	Chaltiel D, Julia C, Adjibade M, Touvier M, Hercberg S, Kesse-Guyot E. Adherence to the 2017 French dietary guidelines and adult weight gain: A cohort study. PLOS Medicine. 2019;16: e1003007. doi:10.1371/journal.pmed.1003007

50.	Debras C, Chazelas E, Srour B, Druesne-Pecollo N, Esseddik Y, Szabo de Edelenyi F, et al. Artificial sweeteners and cancer risk: Results from the NutriNet-Santé population-based cohort study. PLoS Med. 2022;19: e1003950. doi:10.1371/journal.pmed.1003950
