# Supplementary material for: Dietary exposure to nitrites and nitrates in association with type 2 diabetes risk: Results from the NutriNet-Santé population-based cohort study
Source: PLoS Med. 2023 Jan 17;20(1):e1004149. doi: 10.1371/journal.pmed.1004149 (PMC9844911; doi:10.1371/journal.pmed.1004149)
Supplement: Attachment — Submitted filename: response_r3_final.docx [file pmed.1004149.s005.docx]

CC: [cdavidson@plos.org](mailto:cdavidson@plos.org)

Dear Dr. Srour,

Thank you very much for re-submitting your manuscript "Dietary exposure to nitrites and nitrates in association with type-2 diabetes risk:  Results from the NutriNet-Santé population-based cohort study" (PMEDICINE-D-22-02217R2) for review by PLOS Medicine.

I have discussed the paper with my colleagues and the academic editor and it was also seen again by all reviewers. I am pleased to say that provided the remaining editorial and production issues are dealt with we are planning to accept the paper for publication in the journal.

The remaining issues that need to be addressed are listed at the end of this email. Any accompanying reviewer attachments can be seen via the link below. Please take these into account before resubmitting your manuscript:

***Please note while forming your response, if your article is accepted, you may have the opportunity to make the peer review history publicly available. The record will include editor decision letters (with reviews) and your responses to reviewer comments. If eligible, we will contact you to opt in or out.***

In revising the manuscript for further consideration here, please ensure you address the specific points made by each reviewer and the editors. In your rebuttal letter you should indicate your response to the reviewers' and editors' comments and the changes you have made in the manuscript. Please submit a clean version of the paper as the main article file. A version with changes marked must also be uploaded as a marked up manuscript file.

Please also check the guidelines for revised papers at <http://journals.plos.org/plosmedicine/s/revising-your-manuscript> for any that apply to your paper. If you haven't already, we ask that you provide a short, non-technical Author Summary of your research to make findings accessible to a wide audience that includes both scientists and non-scientists. The Author Summary should immediately follow the Abstract in your revised manuscript. This text is subject to editorial change and should be distinct from the scientific abstract.

We hope to receive your revised manuscript within 2 weeks. Please email us ([plosmedicine@plos.org](mailto:plosmedicine@plos.org)) if you have any questions or concerns.

We ask every co-author listed on the manuscript to fill in a contributing author statement. If any of the co-authors have not filled in the statement, we will remind them to do so when the paper is revised. If all statements are not completed in a timely fashion this could hold up the re-review process. Should there be a problem getting one of your co-authors to fill in a statement we will be in contact. YOU MUST NOT ADD OR REMOVE AUTHORS UNLESS YOU HAVE ALERTED THE EDITOR HANDLING THE MANUSCRIPT TO THE CHANGE AND THEY SPECIFICALLY HAVE AGREED TO IT.

Please ensure that the paper adheres to the PLOS Data Availability Policy (see <http://journals.plos.org/plosmedicine/s/data-availability>), which requires that all data underlying the study's findings be provided in a repository or as Supporting Information. For data residing with a third party, authors are required to provide instructions with contact information for obtaining the data. PLOS journals do not allow statements supported by "data not shown" or "unpublished results." For such statements, authors must provide supporting data or cite public sources that include it.

To enhance the reproducibility of your results, we recommend that you deposit your laboratory protocols in protocols.io, where a protocol can be assigned its own identifier (DOI) such that it can be cited independently in the future. Additionally, PLOS ONE offers an option to publish peer-reviewed clinical study protocols. Read more information on sharing protocols at <https://plos.org/protocols?utm_medium=editorial-email&utm_source=authorletters&utm_campaign=protocols>

Please review your reference list to ensure that it is complete and correct. If you have cited papers that have been retracted, please include the rationale for doing so in the manuscript text, or remove these references and replace them with relevant current references. Any changes to the reference list should be mentioned in the rebuttal letter that accompanies your revised manuscript.

Please note, when your manuscript is accepted, an uncorrected proof of your manuscript will be published online ahead of the final version, unless you've already opted out via the online submission form. If, for any reason, you do not want an earlier version of your manuscript published online or are unsure if you have already indicated as such, please let the journal staff know immediately at [plosmedicine@plos.org](mailto:plosmedicine@plos.org).

If you have any questions in the meantime, please contact me or the journal staff on [plosmedicine@plos.org](mailto:plosmedicine@plos.org).

We look forward to receiving the revised manuscript by Nov 18 2022 11:59PM.

Sincerely,

Callam Davidson,

Associate Editor

PLOS Medicine

plosmedicine.org

------------------------------------------------------------

Comments from the Academic Editor:

As the reviewers noted, one of the limitations in this study is no information on the validity of their exposure assessment (nitrates, nitrites).

The authors' approach to this limitation in writing is substandard. They argued as follows. First, it would be ideal to have objective biomarkers for exposures to nitrates and nitrites. Second, there is no promising biomarker for those.

These two arguments partly support why the authors could not assess the validity of their exposure assessment. However, the arguments do not mean we can ignore the specific limitation. The authors should provide an interpretation and conclusion with the weakness into consideration. In other words, the authors should consider the possibility that the finding was an artefact for example.

Any readers would understand that the authors did great for their exposure assessment, including using multiple databases and collecting detailed dietary data. However, the objective view on no information on the validity (sensitivity and specificity) is crucial.

+++ We thank the Academic Editor for these comments and advice. All of them have been accounted for, as detailed below. These edits improved the quality of our manuscript, and helped us address the study limitations.

In the revised document, the authors should do the followings:

The authors should revise the abstract and state that they gained no information on the validity and that measurement errors would be substantial to question the positive association. Of note, the error characteristics are unclear, and the authors should not consider the bias-toward-the-null argument as the direction is unclear overall.

+++ We have now included in the abstract a limitation about the lack of information on the validity of the exposure assessment. We have indicated that exposure was self-reported, and emphasized in the conclusion of the abstract that these results were observed on our sample, and need to be replicated in other studies.

The conclusion sentences should be revised to those in the past tense (e.g. is to was) so that the authors can avoid generalizing the study finding to the general fact.

+++ We have now replaced present tense with past tense.

In the method section, the authors should state no objective information on the validity measure.

+++ We have now stated in the methods section that we had no objective information on the validity measure. The following statement has been added to page 9:

*“**Despite the validation of dietary records against blood and urinary markers for energy and key nutrients, specific exposure to nitrates and nitrates has not been validated against blood or urine assays, given the challenge in identifying specific biomarkers reflecting exogenous dietary exposure and not endogenous metabolism. Therefore, no objective information on the validity, sensitivity, or specificity of the exposure assessment was available.”*

The authors should reduce their discussion on generalisability. The limitations of residual confounding and measurement errors mean that the result itself may be biased within the population. When the observation is biased, generalisability is the secondary concern.

In the discussion, the authors should clarify that validity, sensitivity, and specificity are unclear in their exposure assessment. The authors can keep the note about little availability of useful objective biomarkers, but should clarify that we must recognize the limitation.

In the discussion section, the authors may propose future validation study with a duplicate-meal design for multiple days sufficient to estimate habitual nitrate/nitrite intakes, for example.

Biomarkers from tissues would be just one of many ways to assess the validity of dietary exposure.

+++ We have now restructured the limitations section as follows: limitations of residual confounding and measurement errors are now presented as the main limitations (before generalizability), and further details on measurement errors, as well as perspectives on future validation studies as suggested by the Editor, have been provided. It now reads:

*“However, some limitations should be acknowledged. First, causation could not be established from this single observational study and residual confounding could not be entirely ruled out. Nevertheless, we adjusted for a wide range of socio-demographic, anthropometric, lifestyle, dietary and health factors in order to limit this potential bias. Second, even though dietary records were validated against blood and urinary biomarkers for energy and key nutrients, exposure to nitrates and nitrates has not been validated against blood or urine assays due to lack of biomarkers that would be specific enough to reflect exogenous dietary exposure and not endogenous metabolism. For instance, plasma nitrate would not only be a marker of dietary sources, but would also reflect nitrate derived from various individual (patho‐) physiological pathways, as well as the individual capacity to eliminate nitrate [45–47]; similarly for urinary nitrites, in addition to urinary tract infections being an important driver of urine nitrites levels [48], and could provide biased estimates of dietary exposure. Therefore, and despite the use of multiple databases and detailed dietary assessment, the validity of the exposure assessment cannot be guaranteed, the sensitivity and specificity are unclear.* *Although likely non-differential due to the prospective design, these potential measurement errors may have biased the associations towards an unclear direction. Future validations studies could be considered; for instance, using a duplicate-meal design for multiple days to estimate habitual nitrate/nitrite intakes, or biomarkers from tissues.”*

As requested, the discussion on generalizability has been reduced, pages 19-20.

Split the long paragraph about strengths and limitations into two.

+++ The paragraph on limitations has now been split into three subparagraphs.

Minor revisions:

Revise Figure 1 and 2. Avoid showing the pie charts. Change each into a bar chat that has bars which lengths correspond to the average total amounts of the consumption and which internal segments show different sources. Pie charts are generally discouraged to display in a scientific article.

+++ As requested, figures 1 and 2 have now been reformatted as follows:

Figure 1:

Figure 2:

Figure 4. "estimation" should be "Point estimates". "Estimation" is a noun meaning what the authors conducted, not what the authors produced. The underbar "_" should be removed.

The x-axis of the spline figure should have a unit. The abbreviation requires an explanation in the legend.

+++ Figure 4 has now been edited accordingly. In addition, two-segment Y-axes have now been used to comply with Reviewer #2’s comment.

Requests from Editors:

Line 80: Please update ‘made no difference’ to ‘did not distinguish’

+++ This has been modified accordingly.

The Data availability and Conflicts of interest sections can be removed from the main text – it is essential that all the relevant material is captured in the Data Availability Statement and Competing Interests sections of the submission form (this information will be published as metadata alongside your article).

+++ These sections have been removed and all information is captured in the Data Availability Statement and Competing Interests sections online.

Comments from Reviewers:

Reviewer #2: Regarding the revised-version of the manuscript entitled "Dietary exposure to nitrites and nitrates in association with type-2 diabetes risk: Results from the NutriNet-Santé population-based cohort study", although some of points have been addressed carefully, some issues need further clarifications.

+++ We thank Reviewer #2 for this new round of comments. All of them have been accounted for.

1-	The authors have tried to discuss the important limitation of the current study, i.e., lack of representativeness of the study population, resulted in a lower incidence rate of T2D compared to French population (1% compared to 6%), high-estimated nitrate-nitrite exposure from food sources, and low-estimated nitrate-nitrite exposure from additive sources. They ascertained that their findings could not be generalized to other populations, even French population as well, but their conclusion addresses to updating regulations regarding nitrites from food additives and lack of beneficial effect of dietary nitrate from natural sources. I think that the conclusion should be limited to their study population (young women, with healthy lifestyle, higher socio-professional and educational levels that have mostly home access to the Internet).

+++ We have now toned down the messages in our conclusion, by mentioning the generalizability (linked to overrepresented categories in our sample) and measurements issues (linked to the absence of biomarkers), as well as using the past tense and the conditional form. The conclusion now reads:

*“In this large prospective cohort, a higher exposure to foods and water-originated and additives-originated nitrites was associated with higher T2D risk. Thus, these results did not support any potential benefits for dietary nitrites or nitrates in T2D prevention. These findings need confirmation by other prospective studies and experimental research, given that the study sample overrepresented women, younger participants, and those having healthier lifestyle habits and higher socio-professional levels, and given the possible exposure measurement errors. Yet, these results provide a new piece of evidence in the context of current discussions regarding the need for a reduction of nitrite additives’ use in processed meats by the food industry, and could support the need for better regulation of soil contamination by fertilizers, as highlighted by the latest report of the French Agency for Food, Environmental and Occupational Health and Safety [56]. In the meantime, several public health authorities worldwide already recommend citizens to limit their consumption of foods containing controversial additives, among which sodium nitrite, in the name of the precautionary principle [57].”*

2-	Regarding T2D diagnosis, the authors reported that "Among the participants who provided blood sample during the clinical/biological examination, 232 had elevated fasting blood glucose (i.e. >1.26 g/L). Among them, 85.3% had consistently declared a T2D diagnosis and/or medication. Elevated blood glucose alone (i.e., without any declaration of T2D diagnosis or treatment) was not considered specific enough to classify the participant as a T2D case". It is not clear why they excluded about 15% of subjects with elevated blood glucose while according to IDF recommendation, fasting glucose higher than 126 mg/dL is a criteria for diagnosis of T2D.

+++ [The French guidelines of the High Authority of Health (*Haute Autorité de Santé*)](https://www.has-sante.fr/upload/docs/application/pdf/2015-02/7v_referentiel_2clics_diabete_060215.pdf) for T2D diagnosis require two biological measurements of fasting glucose higher than 1.26 g/L to define a T2D case. Our cohort being a French cohort, we used this definition as these are the guidelines that are followed in French clinical practice. We have now cited these guidelines in the S1-Appendix – Method C; the sentence now reads:

*“Elevated blood glucose alone (i.e., without any declaration of T2D diagnosis or treatment), especially if issued from a single biological measurement, was not considered specific enough to classify the participant as a T2D case, as per the guidelines of the French Haute Autorité de Santé (8).”*

3-	The results provided in response to reviewer 3 (comment 5) regarding possible association between nitrate-nitrite and risk of metabolic syndrome (i.e., significant association between total and food-originated nitrite with metabolic syndrome and lack of association with additive-originated nitrite) are in contrast with findings that provided for nitrite and T2D; how the authors explain this?

+++ As regards the analysis with metabolic syndrome showing a significant positive association with total and foods and water-originated but not with additives-originated nitrites, this model followed a cross-sectional design since biological measures were only available once; thus, the temporal sequence between the exposure and the outcome was not respected. These findings might be due to a reverse causation bias, as participants with a metabolic syndrome could have modified their diets by increasing fruit and vegetable consumption and reducing processed meats. This has been added to the discussion, page 17.

4-	As pointed with reviewer 3 and 4, the estimation of dietary nitrate/nitrite needs to be validated using biological measurements. Although the authors have tried to discuss this important limitation, this issue remains challenging.

+++ As explain in the answer to the Academic Editor (please see above), several edits and reformulations have now been performed in order to further stress this limitation.

5-	In response to the comment 11, regarding the incident rate of T1D, the authors provided a rate of 12.3 per 100000 person-year, but they discussed that "Since the outcome of interest was T2D for this study, these incident T1D cases were censored at the date of diagnosis". This sentence is not make sense; please clarify.

+++ T1D was considered as a competing risk; i.e., an event whose occurrence precludes the occurrence of T2D, our primary event of interest. In the cause-specific Cox model approach we used, we censored T1D cases at their date of T1D diagnosis; which means that these participants contributed to the follow-up until their date of T1D diagnosis, and they were censored and considered as non-cases. We have now clarified this sentence, page 10. It now reads:

*“Cause-specific Cox proportional hazards models with age as the primary timescale were used to study the associations between the exposure to nitrites and nitrates with T2D risk, with death and type-1 diabetes handled as competing events (i.e., an event whose occurrence precludes the occurrence of the primary event of interest). In observational cohort studies, these models do not allow to interpret data in terms of direct causal inference but are designed to account for competing risks [31]. Among cases, participants contributed person time until the date of T2D occurrence. Among non-cases, participants were censored at the date of last completed questionnaire, the date of diagnosis of an incident type-1 diabetes (88 cases, crude incidence rate 12.3 for 100,000 person-years, considered as “non-cases” for the present analysis), the date of death, or October 1st, 2021, whichever occurred first.”*

6-	The response to minor comment 1 "In the most Figures presented in the supplementary material, Y-axis is not started from zero. I strongly suggested that in all cases the start of Y-axis to be zero" is not acceptable. Authors can use two-segment Y-axis to overcome this challenge. It can be found for example in the GraphPad Prism software.

+++ We thank Reviewer #2 for providing an example of an adapted software. We have now modified the figure accordingly.

7-	Please changes the following sentence:

Original: In the mouth, about 5% of the ingested exogenous nitrate is reduced to nitrite by commensal bacteria residing in that area, and is then converted to nitric-oxide (NO) in the stomach, through the nitrate-nitrite-NO pathway, providing a continuous source of NO for human body, in addition to NO production through the L-arginine oxidative pathway.

Suggestion: About 25% of the ingested exogenous nitrate is reduced to nitrite by commensal bacteria residing in the mouth, of which about 20% is converted to nitric-oxide (NO) in the stomach, through the nitrate-nitrite-NO pathway (i.e., about 5% of ingested nitrate is converted to NO), providing a continuous source of NO for human body, in addition to NO production through the L-arginine oxidative pathway.

+++ Thank you. This sentence has been modified as requested.

Reviewer #3: I have read the revised version of the manuscript entitled "Dietary exposure to nitrites and nitrates in association with type-2 diabetes risk: Results from the NutriNet-Santé population-based cohort study". The manuscript has been revised carefully according to the reviewers' comments, however, I have some points provided below:

+++ We thank Reviewer #3 for these comments.

1-	In response to comment 1 & 2, the lack of representativeness of the study population have been discussed in the revised version, however, the conclusion remained unchanged. The authors could not extrapolate their results to other populations.

+++ As stated in the response to Reviewer #2, we have now toned down the messages in our conclusion, by mentioning the generalizability (linked to overrepresented categories in our sample) and measurements issues (linked to the absence of biomarkers), as well as using the past tense and the conditional form. The conclusion now reads:

*“In this large prospective cohort, a higher exposure to foods and water-originated and additives-originated nitrites was associated with higher T2D risk. Thus, these results did not support any potential benefits for dietary nitrites or nitrates in T2D prevention. These findings need confirmation by other prospective studies and experimental research, given that the study sample overrepresented women, younger participants, and those having healthier lifestyle habits and higher socio-professional levels, and given the possible exposure measurement errors. Yet, these results provide a new piece of evidence in the context of current discussions regarding the need for a reduction of nitrite additives’ use in processed meats by the food industry, and could support the need for better regulation of soil contamination by fertilizers, as highlighted by the latest report of the French Agency for Food, Environmental and Occupational Health and Safety [56]. In the meantime, several public health authorities worldwide already recommend citizens to limit their consumption of foods containing controversial additives, among which sodium nitrite, in the name of the precautionary principle [57].”*

2-	In response to comment 5, new analyses were performed to assess the possible associations between nitrate/nitrite and risk of metabolic syndrome, and the results indicated a significant association between total and food-originated nitrite with metabolic syndrome and lack of association with additive-originated nitrite. These findings differed from those reported for T2D; would you please explain the new findings?

+++ As stated in the answer to Reviewer #2, as regards the analysis with metabolic syndrome showing a significant positive association with total and foods and water-originated but not with additives-originated nitrites, this model followed a cross-sectional design since biological measures were only available once; thus, the temporal sequence between the exposure and the outcome was not respected. These findings might be due to a reverse causation bias, as participants with a metabolic syndrome could have modified their diets by increasing fruit and vegetable consumption and reducing processed meats. This has been added to the discussion, page 17.

Reviewer #4: Minor comment: Please correct 'Green leafy vegetables are the major source of dietary nitrates. Even though their role in reducing T2D risk has been suggested [1,2], the most recent meta-analyses [3-5] have reported low to very low quality of evidence for the association of incidence of cruciferous and green leafy vegetables with T2D risk' to 'Green leafy vegetables are the major source of dietary nitrates. Even though their role in reducing T2D risk has been suggested [1,2], the most recent meta-analyses [3-5] have reported low to very low quality of evidence for the association of cruciferous and green leafy vegetables with T2D risk'.

+++ We thank Reviewer #4 for this comment. The manuscript has been modified accordingly.

Reviewer #5: Thanks for the detailed responses to my comments/concerns. I think the authors have adequately addressed my and other reviewers' comments. I am happy with the revised version and would greenlight the paper for publication. Congratulations!

+++ We thank Reviewer #5 for agreeing to review our manuscript again, and for recommending its publication.

Any attachments provided with reviews can be seen via the following link:

__________________________________________________

In compliance with data protection regulations, you may request that we remove your personal registration details at any time. (Use the following URL: <https://www.editorialmanager.com/pmedicine/login.asp?a=r>). Please contact the publication office if you have any questions.
